# In silico designing of a novel epitope-based candidate vaccine against *Streptococcus pneumoniae* with introduction of a new domain of PepO as adjuvant

**DOI:** 10.1186/s12967-022-03590-6

**Published:** 2022-09-04

**Authors:** Zohreh Bahadori, Mona Shafaghi, Hamid Madanchi, Mohammad Mehdi Ranjbar, Ali Akbar Shabani, Seyed Fazlollah Mousavi

**Affiliations:** 1 Department of Medical Biotechnology, Faculty of Medicine, Semnan University of Medical Sciences, Semnan, Iran; 2 Research Center of Biotechnology, Semnan University of Medical Sciences, Semnan, Iran; 3 Department of Bacteriology, Pasteur Institute of Iran, Tehran, Iran; 4 Drug Design and Bioinformatics Unit, Department of Medical Biotechnology, Biotechnology Research Center, Pasteur Institute of Iran, Tehran, Iran; 5 Agricultural Research, Education, and Extension Organization (AREEO), Razi Vaccine and Serum Research Institute, Karaj, Iran

**Keywords:** *S.pneumoniae*, PspC, PhtD, PsaA, PepO, Protein-based vaccine, Toll-like receptor (TLR) agonist

## Abstract

**Background:**

*Streptococcus pneumoniae* is the leading reason for invasive diseases including pneumonia and meningitis, and also secondary infections following viral respiratory diseases such as flu and COVID-19. Currently, serotype-dependent vaccines, which have several insufficiency and limitations, are the only way to prevent pneumococcal infections. Hence, it is plain to need an alternative effective strategy for prevention of this organism. Protein-based vaccine involving conserved pneumococcal protein antigens with different roles in virulence could provide an eligible alternative to existing vaccines.

**Methods:**

In this study, PspC, PhtD and PsaA antigens from pneumococcus were taken to account to predict B-cell and helper T-cell epitopes, and epitope-rich regions were chosen to build the construct. To enhance the immunogenicity of the epitope-based vaccine, a truncated N-terminal fragment of pneumococcal endopeptidase O (PepO) was used as a potential TLR2/4 agonist which was identified by molecular docking studies. The ultimate construct was consisted of the chosen epitope-rich regions, along with the adjuvant role (truncated N-PepO) and suitable linkers.

**Results:**

The epitope-based vaccine was assessed as regards physicochemical properties, allergenicity, antigenicity, and toxicity. The 3D structure of the engineered construct was modeled, refined, and validated. Molecular docking and simulation of molecular dynamics (MD) indicated the proper and stable interactions between the vaccine and TLR2/4 throughout the simulation periods.

**Conclusions:**

For the first time this work presents a novel vaccine consisting of epitopes of PspC, PhtD, and PsaA antigens which is adjuvanted with a new truncated domain of PepO. The computational outcomes revealed that the suggested vaccine could be deemed an efficient therapeutic vaccine for *S. pneumoniae*; nevertheless*, *in vitro and in vivo examinations should be performed to prove the potency of the candidate vaccine.

**Supplementary Information:**

The online version contains supplementary material available at 10.1186/s12967-022-03590-6.

## Background

*Streptococcus pneumoniae* (pneumococcus), an opportunistic Gram + bacterium, resides normally on the throat or nasopharynx of healthy people and is transmitted through the carriers’ respiratory droplets [1]. It can cause life-threatening bacterial infections such as meningitis, sepsis, and pneumonia if it invades sterile regions of the body [2]. Moreover, pneumococcus is one of the leading causes of severe secondary infections following viral respiratory diseases such as coronavirus [3]. According to reports from the WHO, pneumococcal (Pnc) infections claimed the lives of 1.6 million people in both developed and developing nations in 2005, which happened a lot in the children < 2 and elderly ≥ 65 years [4]. Prevention of this considerable mortality rate on one side and progressing resistance of *S. pneumoniae* to existing antimicrobials on the other emphasize the critical need for an efficient vaccine that protects high-risk groups from the majority of clinically invasive serotypes. Presently, there are two types of polysaccharide pneumococcal vaccines on the market, unconjugated (plain) polysaccharide vaccines (PPV) and protein-conjugated polysaccharide vaccines (PCV) [5]. PPV comprising the T-cell independent polysaccharide antigens is ineffective in infants under two-year old who are at the highest risk for severe pneumococcal diseases. PCV protects children, however, it is costly, very difficult to manufacture, requires multiple injections, and needs refrigeration. Moreover, the PCV serotype coverage includes pneumococcal serotypes of developed countries [6, 7]. Both existing vaccines are serotype-dependent and only elicit serotype-specific immunity [7]. Because of the boundaries of available *S. pneumoniae* vaccines, the development of a powerful, broad-spectrum, and cost-effective vaccine that can be effective in preventing infections caused via different pneumococcal serotypes is a significant concern of World Health Organization.

Protein-based vaccine, comprising multiple conserved pneumococcal protein antigens, can give an alternative to serotype-dependent vaccines [8]. Understanding the different roles of virulence and colonization factors that induce pneumococcal infections is fundamental for developing the effective protein-based vaccines. Various pneumococcal proteins have been evaluated as possible vaccine candidates throughout the years, for instance, pneumococcal surface protein A and C (PspA/C) [9, 10], the members of pneumococcal histidine triad (Pht) family A, B, D, and E (PhtA, B, D, and E) [11, 12], and ATP-binding cassette (ABC) transporters such as PsaA, PiuA, and PiaA [13, 14]. Each of the above mentioned virulence proteins could produce various levels of protection against immunological challenges with numerous pneumococcal serotypes in animal model [7].

Pneumococcal surface protein C is one of the most important surface proteins of pneumococcus that is considered as a vaccine candidate [15]. PspC has been shown to have notable roles in adhering of pneumococcus, invading, and evading complement [10, 16]. Hence, it is also called factor H-binding inhibitor of complement (Hic) [17], C3-binding protein A (PbcA) [18], *S. pneumoniae*-secretory IgA-binding protein (SpsA) [19], and choline-binding protein A (CbpA) [20]. PspC consists of three domains: an exposed N-terminal (N-ter) α-helical domain, a proline-rich domain, and C-terminal (C-ter) repeat region [21]. This antigen shows variability among different serotypes and is classified into 11 groups [22]. The protein PspC as a recombinant vaccine candidate has been indicated to induce antibody-mediated protection against pneumococci [21, 23]. Investigations have revealed that anti-PspC3 (PspC from group 3) antibodies are capable to identify the majority of Pnc clinical isolates [24]. Pht family, which is a group of surface-associated pneumococcal antigens, contains 5 conserved His triad motifs (His-xx-His-x-His) in PhtA, B, and D, as well as 6 motifs in PhtE [12, 25]. A number of roles have been suggested for this family including involvement in the homeostasis of Zinc (Zn^2+^) which is crucial for the colonization and invasion of pneumococcus [26], and also complement deposition inhibition on the surface of pneumococcus via the recruitment of complement factor H [27]. Among the members of this family, PhtD has been determined to be the most conserved across different strains [28], and so to induce the broadest protection against pneumococcal diseases [29]. Researches have indicated that the C-terminal of PhtD (PhtD-C), compared to the other areas of the protein, is more surface-exposed and could be a more immunogenic part [28, 30]. Another protein candidate is pneumococcal surface adhesion A (PsaA) that has been assessed against Pnc infections with encouraging results in animal models and human clinical trials [31]. The PsaA antigen, a conserved lipoprotein expressed by all pneumococcal strains, is an important virulence factor of pneumococci [32, 33]. PsaA plays vital functions in manganese (Mn) transport [34] and adherence to the cells of the host [35]. In pneumococcus, the acquisition of Mn^2+^ has been found to have significant roles in growth, proliferation, and virulence [36].

Adjuvants are another component of vaccines that could significantly enhance the efficiency of vaccines and have been employed for more than a century [37]. In the past few decades, toll-like receptor (TLR) agonists have been extensively studied as possible adjuvants for vaccines [38]. Most of the TLR agonists currently being investigated are non-protein microbial products such as lipopeptides, oligonucleotides, and lipopolysaccharides [39]. On the other side, a developing number of studies have revealed there are various bacterial proteins that can activate TLR signaling and immune responses. Bacterial proteins that show one or more of the following features can be considered as potential adjuvant candidates: (1) induction of pro-inflammatory cytokines, (2) up-regulation of the costimulatory molecule expression on antigen presenting cells (APCs), (3) activation of the TLR2/TLR4 signaling, (4) induction of antibody- or cell-mediated immunity [39]. Microbial protein TLR agonists present unique characteristics that are not provided by non-protein TLR agonists, including the ability to modify the structure/function as needed for favorable immunogenicity and minimum toxicity [39]. To ensure the co-delivery of adjuvant and antigen into the target cells, protein TLR agonists could be genetically joined to protein antigens, leading to powerful stimulation of innate and acquired immune response [39]. *S. pneumoniae* endopeptidase O (PepO) is a TLR agonist that is able to induce a strong natural immune response in a TLR2/TLR4-dependent way [40, 41]. This protein which share homology with M13 peptidase family consists of two conserved domains: M13_N domain (residues 3–383) and M13 domain (residues 437–627). It has shown that the TLRs 2 and 4 bind to the Pnc endopeptidase O through the M13_N domain [40, 41].

Recent advances in cost- and time-efficient approaches of immunoinformatics and the availability of a broad array of tools for vaccine designing have notably allowed to develop new stable and accurate epitope vaccines [42–44]. Considering the feasibility and benefits of vaccines generated via immunoinformatics methods, the aim of this research was to employ these approaches to design an epitope-based vaccine against *streptococcus pneumoniae*. Herein, we used several virulence proteins of pneumococcus, i.e. PspC, PhtD, and PsaA, to predict B-cell and helper T-cell epitopes from them. The M13_N domain of PepO was subjected to molecular docking with TLR2/4 to determine the potential regions involved in the PepO-TLR interaction. Furthermore, molecular docking, molecular dynamics simulation, and in silico cloning were done on the final construct. The novelty of this study is the construction of an epitope-based vaccine containing more effective antigenic epitope-rich domains of three pneumococcal proteins, PspC, PhtD, and PsaA, which are capable of production of more diverse and robust responses. Another novelty of our study lies in the use of a new truncated domain of PepO as a TLR agonist adjuvant candidate for promoting the immunogenicity of the vaccine.

## Methods

**Fig. 1:**
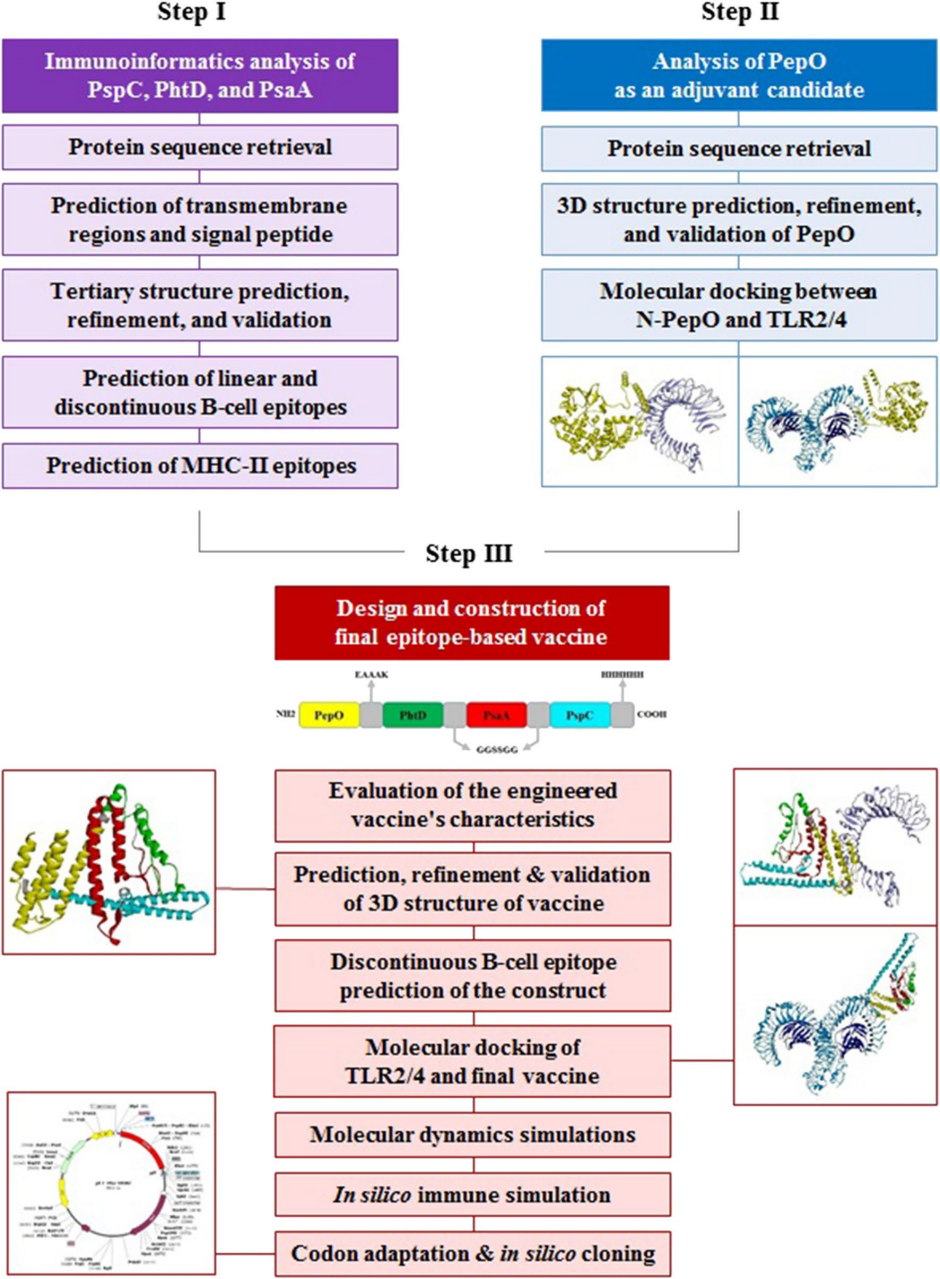
The workflow of this study for designing the epitope-based vaccine against pneumococcus. This research was carried out in three steps, step I, immunoinformatics analysis of the proteins PspC, PhtD, and PsaA for exploring epitope-rich locations. Step II, analysis of PepO as an adjuvant candidate for identifying of potential regions of PepO involved in the interaction with TLR2/4. Step III, design and construction of final epitope-based vaccine for developing a validated construct which could stimulate effective immunity against pneumococcal infections

This investigation was accomplished in three steps including the following: (1) immunoinformatics analysis of the proteins PspC, PhtD, and PsaA to predict the epitope-rich regions, (2) analysis of the PepO (as a TLR2/4 agonist) to identify its potential regions involved in the interaction with TLR, in order to be used as an adjuvant candidate, (3) design and construction of final epitope-based vaccine, and in silico cloning. Figure 1 depicts the process of this research.

## Immunoinformatics analysis

### Protein sequence retrieval and primary analysis

The sequences of PhtD (Accession no. AAK99711), PsaA (Accession no. AAF70663), and PspC from group 3 (PspC3, Accession no. EF424119) were fetched from the NCBI protein database (www.ncbi.nlm.nih.gov/protein). TMHMM Server v. 2.0 was employed to identify the transmembrane helices of proteins (www.cbs.dtu.dk/services/TMHMM-2.0) [45]. The SignalP 5.0 server was applied to detect the signal peptide region (http://www.cbs.dtu.dk/services/SignalP/) [46].

### Tertiary structure prediction of the selected proteins

Depending on the (un) availability of the structural template in the protein data bank (PDB), the template (free)-based modeling methods are used for the prediction of protein tertiary structure. In this study, the tertiary structures of PhtD and PspC were modeled by I-TASSER, a template-based method of modeling (https://zhanglab.ccmb.med.umich.edu/I-TASSER/) [47]. The I-TASSER suite, which automatically models 3D-structure of a given amino acid sequence, provides the model's ranking scores such as confidence score (C-score), template modeling (TM) score, and Root Mean Square Deviation (RMSD). The score of confidence ranges from -5 to + 2, with + 2 representing the most appropriate model. Template modeling score is in the range of 0–1, with closer to 1 representing the closeness of structure to global topology [48]. The score of RMSD indicates the similarity degree between a specific protein and the other. The model with a lower RMSD is more acceptable and realistic [49]. I-TASSER usually takes 1 ~ 2 days to produce a model depending on the protein size.

### Tertiary structure refinement and validation

The most desirable 3D structures modeled by the I-TASSER were refined through the GalaxyRefine server (http://galaxy.seoklab.org/cgi-bin/submit.cgi?type=REFINE) [50]. GalaxyRefine, which can improve the predicted structure's local and global quality, first reconstructs the side chains, and repacks them, and subsequent relaxes the overall structure using MD simulation. The average run time is usually 1 to 2 h. The server shows 5 modeled structures and respective their MolProbity, GDT-HA, Clash, Poor rotamers, RMSD along with Rama favored scores. The Z-score of ProSA server (https://prosa.services.came.sbg.ac.at/prosa.php) [51] and Ramachandran plot (R-Plot) of PROCHECK (https://servicesn.mbi.ucla.edu/PROCHECK/) [52] were computed to assess the correctness of the projected model and the effectiveness of the refinement process. The Z-score represents how similar the models are to native proteins, while the R-plot depicts the distribution of residues in most favored, allowed, and disallowed zones.

### Prediction of B-cell epitopes

A multi-method strategy was used to identify more dependable B-cell epitopes. The sequential B-cell epitopes of the antigens PhtD, PsaA, and PspC were predicted through machine learning-based algorithms such as ABCpred (http://crdd.osdd.net/raghava/abcpred/) [53], and LBTope (http://crdd.osdd.net/raghava/lbtope/) [54], and also physicochemical-based algorithms e.g. IEDB Emini tool (http://tools.iedb.org/bcell/result/) [55], and Ellipro (http://tools.iedb.org/ellipro/) [56]. Beside the high accuracy of the epitope prediction servers, one of their important features is their short response time (less than 5 min). ABCpred server is one of the first servers on the basis of the recurrent neural network, which generates epitopes with the length in the range of 12–20-mers. The LBtope server has an accuracy of around 81%. IEDB Emini tool was used to predict the surface accessibility of epitopes. The servers were applied at default settings.

Likewise, conformational B-cell epitopes were predicted based on the tertiary structures of the considered antigens using the servers Ellipro and DiscoTope (http://tools.iedb.org/discotope/) [57]. The modeled structures using the I-TASSER server and the crystal structure of PsaA (PDB id: 1PSZ) retrieved from RCSB (https://www.rcsb.org/) were used as inputs of these predictor servers.

### Prediction of MHC-II epitopes

T cell epitope prediction tool of the IEDB web server (http://tools.immuneepitope.org/mhcii) [58] and NetMHCIIpan 4.0 (http://www.cbs.dtu.dk/services/NetMHCIIpan/) [59] were used to discover appropriate MHC-II binding epitopes. In IEDB, each projected epitope has a percentile rank that indicates how well the epitope binds to MHC-II. The lower percentile rank is the better rank. We chose the eight HLA-DR1 alleles (DRB1*01:01, DRB1*03:01, DRB1*04:01, DRB1*07:01, DRB1*08:01, DRB1*11:01, DRB1*13:01, and DRB1*15:01) as the most common human alleles for MHC-II [60], and also the 3 mouse alleles (H2-IAb, H2-IAd, and H2-IEd).

## Determination of the potential regions involved in PepO-TLR interaction by molecular docking

### Sequence retrieval and structure modeling of PepO

The sequence of PepO (Accession no. ABJ55398) was fetched from the NCBI protein database. Since the tertiary structure of Pnc endopeptidase O was not available in PDB, the 3D structure of its M13_N domain was modeled and refined through the I-TASSER and GalaxyRefine servers, respectively.

### Molecular docking between N-PepO and TLR2/4

The protein PepO is able to enhance macrophage unspecific phagocytosis and bactericidal effect, depending on interactions of its N-terminal with the toll-like receptors 2 and 4. For a more detailed understanding of the interaction of N-Pepo with TLR2/4 and prediction of the amino acid residues involved in the interacting interface, molecular docking was performed through the ClusPro v2.0, a totally automated protein–protein docking server (https://cluspro.org/login.php) [61]. The average time used for the docking run is about 4 h, depending upon the size of the submitted proteins. This server conducts the following computing steps: rigid-body docking, clustering of the lowest energy conformations, and refinement of chosen complexes through energy minimization. For this docking analysis, 3-D structure of N-PepO was modeled using I-TASSER (as mentioned above) and the crystal structures of human TLR2 (PDB code: 6NIG) and TLR4 (PDB code: 3FXI) were retrieved from the RCSB site. The best docked complexes of the N-PepO-receptor with the lowest energy were chosen. To display and investigate the considered complexes, the Discovery Studio (DS) Visualizer and DIMPLOT tool from LigPlot + v2.2.4 [62] were used.

## Design and construction of epitope-based vaccine

On the basis of the predicted findings of the used servers, B cell and T cell epitopes with high scores were considered for generating an epitope-based vaccine. To keep and increase the immunogenicity of the chosen epitopes, a suitable pattern of them should be linked together employing appropriate linkers. The GGSSGG flexible linker was introduced between the proper epitopes of PhtD, PsaA, and PspC to maintain their independent folding and immunological activities [63]. The chosen sequence of the N-PepO as a potential adjuvant candidate was connected to the N-terminal of the above mentioned sequence with the EAAAK helical linker [64, 65] to promote the immunogenicity of the construct. In the end, a hexa-histidine (His6) tag was attached to the C-terminal of the engineered construct to aid the purification.

### Evaluation of the engineered vaccine's characteristics

The antigenicity potential of the candidate construct was investigated via the VaxiJen server (http://www.ddg-pharmfac.net/vaxijen/VaxiJen/VaxiJen.html) [66] with a threshold of 0.4 and the ANTIGENpro tool from the Scratch Protein Predictor server (http://scratch.proteomics.ics.uci.edu/) [67]. To predict allergenicity of the designed construct, AlgPred (https://www.imtech.res.in/raghava/algpred/) [68] and AllerTOP (http://www.ddg-pharmfac.net/AllerTOP/) [69] servers were utilized. The SOLpro server at (https://scratch.proteomics.ics.uci.edu/) [70] was employed for predicting the solubility of the vaccine upon overexpression in *Escherichia coli*. Toxicity of the primary structure of the construct was predicted through the ToxinPred server (https://webs.iiitd.edu.in/raghava/toxinpred/index.html) [71]. The Protparam tool from ExPasy web server (https://web.expasy.org/protparam/) was applied for determining theoretical isoelectric point value, molecular weight, half-life, aliphatic index, instability index, and other physiochemical properties of the designed construct [72]. Tools such as VaxiJen, AlgPred, AllerTOP, ToxinPred, and Protparam are very fast and can predict in a few seconds, while other tools such as ANTIGENpro and SOLpro are slower and require about half an hour.

### Prediction, refinement, and validation of the construct's tertiary structure

The designed construct's 3D structure was modeled via Robetta server (http://robetta.bakerlab.org/) [73]. The server takes about 4 to 6 h to run a short query of 150 residues. The best three-dimensional model was chosen and refined through GalaxyRefine server. Validating the model is a key step for identifying potential errors in the primary model and comparing the quality of the primary and refined models. The geometry, stereochemistry, and other structural aspects of the models were compared through Ramachandran plot, ProSA Z.score, and ERRAT (https://servicesn.mbi.ucla.edu/ERRAT/). R-Plot checked the stereochemical and geometrical restrictions of the construct. The ProSA server computed a Z-score to represent the construct structure's overall quality. The non-bonded atomic interactions were analyzed via ERRAT [74]. A higher ERRAT score represents a more trustworthy model for future investigation.

### Prediction of discontinuous B-cell epitopes of the construct

Following the three dimensional modeling of designed construct, prediction of its conformational B-cell epitopes which is a key task in designing of vaccine was performed via Ellipro tool of the IEDB server (http://tools.iedb.org/ellipro/) [56].

### Analysis of the designed vaccine's molecular interactions with immune receptors

The ClusPro online server was applied to carry out molecular docking between the engineered construct and toll-like receptors (TLR2 and TLR4). ​The 3D structure of the vaccine modeled by the Robetta server and the crystal structures of human TLR2/4 were used as the inputs for the docking processes. The rest of the steps were conducted similarly to the above explained docking between N-PepO and TLR2/4, and finally the best complexes were chosen.

### Molecular dynamics (MD) simulation

After conducting the docking and determining the finest orientation of the designed vaccine and receptor for interaction with each other, the 2 top-scored complexes were presented to the simulations of MD in 2 separate runs. The MD simulation analysis was performed for 50 ns to compute the docked complex's stability using the GROMACS program [75] with GROMOS96 54A7 force field [76]. The MD run was performed in about two days for each selected complex, using the described conditions in our prior works [77, 78]. Briefly, the target complexes were placed within a proper cubic box, solvated applying the TIP3P water type, and neutralized by adding sufficient Na + /Cl ions. The solvated systems were energy-minimized through the steepest descent method and equilibrated under NVT and NPT. During the MD simulations, the values of RMS Deviation and RMS Fluctuation (RMSF) were calculated through the use of g_rms and g_rmsf, respectively.

### Immune system simulation

To further verify the immune response and immunogenic profile of the designed construct, a computational simulation of the immune system was performed utilizing the C-ImmSim server (https://kraken.iac.rm.cnr.it/C-IMMSIM/) [79]. The server simulates at the same time three mammalian immune system compartments including bone marrow, thymus, and tertiary lymphatic organs. It utilizes machine learning approaches and a Position Specific Scoring Matrix (PSSM) method to predict immune epitopes and immune interactions, respectively. Three injections of the engineered vaccine were administered at 4-week intervals [80], and all simulation settings were left to default with time steps set at 1, 84, and 168, where each time step is eight hours, and the first time step is injection at the time zero.

### Reverse translation and codon optimization of the designed construct

In order to express the engineered vaccine in *Escherichia coli* expression system, the Java Codon Adaptation Tool (JCat) server (http://www.jcat.de/) was applied to do reverse translation and codon optimization in a few seconds [81]. The amino acid sequence of designed construct was translated reversely to the DNA sequence by setting the codon usage of *E. coli* k12 host. Additional optimizations were chosen to avoid the restriction enzymes (RE) cleavage sites, rho-independent transcription termination, and prokaryote ribosome binding site. The output of JCat includes the percent of CG content and codon adaptation index (CAI) that may be utilized to evaluate the levels of protein expression. The sequence's CG content which has favorable effects on translation and transcription should be between 30 and 70 percent. CAI presents details on codon usage biases; the desired score of CAI is > 0.8 and ≤ 1.0. Ultimately, to ensure vaccine expression, the optimized sequence (or gene) was inserted into the pET-28a ( +) through SnapGene tool (https://www.snapgene.com/try-snapgene/) which provides a fast and easy way for in silico cloning.

## Results

## Immuno-informatics analyses

### Protein sequence retrieval

The protein sequences of PhtD, PsaA, and PspC were retrieved from the NCBI. The C-terminal of PhtD (PhtD-C, 383–853), PsaA, and PspC amino acid chains were applied in the project to predict T helper and B cell epitopes.

### Prediction of transmembrane regions and signal peptide

The transmembrane topology investigation showed that the mentioned antigens lacked any transmembrane helices (Additional file 1: Fig. S1). According to the results of the SignalP server, the selected proteins except PsaA did not have a signal peptide (Additional file 1: Fig. S2).

### Tertiary structure prediction, refinement, and validation

The 3D structures of PhtD-C and PspC obtained from I-TASSER showed better quality than the models generated by the other servers. Considering the C-score, the model number one of PhtD-C (with C-score: -0.64) and PspC (with C-score: -0.65) were selected as the most suitable 3D-structure among five generated models for each of the proteins. The selected models of PhtD-C and PspC were refined using GalaxyRefine (Additional file 1: Fig. S3). The structure quality of the models was improved following treatment with the server as represented via ProSA Z-score and Ramachandran plot. The results of validation of the protein models before and after refinement are presented in Additional file 1: Table S1. The ProSA server revealed that refined models of PhtD-C and PspC had the Z-score of -9.1 and -0.04, respectively, which are within the range of scores for similar size natural proteins (Additional file 1: Fig. S4 A and B). According to the assessment of the Ramachandran plot of refined models, there were 81.7%, 16.9%, and 1.4% residues of the PhtD-C, 91.5%, 6.6%, and 1.9% residues of the PspC in the most favored, allowed, and disallowed regions, respectively (Additional file 1: Fig. S4 a, b).

### Identification of linear and discontinuous B-cell epitopes

As B-cell epitopes have an essential role in humoral responses, the sequential and discontinuous B-cell epitopes of the mentioned proteins were predicted using LBTope, ABCpred, IEDB Emini tool, and Ellipro (for the sequential epitopes), Ellipro and DiscoTope servers (for discontinuous epitopes). The prediction results obtained by the servers are provided in Additional file 1: Tables S2–S7, the first 3 tables are for linear and the last 3 are for discontinuous B-cell epitopes.

### Identification of MHCII epitopes

T-helper epitopes from PhtD-C, PsaA, and PspC sequences were predicted using IEDB (percentile rank < 10) and NetMHCIIpan (%Rank ≤ 1). The different MHCII epitopes from the protein sequences were derived via the servers according to eight HLA-DRB1 alleles (DRB1*01:01, DRB1*03:01, DRB1*04:01, DRB1*07:01, DRB1*08:01, DRB1*11:01, DRB1*13:01, and DRB1*15:01) and three mouse alleles (H2-IAb, H2-IAd, and H2-IEd). The results of predictions are listed in Additional file 1: Tables S8–S10.

## Analysis of the interaction of N-PepO with TLR2/4

### Retrieval of sequence and modeling of 3D structure

The protein sequence of PepO was fetched from the NCBI. The tertiary structure of N-PepO obtained from I-TASSER showed better quality than the models generated through the other servers. The 3D model number one of N-PepO (with C-score: 1.42) was chosen as the most proper structure among the generated models. The selected model of N-PepO was refined using GalaxyRefine (Additional file 1: Fig. S5A). The results of validation before and after refinement represented that the structure quality of the model was improved. The ProSA server revealed that the refined model had the Z-score of -2.62, which is within the range of scores for similar size natural proteins (Additional file 1: Fig. S5B). According to the assessment of the Ramachandran plot of the refined model, there were 94.4%, 4.5%, and 1.1% residues in the most favored, allowed, and disallowed zones, respectively (Additional file 1: Fig. S5C).

### A more detailed identification of interaction of N-PepO with TLR2/4 by molecular docking

**Fig. 2:**
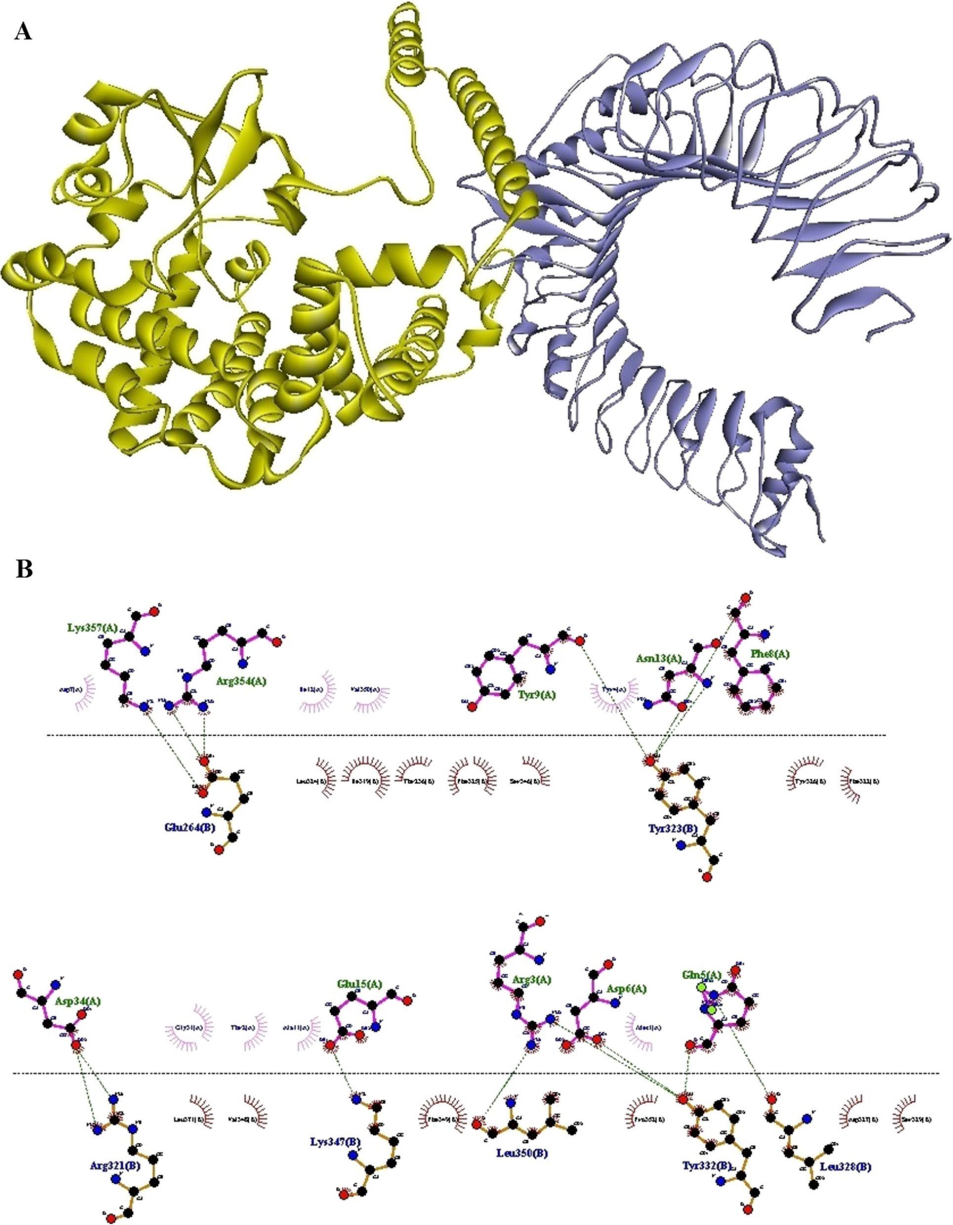
Docking output of the N-PepO and TLR2 receptor. **A** The 3D structure shows the interaction between TLR2 and PepO as the receptor and ligand represented in harbor blue and yellow, respectively. **B** The formation of hydrogen bonds between the Glu^264^, Arg^321^, Tyr^323^, Leu^328^, Tyr^332^, Lys^347^, and Leu^350^ of TLR2 and the Arg^3^, Gln^5^, Asp^6^, Phe^8^, Tyr^9^, Asn^13^, Glu^15^, Asp^34^, Arg^354^, and Lys^357^ residues of PepO is shown by Dimplot 2D-interaction plot. TLR2 residues, PepO residues, hydrogen bonds, and non-bonded residues are presented in blue, green, green dashed lines, and red/pink eyelashes, respectively

**Fig. 3:**
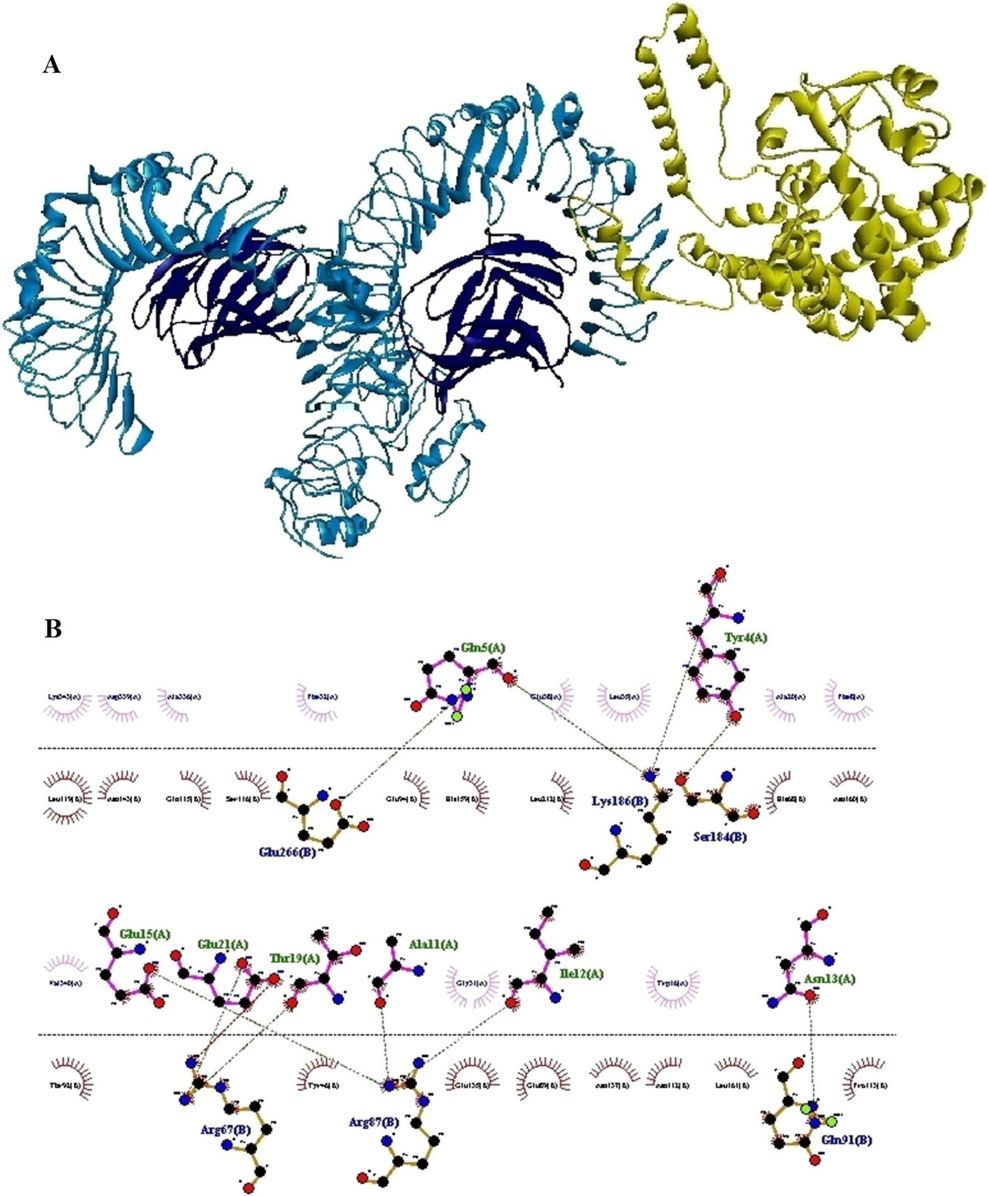
The result of docking of the N-PepO and TLR4 receptor. **A** The model demonstrates the interaction between PepO and TLR4 as the ligand and receptor depicted in yellow and blue, respectively. **B** Dimplot 2D-interaction plot depicts the creation of hydrogen bonds between PepO residues (Tyr^4^, Gln^5^, Ala^11^, Ile^12^, Asn^13^, Glu^15^, Thr^19^, and Glu^21^) and TLR4 residues (Arg^67^, Arg^87^, Gln^91^, Ser^184^, and Val^266^). PepO residues, TLR4 residues, hydrogen bonds, and non-bonded residues are shown in green, blue, green dashed lines, and red/pink eyelashes, respectively

To identify the residual interactions between N-PepO and TLR2/4, the refined modeled structure of N-PepO from the previous step and the crystal structures of TLRs 2 and 4 were docked by the ClusPro 2.0 online server. Following docking, it was seen that the N-PepO possessed good interaction with TLR2/4. The model.000.00 for N-PepO-TLR2 and model.000.02 for N-PepO-TLR4 were chosen with the lowest energy docking mode -859.4 and -967.1, respectively. The Discovery Studio Visualizer and DIMPLOT from LigPlot^+^v2.2.4 were used for visualizing and analyzing the chosen complexes (Figs. 2 and 3). The N-PepO-TLR2 complex formed 14 hydrogen bonds and 4 salt bridges, Arg^354^-Glu^264^, Lys^357^-Glu^264^, Asp^34^-Arg^321^, Phe^8^-Tyr^323^, Tyr^9^-Tyr^323^, Asn^13^-Tyr^323^, Gln^5^-Leu^328^, Arg^3^-Tyr^332^, Gln^5^-Tyr^332^, Asp^6^-Tyr^332^, Glu^15^-Lys^347^, and Arg^3^- Leu^350^ (Table 1). Comprehensive insights into intermolecular interactions of the N-PepO-TLR2 showed that all the hydrogen bonds except Asn^13^-Tyr^323^ formed at distances below 3 A° (Table 1). Likewise, the N-PepO-TLR4 complex made 10 hydrogen bonds and 2 salt bridges, Thr^19^-Arg^67^, Glu^21^-Arg^67^, Ala^11^-Arg^87^, Ile^12^-Arg^87^, Glu^15^-Arg^87^, Asn^13^-Gln^91^, Tyr^4^-Ser^184^, Tyr^4^- Lys^186^, Gln^5^-Lys^186^, and Gln^5^-Glu^266^. All the hydrogen bonds, excluding Ile^12^-Arg^87^, formed at distances less than 3 A° (Table 1). Considering the obtained results, a truncated fragment of N-PepO from amino acids 1 to 112, which was potentially involved in the interactions between N-PepO and TLRs, was selected as a potential TLR2/4 agonist candidate for further stage of the research.

**Table 1:** Interactions of the N-PepO with TLR2 and TLR4. Investigation of interactions was performed through DIMPLOT program from LigPlot^+^v2.2.4

N-PepO-TLR2						N-PepO-TLR4				
N-PepO residues		TLR2 residues			H-bond distance	N-PepO residues		TLR4 residues		H-bond distance
Name	No	Name		No		Name	No	Name	No	
Arg	354	Glu	264		2.74	Thr	19	Arg	67	2.79
Arg	354	Glu	264		2.71	Glu	21	Arg	67	2.84
Lys	357	Glu	264		2.75	Ala	11	Arg	87	2.77
Asp	34	Arg	321		2.73	Ile	12	Arg	87	3.06
Asp	34	Arg	321		2.69	Glu	15	Arg	87	2.78
Phe	8	Tyr	323		2.79	Asn	13	Gln	91	2.93
Tyr	9	Tyr	323		2.68	Tyr	4	Ser	184	2.97
Asn	13	Tyr	323		3.09	Tyr	4	Lys	186	2.59
Gln	5	Leu	328		2.95	Gln	5	Lys	186	2.59
Arg	3	Tyr	332		2.63	Gln	5	Glu	266	2.84
Gln	5	Tyr	332		2.75					
Asp	6	Tyr	332		2.60					
Glu	15	Lys	347		2.51					
Arg	3	Leu	350		2.66					

## Designing of epitope-based vaccine

Based on the results of the immuno-informatics investigation, epitopes of B-cell and MHC-II with the best scores were considered. The final construct of the vaccine consisting of four domains: the truncated N-PepO (1–112), PhtD-C (196–256), PsaA (100–187), and PspC (276–363) was designed. The chosen domains of PhtD, PsaA, and PspC were connected together via GGSSGG linkers. The selected fragment of N-PepO as a potential candidate adjuvant was joined to the N-terminal of the considered domains with the EAAAK linker. Following attachment of the His6 (HHHHHH) tag to the C-terminal of the construct, the epitope-based vaccine (ODAC) was finally designed to be 372 residues in length. The ODAC sequence is as follows:

MTRYQDDFYDAINGEWQQTAEIPADKSQTGGFVDLDQEIEDLMLATTDKWLAGEEVPEDAILENFVKYHRLVRDFDKREADGITPVLPLLKEFQELETFADFTAKLAEFELAEAAAKAAQAYAKEKGLTPPSTDHQDSGNTEAKGAEAIYNRVKAAKKVPLDRMPYNLQYTVEVKNGSGGSSGGSDGVDVIYLEGQNEKGKEDPHAWLNLENGIIFAKNIAKQLSAKDPNNKEFYEKNLKEYTDKLDKLDKESKDKFNKIPAEKKLIVTSEGGSSGGRRNYPSNTYFSLELEISESDVEVKKAEFELVKEEAKEPRNEEKVKQAKAKVESKKAVATRLENIKTDRKKAEEEAKRKAAEEDKVKEKGHHHHHH.

**Fig. 4:**
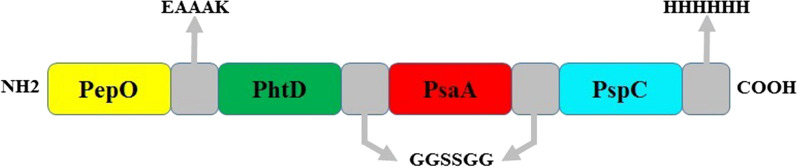
Schematic diagram of the candidate construct. The epitope‑rich domains of PhtD, PsaA, and PspC are shown in green, red, and turquoise blue, respectively, which are linked together with GGSSGG. The truncated fragment of PepO shown in yellow as a potential adjuvant candidate was joined to the N-terminal of the considered domains through the EAAAK linker. A hexa-histidine (His6) tag was fused at the C-terminal of the candidate vaccine to facilitate purification

The details of the chosen epitopes in the designed construct and schematic diagram of the vaccine are presented in Table 2 and Fig. 4.

**Table 2:** Details of the chosen domains of PhtD, PsaA and PspC for designing the final construct

Protein	PhtD-C	PsaA	PspC
Final selected domain	(Residues 196–256) AAQAYAKEKGLTPPSTDHQDSGNTEAKGAEAIYNRVKAAKKVPLDRMPYNLQYTVEVKNGS	(Residues 100–187) SDGVDVIYLEGQNEKGKEDPHAWLNLENGIIFAKNIAKQLSAKDPNNKEFYEKNLKEYTDKLDKLDKESKDKFNKIPAEKKLIVTSEG	(Residues 276–363) RRNYPSNTYFSLELEISESDVEVKKAEFELVKEEAKEPRNEEKVKQAKAKVESKKAVATRLENIKTDRKKAEEEAKRKAAEEDKVKEK
Final B-cell epitopes	224–238 225–239 226–240 234–248 237–251 238–252 239–253 240–255 241–256	103–117 104–118 105–119 106–120 107–121 108–122 109–123 110–124 113–127 133–147 141–155 148–162	285–299 288–302 294–308 295–309 324–339 327–341
Final MHC-II Epitopes (Mouse)	189–203 190–204 217–231 218–232 223–237 224–238 225–239 226–240 227–241	174–188 175–199 176–190	288–302 289–303 290–304 298–312 299–313 300–314 301–315 316–330 333–347 323–337 322–336 321–335 324–338 320–334
Final MHC-II epitopes (Human)	222–236 223–237 224–238 225–239 227–241 228–242 240–254 241–255	103–117 104–118 126–140 167–181 168–182 169–183	303–317 334–348 335–349 336–350 337–351 333–347

### Evaluation of the sequence properties of the designed vaccine

The sequence antigenicity probability was estimated to be 0.748 and 0.926 using the VaxiJen and the ANTIGENpro, respectively. The engineered sequence was predicted to be non-allergenic by Alg Pred and Allertop online servers. According to SOLpro results, the solubility of the candidate vaccine upon overexpression in *Escherichia coli* was 0.893. ToxinPred was employed for predicting the toxicity of the designed sequence and it was deemed as non-toxic. Furthermore, the ProtParam results indicated that theoretical isoelectric point value (pI) and molecular weight (Mw) of the construct were 5.29 and 41.939 kDa, respectively. Half-life was computed to be 30 h in mammalian reticulocytes, > 20 h in yeast, and more than 10 h in *E. coli*. Aliphatic index (AI) and grand average of hydropathicity (GRAVY) values were defined as 68.01 and -0.954, respectively. The instability index (II) of the designed sequence was estimated to be 36.83, indicating that it is stable. The total number of positive (+ R) and negative (-R) residues were computed 60 and 70, respectively. The details of the sequence properties of the designed vaccine are presented in Table 3.

**Table 3:** Analysis of sequence characteristics of the ODAC construct. Physicochemical parameters, antigenicity, solubility and toxicity of the sequence were computed through Expasy’s ProtParam, ANTIGENpro/VaxiJen, SOLpro and Toxin Pred, respectively. Also, the allergenicity of the desired sequence was evaluated using AllerTOP and AlgPred

Protparam tool											Vaxi Jen	ToxinPred	Aller TOP	Alg Pred	Scratch protein predictor	
Sequence Length	M.wt	+ R	− R	pI	Half-life in			II	AI	GRAVY					SOL pro	Antigen pro
					Mammalian	Yeast	*E. coli*									
372	41.93	60	75	5.29	30 h	> 20 h	> 10 h	36.83	68.01	-0.954	0.748	Non toxin	Non allergen	Non allergen	0.89	0.92

### Prediction, refinement, and validation of tertiary structure of the vaccine

**Fig. 5:**
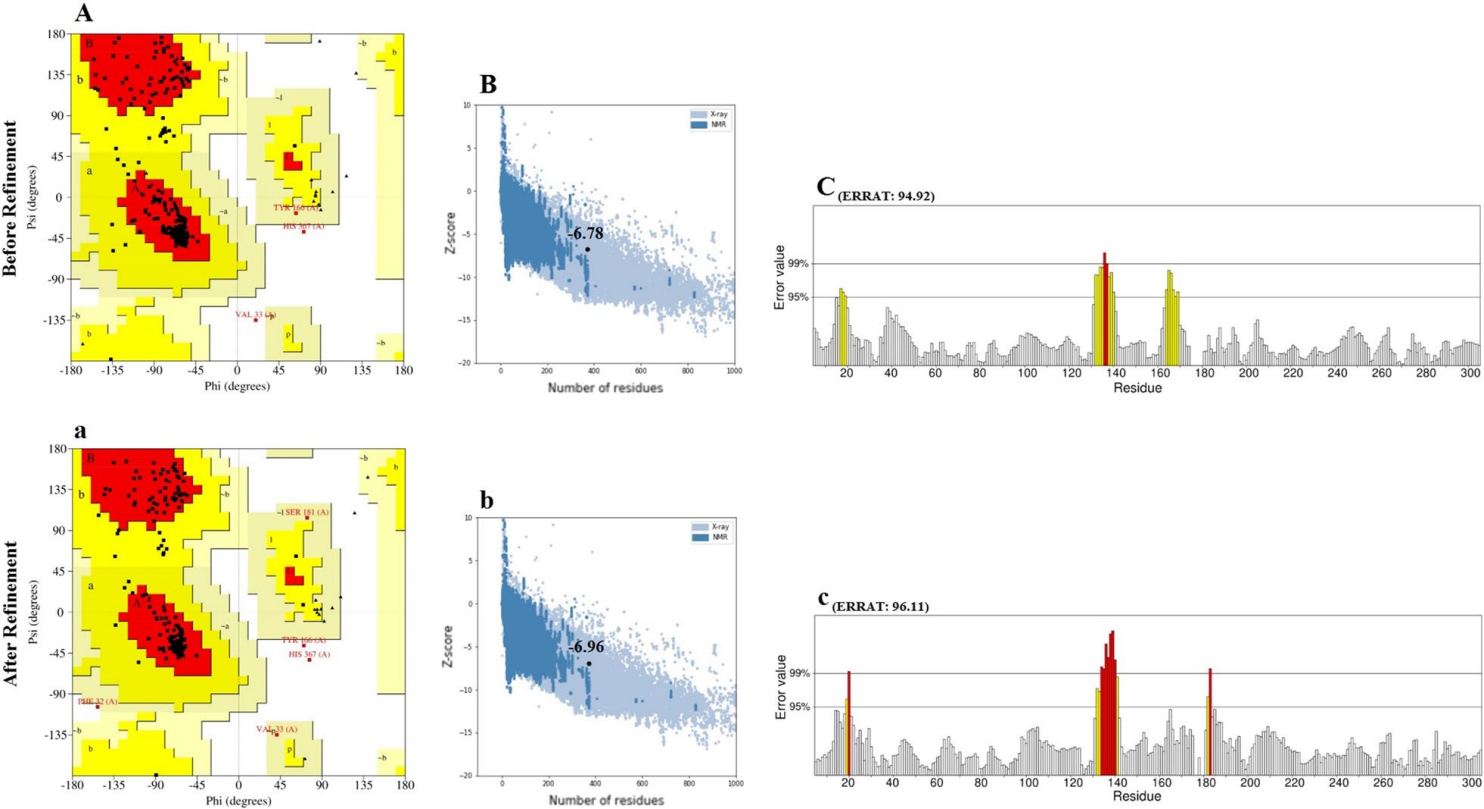
The comparison of the unrefined and refined vaccine. **A**(**a**), **B**(**b**), and **C**(**c**) present the R-plot, ProSA Z-score, and ERRAT, respectively. Following refinement of the chosen model through Galaxy Refine, the vaccine properties were improved (**a**–**c**)

**Fig. 6.**
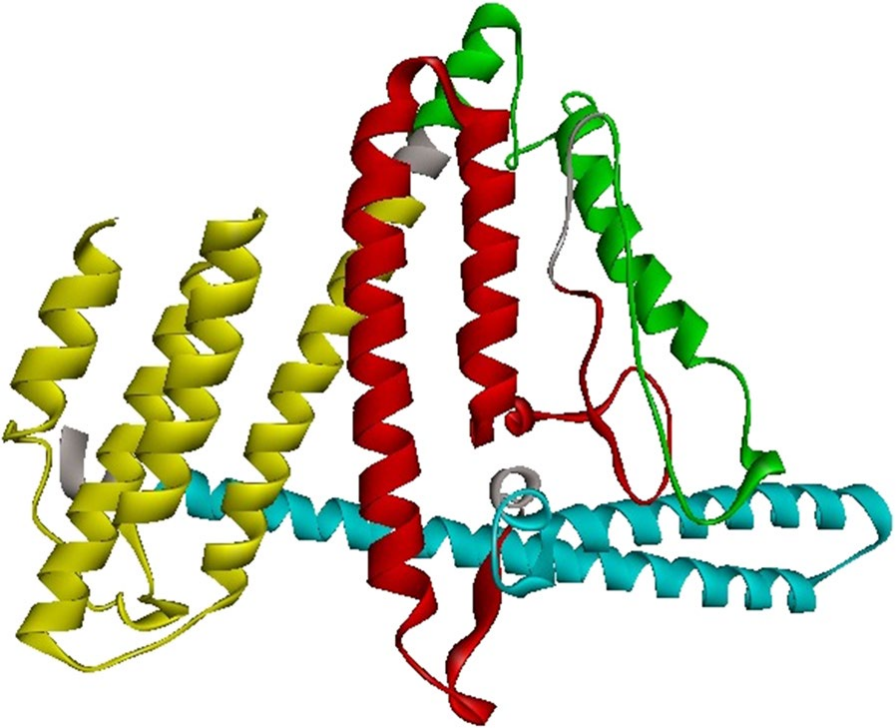
3-Dimensional structure of the engineered vaccine was displayed by Discovery Studio Visualizer. The PepO, PhtD, PsaA, and PspC regions and linkers are shown in yellow, green, red, turquoise, and gray colors, respectively

Five 3-dimensional structures of the designed vaccine sequence were generated by the Robetta server, and the best model was chosen. The R-plot revealed that 92.5, 6.9, and 0.6 percent of amino acids were located in the favored, allowed, and disallowed zones (Fig. 5A). Moreover, the ProSA Z-score of -6.78 was inside the area of scores for natural proteins, confirming the overall quality and reliability of the designed model (Fig. 5B). The ERRAT score of the chosen model was 94.92 percent (Fig. 5C). Following the refinement of the structure using Galaxy Refine, the model properties were improved. The primary and the refined structures are compared in Fig. 5 and Table 4. The R-plot of the refined model showed that 94.00, 5.1, and 0.9 percent of residues are located in favored, allowed, and disallowed zones, respectively (Fig. 5a). Furthermore, the Z-score (Fig. 5b), and ERRAT score (Fig. 5c) were improved to -6.96 and 96.11 in the refined model, respectively (Table 4). The 3-dimensional structure of the refined model was visualized via Discovery Studio Viewer (Fig. 6).

**Table 4:** Evaluation of the modeled vaccine before and after refinement. Analysis of the models was performed by the PROCHECK Ramachandran plot, ERRAT and ProSA-web Z-score

Step	Ramachandran plot			ERRAT	Z-score
	Most favored regions (%)	Allowed regions (%)	Disallowed regions (%)		
Before refinement	92.5	6.9	0.6	94.92	− 6.78
After refinement	94.00	5.1	0.9	96.11	− 6.96

### Discontinuous B-cell epitope prediction of the engineered construct

Given the significance of conformational epitopes in immunization, discontinuous B cell epitopes of the 3D structure of engineered construct were predicted based on the interaction of protein and antibody using the Ellipro server. Conformational peptides with a score of > 0.5 were chosen, and the scores showed the surface atoms of the construct that are responsible for antibody binding. Table 5 summarizes the number of amino acids, the amino acid compositions, the score values, and the sequence location.

**Table 5:** Prediction of discontinuous B-cell epitopes of ODAC vaccine. The conformational epitopes were predicted using ElliPro

Epitopes	Residues	Number of residues	Score
1	S296, E299, V300, K301, K302, A303, E304, F305, E306, L307, V308, K309, E310, E311, A312, K313, E314, P315, R316, N317, E318, E319, K320, V321, K322, Q323, A324, K325, A326, K327, V328, E329, S330, K331, K332, A333, V334, A335, T336, R337, N340, I341, D344, K347	44	0.819
2	M1, T2, R3, Y4, Q5, D6, D7, Y9, D10, A11, I12, N13, G14, E15, W16, Q17, Q18, T19, A20, E21, I22, P23, A24, D25, K26, S27, Q28, T29, G30, G31, F32, V33, D34, P57, E58, H69, R70, V72, R73, D74, F75, D76, K77, R78, E79, A80, D81, G82, I83, T84, P85, V86, A357, E358, E359, K361, V362, K363, E364, K365, G366, H367, H368, H369, H370, H371, H372	67	0.724
3	L51, A52, G53, E54, A114, A115, A116, K117, A118, A119, Q120, A121, Y122, A123, K124, E125, K126, G127, L128, T129, P130, P131, S132, T133, D134, H135, Q136, D137, S138, G139, N140, T141, E142, A143, K144, G145, E147, V174, K175, N176, G177, S178, G179, G180, S181, S182, S225, A226, K227, D228, P229, N230, N231, K232, E233, F234, E236, K237	58	0.715
4	V159, P160, D162, R163	4	0.595
5	D255, N258, K259	3	0.591
6	I260, P261, A262, E263, K264, K265, P282, S283	8	0.579

### Molecular docking of TLR2/4 and ODAC vaccine

**Fig. 7:**
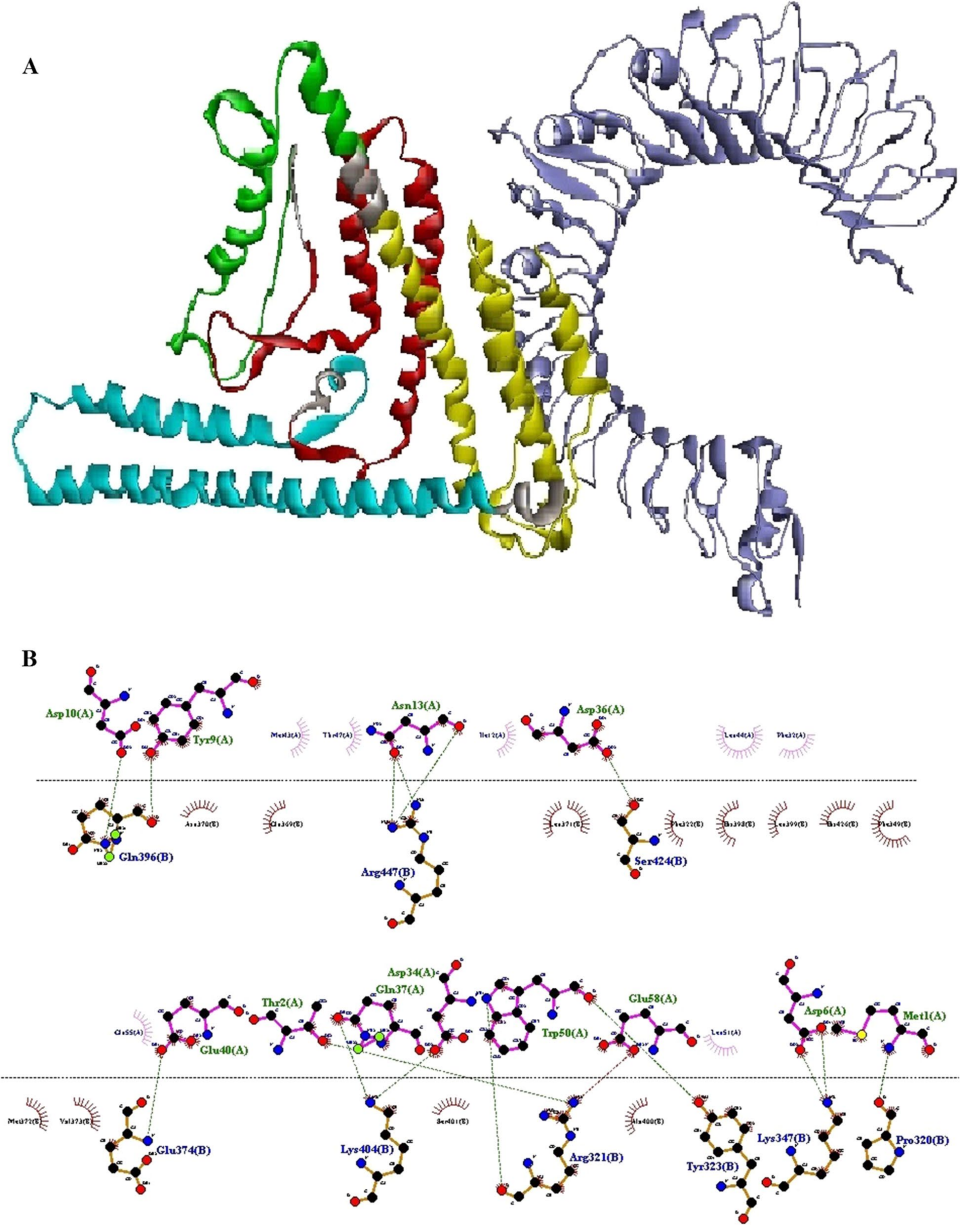
The outcome of docking of the vaccine construct and TLR2 receptor. **A** The three-dimensional structure illustrates the interaction of the construct and TLR2 that are shown in colored parts and harbor blue, respectively. TLR2 and vaccine interacted with each other via the truncated N-PepO (yellow part). **B** The Dimplot 2D-interaction plot demonstrates the formation of hydrogen bonds between the construct residues (Met^1^, Thr^2^, Asp^6^, Tyr^9^, Asp^10^, Asn^13^, Asp^34^, Asp^36^, Gln^37^, Glu^40^, Trp^50^, and Glu^58^) and TLR2 residues (Pro^320^, Arg^321^, Tyr^323^, Lys^347^, Glu^374^, Gln^396^, Lys^404^, Ser^424^, and Arg^447^). PepO residues, TLR2 residues, hydrogen bonds, and non-bonded residues are indicated in green, blue, green dashed lines, and red/pink eyelashes, respectively

**Fig. 8:**
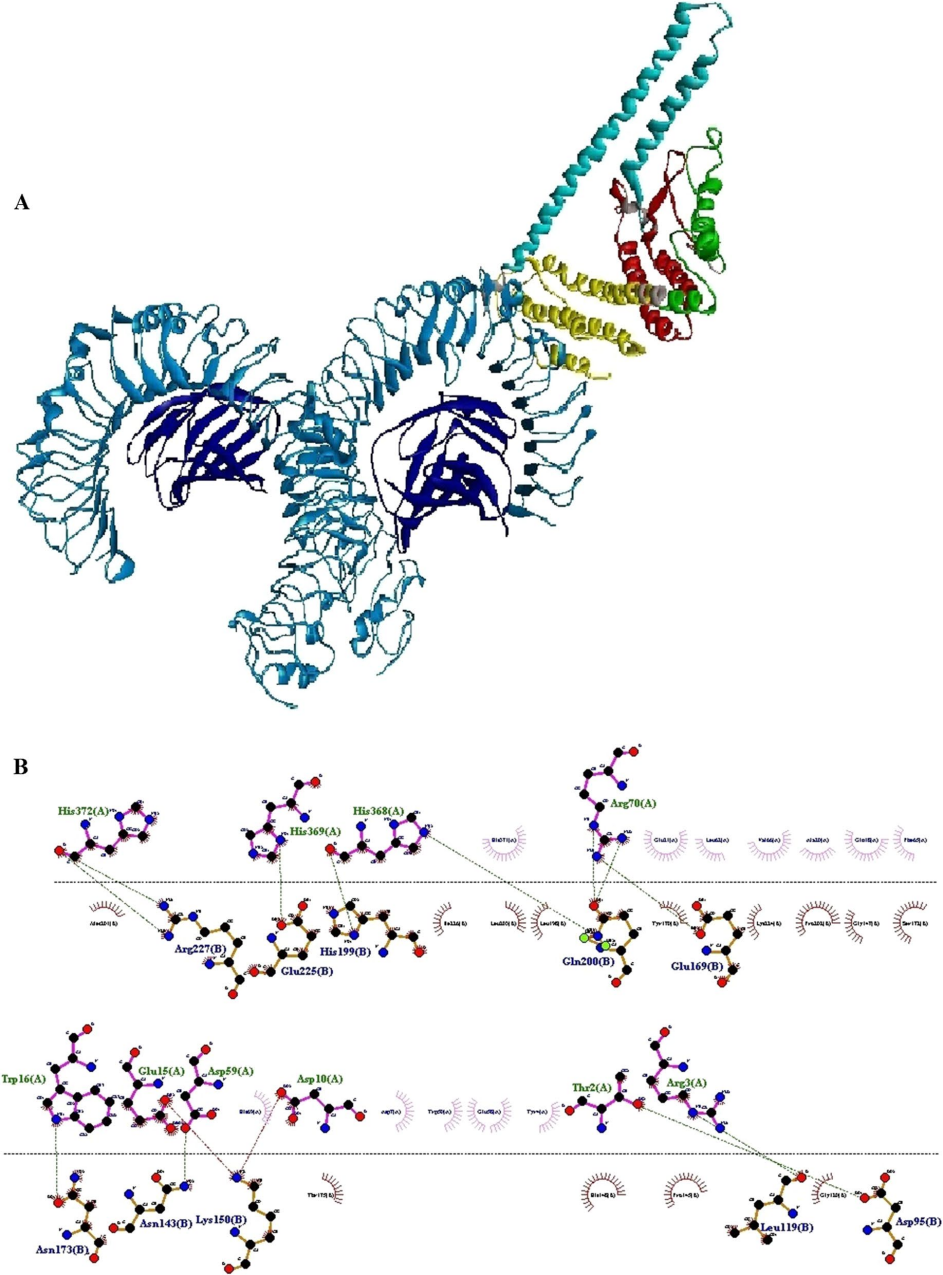
Molecular docking of the vaccine with TLR4 receptor. **A** The complex shows the interaction between the engineered construct and TLR4 that are represented in colored parts and blue, respectively. TLR4 and ODAC interacted with each other by the truncated N-PepO (yellow part). **B** The Dimplot 2D-interaction plot shows the creation of hydrogen bonds between the designed construct residues (Thr^2^, Arg^3^, Asp^10^, Trp^16^, Asp^59^, Arg^70^, His^368^, His^369^, and His^372^) and TLR4 residues (Asp^95^, Leu^119^, Asn^143^, Lys^150^, Glu^169^, Asn^173^, His^199^, Gln^200^, Glu^225^, Arg^227^). TLR4 residues, construct residues, hydrogen bonds, and non-bonded residues are depicted in blue, green, green dashed lines, and red/pink eyelashes, respectively

As mentioned above, the first 112 residues of the N-PepO may act as a potential binding site for TLR2/4. The construct of the vaccine was engineered in an effective way to interact with the TLR2 and TLR4 receptors. The process of protein–protein docking between TLR2/4 receptor and vaccine was performed by Cluspro 2.0 online server after eliminating the ligand and water molecules from the crystallographic structure of receptor. Several complexes were generated by the docking, and it was observed that the engineered vaccine could interact well with the TLR2/TLR4. The model.000.00 and model.000.01 were chosen for ODAC-TLR2 and ODAC-TLR4 complexes with the lowest energy docking mode -922.1 and -798.3, respectively. For displaying and evaluating the considered complexes, the DS Visualizer and DIMPLOT tools from LigPlot + v2.2.4 were used (Figs. 7 and 8). According to a detailed review of the results, the designed vaccine and TLR2/4 interacted properly via the truncated region of N-PepO. The engineered construct-TLR2 formed 15 hydrogen bonds and 3 salt bridges, Met^1^-Pro^320^, Trp^50^-Arg^321^, Thr^2^-Arg^321^, Trp^50^-Tyr^323^, Asp^6^-Lys^347^, Glu^40^-Glu^374^, Tyr^9^-Gln^396^, Asp^10^-Gln^396^, Asp^34^-Lys^404^, Gln^37^-Lys^404^, Asp^36^-Ser^424^, Asn^13^-Arg^447^, and Glu^58^-Arg^321^. Analysis of ODAC-TLR2 intermolecular interaction revealed that only Glu40-Glu374 hydrogen bond formed at distance above 3 A° (Table 5). Also, the designed vaccine-TLR4 made 12 hydrogen bonds and 4 salt bridges, Thr^2^-Asp^95^, Arg^3^-Leu^119^, Asp^59^-Asn^143^, Arg^70^-Glu^169^, Trp^16^-Asn^173^, His^368^-His^199^, Arg^70^-Gln^200^, His^368^-Gln^200^, His^369^- Glu^225^, His^372^-Arg^227^, Asp^10^-Lys^150^, and Glu^15^-Lys^150^. Investigation of ODAC-TLR4 intermolecular interaction exhibited that all the hydrogen bonds formed at distances between 2.59 and 2.99 A° (Table 6). Both chosen complexes were subjected as the initial structures in MD simulation processes.

**Table 6:** Interactions of the engineered vaccine with TLR2 and TLR4. Analysis of interactions was performed by DIMPLOT program from LigPlot + v2.2.4

ODAC -TLR2					ODAC -TLR4				
ODAC residues		TLR2 residues		H-bond distance	ODAC residues		TLR4 residues		H-bond distance
Name	No	Name	No		Name	No	Name	No	
Met	1	Pro	320	2.88	Thr	2	Asp	95	2. 90
Trp	50	Arg	321	2.97	Arg	3	Leu	119	2.83
Thr	2	Arg	321	2.61	Asp	59	Asn	143	2.91
Trp	50	Tyr	323	281	Arg	70	Glu	169	2.78
Asp	6	Lys	347	2.64	Trp	16	Asn	173	2.86
Asp	6	Lys	347	2.54	Arg	70	Gln	200	2.77
Glu	40	Glu	374	3.08	Arg	70	Gln	200	2.62
Tyr	9	Gln	396	2.74	His	368	Gln	200	2.99
Asp	10	Gln	396	2.75	His	369	Glu	225	2.78
Asp	34	Lys	404	2.75	His	372	Arg	227	2.84
Gln	37	Lys	404	2.77	Asp	10	Lys	150	2.64
Asp	36	Ser	424	2.82	Glu	15	Lys	150	2.71
Asn	13	Arg	447	2.69					
Asn	13	Arg	447	2.71					
Glu	58	Arg	321	2.69					

### MD Simulations of selected complexes

The chosen docked structures of the designed vaccine-TLR2/4 were subjected to simulations of MD to calculate the level of their structural stability. On the basis of the GROMOS96 54A7 force field, we conducted a separate 50 ns MD simulation for each docked complex.

#### Investigation of stability

**Fig. 9:**
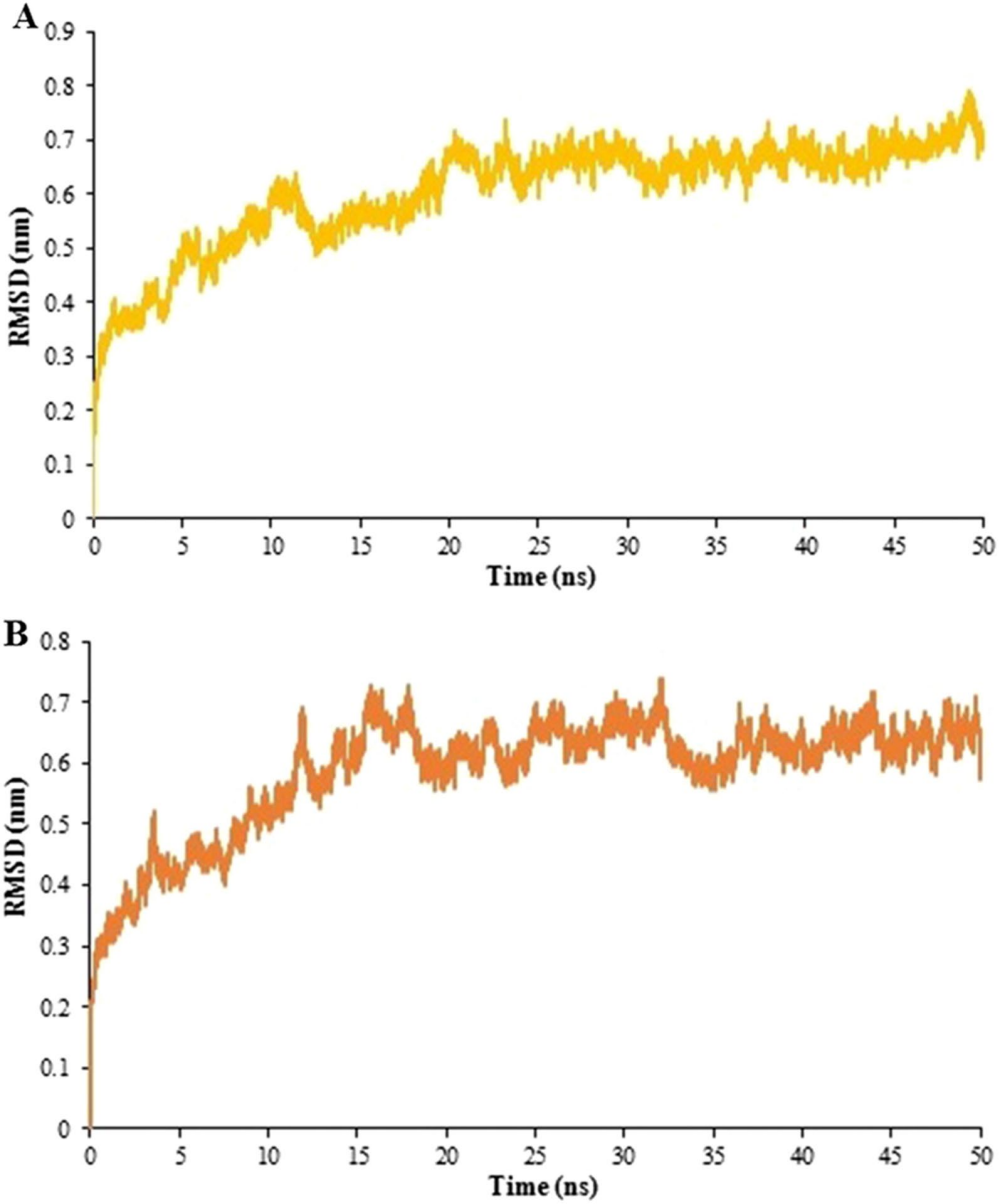
RMSD plots for 50 ns simulation runs for vaccine-receptor complexes. The changes in the RMSD values were in the range of 0.6–0.8 nm after 20 ns for (**A**) ODAC-TLR2 and 0.57–0.72 nm after 15 ns for (**B**) ODAC-TLR4. The results showed that the binding of the vaccine to both receptor is stable

The RMS Deviation was applied for evaluating the vaccine-TLR2/-TLR4 complex stability. The backbone deviations of chosen complexes of the initial conformations were plotted as a function of time. The ODAC-TLR2 and ODAC-TLR4 showed RMSD values in the range of 0.6–0.8 nm after 20 ns and 0.57–0.72 nm after 15 ns, respectively, as they are displayed in Fig. 9.

#### Analysis of flexibility

**Fig. 10:**
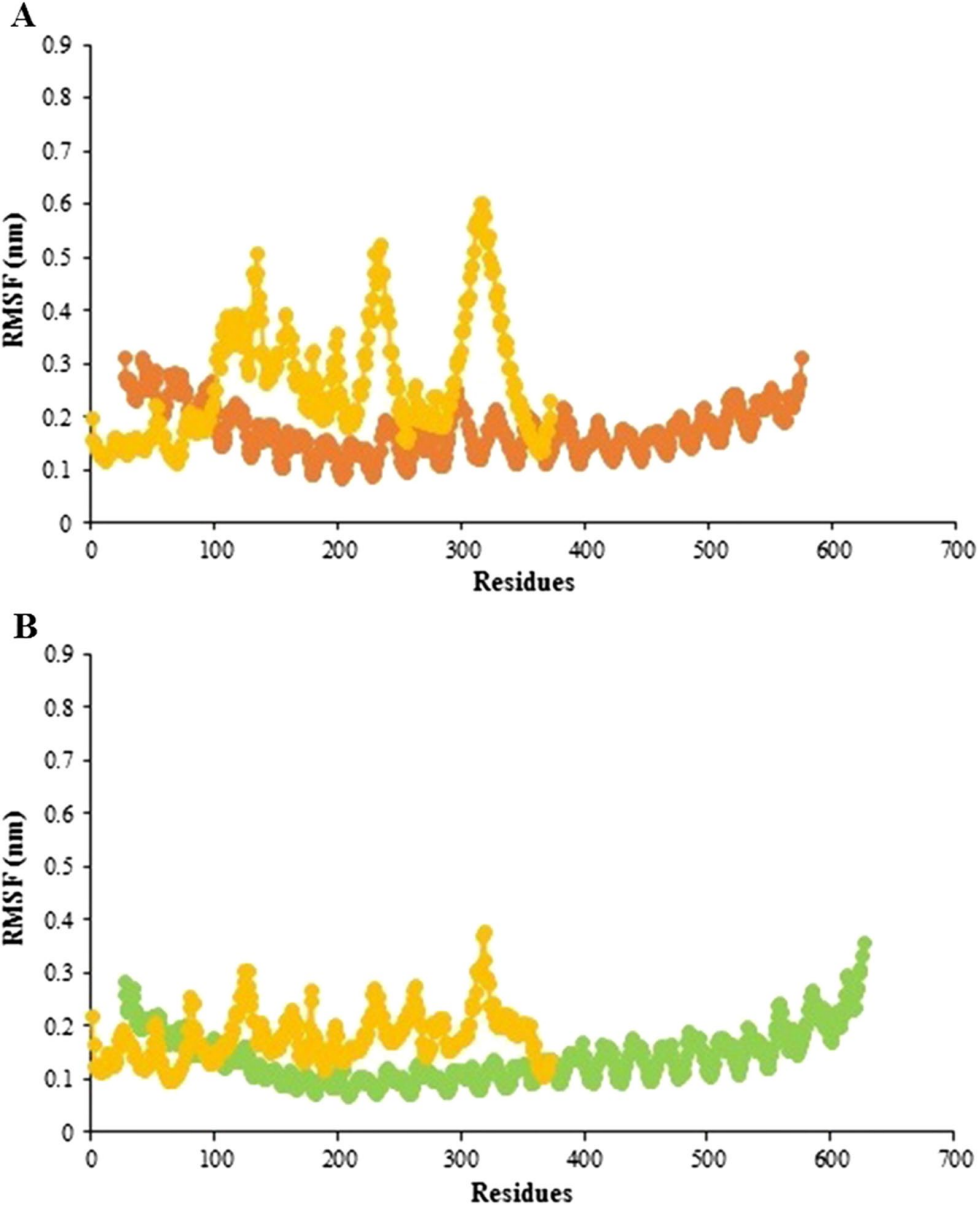
RMS fluctuation values as a function of residue number for the vaccine-receptor complexes. **A** RMSF plots for the vaccine and TLR2 are depicted in yellow and orange, respectively. The plots reveal that the vaccine residues except for 112 residues of PepO have a greater degree of flexibility than that of TLR2. **B** RMSF plots for the vaccine and TLR4 are represented in yellow and green, respectively. The ODAC and TLR4 residues show no significant flexibility

The RMS fluctuation is another way to anticipate the dynamic stability (DS) of the docked complex that evaluates fluctuations of the residues. The RMSF values showed that PepO residues in ODAC had almost the same degree of flexibility as TLR2 amino acids, while the rest of ODAC residues had a greater degree of flexibility than TLR2 residues (Fig. 10A). Likewise, as shown in Fig. 10B, the RMSF value of residues in ODAC and TLR4 revealed no notable flexibility.

### The simulation of the immune system

**Fig. 11:**
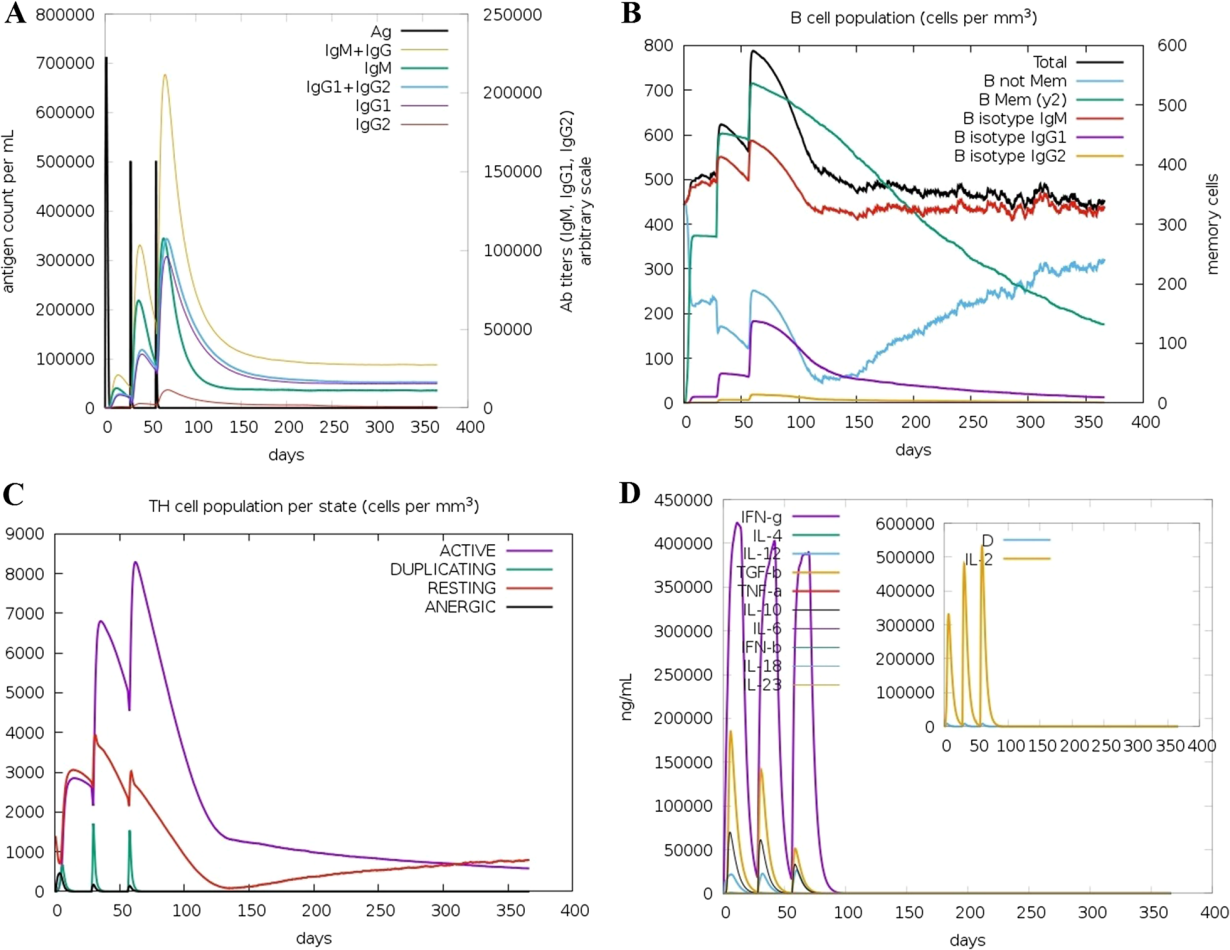
C-ImmSim plots display the immune profile of the candidate vaccine. **A** The production of immunoglobulins and their subclasses in response to 3 injections of antigen; Ag and Ig subtypes/subclasses are depicted as black vertical lines and colored peaks, respectively. **B** The development of the B-cell population following 3 injections. **C** The evolution of CD4 T-helper cells. **D** Cytokine and interleukin levels after three injections

The outcomes of the immune simulations provided by the C-ImmSim online server were consistent with strong immune reactions as shown in Fig. 11. It was found that the developed vaccine construct could able to elicit the primary response and powerful secondary and tertiary responses. The primary response is illustrated by the raised levels of IgM antibodies after a 5–7-day delay of antigen exposure. The secondary and tertiary responses are characterized by the increase in the population of B-cells and expression of immunoglobulins (IgM, IgG1 + IgG2, and IgG + IgM), resulting in a subsequent decline in the concentration of antigen (Fig. 11 A, B). The suggested vaccine, in addition to inducing high proliferation of B cells, also leads to memory B-cell generation. Likewise, the outcomes demonstrated that the construct could increase the population of T helper cells with memory development (Fig. 11 C). As depicted in Fig. 11 there is also an increase in the IFN-γ concentration following each injection. These results clearly display that the designed vaccine could efficiently elicit immune responses and provide the basis for immunity against pneumococcal infection.

### Reverse translation, codon adaptation, and in silico cloning

**Fig. 12:**
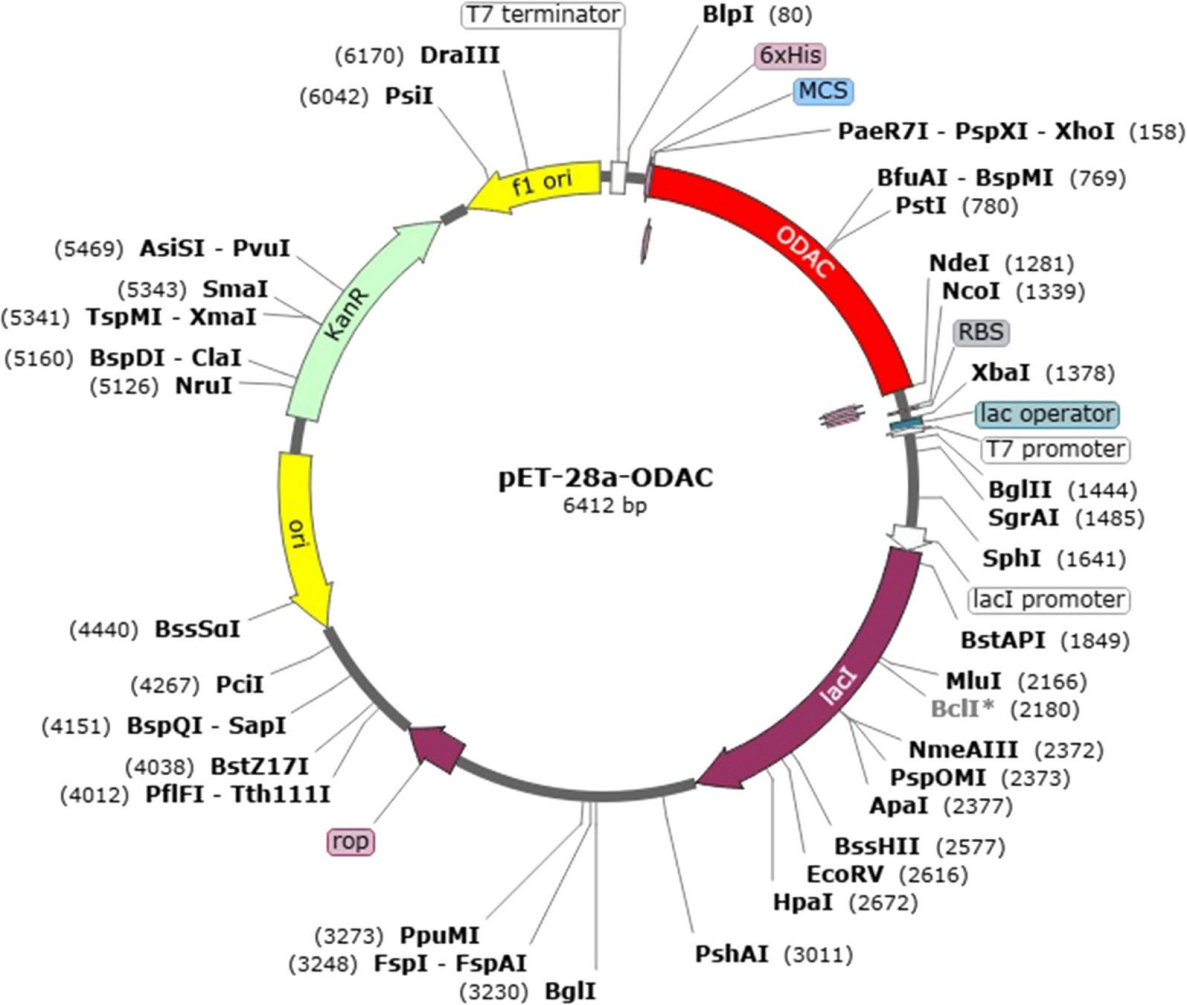
In silico cloning of epitope vaccine sequence within the pET28a ( +) vector through SnapGene software free-trial. The segment painted in red is the nucleotide sequence of ODAC vaccine and the other parts of the circle show the backbone of pET28a( +)

JCat tool was employed for reverse translation and codon adaptation of the devised vaccine candidate in order to maximal expression of it in *E. coli* K-12. The vaccine sequence after the reverse translation was 1116 nucleotides long. According to the codon optimization findings, the CAI value and CG content were 0.99 and 46.41%, respectively, which are accepted since they are within the permitted limit. These outcomes confirm a proper expression of the engineered vaccine in *E. coli* K-12. Ultimately, the ODAC vaccine sequence was successfully cloned into the pET-28a( +) between *Nde*I and *Xho*I enzymes by SnapGene software free-trial (Fig. 12).

## Discussion

*Streptococcus pneumoniae* is a powerful pathogen owing to its ability to adhere to cells, invasion to host tissues, and escape the immune system of the host [82], and can cause fatal diseases such as sepsis, pneumonia, and meningitis with a significant death rate worldwide [83]. The currently existing pneumococcal vaccines have several limitations including serotype dependence [7, 84] and so they are not capable of protecting subjects against all Pnc serotypes. A hopeful alternative can be the utilization of proteins and peptide fragments, some of which have been investigated as vaccine candidates in pre-clinical trials, either alone or in combination with each other. Pneumococcal proteins commonly expressed across all serotypes have been regarded as a potential candidate for developing serotype-independent Pnc vaccines [7]. In many biological subjects, in silico techniques could be highly useful to decrease the cost and time of experimental researches [37, 85, 86]. Epitope identification by immunoinformatics tools could be quite helpful in the different applications within the field of epitope mapping, such as designing peptide-based vaccines, identifying immunological processes, predicting epitopes used in the disease diagnosis, and so on [87–90]. Peptide-based vaccines are one of the alternative and appealing approaches to develop vaccines, which only include peptide fragments with the highest antigenicity and the most ability to induce immune responses [91, 92]. In reality, the immune responses are only formed against immunogenic epitopes, and other parts of the protein virulence factor causing unwanted reactions can be eliminated [93]. The combining of several highly-conserved peptides can be employed to induce both native and acquired immune responses, and prevent various stages of Pnc infection, as well as present broader serotype coverage [7].

PspC protein which is almost found in all pneumococcal strains is one of the major and surface-exposed virulence factors [21]. A study showed that the survival time for mice vaccinated with a combination of PspC and a genetic toxoid derivative of pneumolysin (PdB) was considerably longer than that for mice treated only with PdB, but was not remarkably different from that for mice immunized only with PspC [94]. In another study in 2014, the PspC peptides which participate in adherence and invasion were genetically fused to L460D pneumolysoid protein. The fusion construct was more immunogenic than L460D pneumolysoid alone, and anti-fusion antibodies were active against pneumococcus. Immunization of mice with the fusion construct protected them from the Pnc carriage, pneumonia, meningitis, bacteremia, and sepsis [95]. PhtD and PhtE antigens, two members of the conserved Pht family, have been found to be surface-exposed and expressed almost in all pneumococcal strains [96]. It has been exhibited that both antigens induce efficient protection against colonization, pneumonia and sepsis in animal models [97]. Godfroid et al. indicated that mice vaccinated with this family were protected more effective against pneumonia compared to those immunized with other Pnc vaccine candidates such as PsaA and PspA [96]. In the research of Verhoeven et al., the efficacy of a multivalent vaccine containing PhtD, choline-binding protein A (PcpA), and detoxified pneumolysin (PlyD1) was evaluated and it was shown that the vaccine protected the infant mouse model against serotypes 3 and 6A pneumococcus [98]. Plumptre et al., demonstrated the truncated derivatives of PhtD as vaccine antigens in animal models were more effective than the whole PhtD protein [12]. Pneumococcal surface adhesion A is another conserved and immunogenic antigen that is expressed by all Pnc strains [99]. Lin et al. reported that mice vaccinated with the 23-valent capsular polysaccharide (CPS)–rPsaA conjugate showed rapid pneumococcal clearance from blood and could provide the best protection against Pnc challenge [100]. Lu et al., prepared a fusion conjugate consisting of a fusion protein of pneumolysin nontoxic derivative (PdT) with PsaA coupled to cell wall polysaccharide (CWPS). The fusion conjugate protected mice against colonization, while mice vaccinated with the mixture of the three antigens were not protected [101]. In the study of Lu et al., in 2015, the fusion protein PspA-PsaA was assessed in an animal model against fatal challenges with pneumococci. The results showed that levels of antibodies against both proteins increased in mice vaccinated by the fusion protein. Also following fatal challenge, immunized mice revealed decreased levels of pneumococci in the lungs and blood compared with the control group and were protected against pneumococcal challenge [32]. Another study showed that PsaA antigen prevented pneumococcal carriage more than PspA [102].

The principal drawback of epitope vaccines is that they could be degraded quickly by proteinase in the body, making them hard to be recognized by immune cell receptors [103, 104]. One of the approaches recommended for enhancing the immune responses elicited by epitope vaccines is to utilize adjuvants in the vaccine constructs [104]. In recent decades, TLR agonists have been broadly examined as candidate adjuvants for vaccine constructs [38]. There are various TLR agonists with different sources which a group of them are bacterial proteins capable of inducing TLR signaling and native immune responses [39]. Pneumococcal endopeptidase O is one of the bacterial TLR agonists that can be considered as a potential candidate adjuvant [39]. Shu and colleagues identified the Pnc endopeptidase O to be a TLR2/TLR4 bi-ligand. To distinguish the binding site of TLRs in PepO, they prepared two recombinant truncated forms of it, PepO 1–430 and PepO 431–630, according to two conserved domains of PepO named M13_N domain and M13 domain, respectively. Their result revealed that TLR2 and 4 bind to the M13_N domain of this protein [41]. In this regard, in the present study, to identify the main residues potentially involved in the interaction between the N-PepO and TLRs, we employed molecular docking using ClusPro 2.0. Following docking, the best complexes were chosen with the lowest energy docking mode -859.4 (for N-PepO-TLR2) and -967.1 (for N-PepO-TLR4). The DIMPLOT from LigPlot + v2.2.4 was used to analyze the chosen complexes (see Figs. 2 and 3). The N-PepO residues Asp^34^, Arg^3^, Gln^5^, Asp^6^, Phe^8^, Tyr^9^, Asn^13^, Glu^15^, Arg^354^, and Lys^357^, and likewise, the N-PepO amino acids Tyr^4^, Gln^5^, Ala^11^, Ile^12^, Asn^13^, Glu^15^, Thr^19^, and Glu^21^ were predicted to play roles in the interactions with TLR2 and TLR4, respectively (see Table 1). Ultimately, to maintain the domain structure, a truncated fragment from N-PepO (residues 1–112) was selected as a potential TLR2/4 agonist candidate in this research.

The strategies applied in this investigation were the use of derivatives of three important virulence proteins PspC, PhtD, and PsaA into the vaccine construct which could help at preventing infection by pneumococci, and the identification of epitope-rich domains that could provoke both robust B-and T-cell immune responses. An efficient epitope vaccine should be engineered to include B and T cell epitopes capable of providing effective reactions to a specific infection [105]. Since the recognition of putative B and T cell epitopes using the wet-lab approach requires experimental screening of a large number of active epitopes against inactive epitopes, the computational approach can act as a cost-effective, rapid, reliable, and accurate alternative [106, 107]. In this regard, the use of a consensus prediction strategy is more reliable and robust providing better results than any of the individual prediction methods [108]. Hence in this study, the B- and helper T-cell epitopes were predicted through various reliable databases and servers including ABCpred, LBtope, IEDB, Ellipro, DiscoTope, and NetMHCIIpan servers to achieve more reliable results. Finally, three epitope-rich regions containing high-ranked and shared epitopes, i.e. residues 196–256, 100–187, and 276–363 from PhtD-C, PsaA, and PspC, respectively, were chosen (Table 2). According to IEBD database, the selected region of PspC was consistent with two significant epitopes EDRRNYPSNT and EAKEPRNEEKVKQAK experimentally reported as B cell epitopes by Vadesilho et al. The outcomes of recent experimental study could support our predictions. The physical–chemical features and 3-D structure of the epitope-based vaccines correlate with the nature of the epitopes, linkers, and adjuvants, as well as their place and order in the vaccine construct [109]. Herein, the chosen regions of PhtD, PsaA, and PspC were joined together in an appropriate pattern using suitable linkers. In a similar manner to Tian et al., the GGSSGG flexible linkers were applied to keep the independent folding of domains and their immunological activities [63]. In addition, the EAAAK helical linker was employed for connecting the truncated N-PepO, as an adjuvant candidate, to the N-terminal of the above-quoted sequence to boost the stability and immunogenicity of the construct [64, 65]. None of the selected three domains (196–256 of PhtD-C, 100–187 of PsaA, and 276–363 of PspC) has been used before and also the truncated fragment of PepO (residues 1 to 112) was not introduced earlier as a TLR agonist adjuvant candidate, showing the novelty of our study. The final engineered vaccine construct (ODAC) was assessed for toxicity, antigenicity, and allergenicity. It was antigenic, non-toxic, and non-allergic, which indicated its effectiveness in evoking sturdy immune responses without no harmful reactions. According to SOLpro results, the solubility of vaccine upon overexpression in *E. coli* host, which is one of the fundamental requirements of most functional and biochemical investigations, was 0.893 [110, 111]. The theoretical pI of the ODAC vaccine was 5.29 which indicates the ODAC is basic in nature. The molecular weight of the construct was 41.93 kDa, which is ideal because the purification of protein constructs with Mws fewer than 110 kDa is faster and easier [104, 112]. The half-life (T1/2) of the engineered vaccine was computed to be 30 h, > 20 h, and more than 10 h, in mammalian reticulocytes, yeast, and *E. coli*, respectively. The half-life is the time which takes for 50% of the protein amount inside a cell to eliminate following its synthesis in the cell. ProtParam uses the "N-end rule" which links the protein's half-life to the identity of its amino-terminal residues that applies to both prokaryotic and eukaryotic organisms [113]. The aliphatic index of the designed vaccine was computed to be 68.01, implying that it could be a thermostable vaccine [114]. The GRAVY index of ODAC vaccine was -0.954, and the negative value of this parameter demonstrates that the construct is hydrophilic and could well interact with molecules of water [115]. The instability index is assessed to estimate whether a vaccine is (un)stable, if this index of a protein is below (above) 40 then it’s called stable (unstable) [72, 115]. The instability index of the ODAC construct was computed to be 36.83, and as the value is < 40, the engineered vaccine is deemed stable (see Table 3).

The initial tertiary structure of the engineered vaccine was modeled via the Robetta server. Following this step, the refining process was utilized to promote the quality of the 3D structure, bringing it closer to the native structure. The qualities of the unrefined and refined models were compared through the R-plot, ProSA Z-score, and ERRAT score. The results showed that all scores related to the structure of the refined model improved well (Fig. 5). The refined 3D structure was subjected to discontinuous B-cell epitopes identification and molecular docking process. A proper vaccine ought to include B-cell epitopes, besides to T-cell epitopes, with the purpose of ensuring the induction of humoral immunity [116]. The conformational B-cell epitopes were predicted on the basis of the interaction of vaccine-antibody through the Ellipro server, and the epitopes with a score of > 0.5 were picked (see Table 4). The refined model had a plurality of discontinuous B-cell epitopes, demonstrating that the engineered vaccine is quite potent in evoking humoral immunity. The docking processes between the ODAC vaccine and TLRs 2 and 4 were performed by Cluspro 2.0. The best complex models were considered with the lowest energy docking mode -922.1 and -798.3 for ODAC-TLR2 and -TLR4, respectively. The DIMPLOT was utilized for analyzing the chosen models (Figs. 7 and 8). The ODAC construct residues Met^1^, Thr^2^, Asp^6^, Tyr^9^, Asp^10^, Asn^13^Asp^34^, Asp^36^, Gln^37^, Glu^40^, Trp^50^, and Glu^58^, and likewise, the ODAC vaccine amino acids Thr^2^, Arg^3^, Asp^10^, Glu^15^, Trp^16^, Asp^59^, Arg^70^, His^368^, His^368^, His^369^, and His^372^ were predicted to perform roles in the interactions with TLR2 and TLR4, respectively (see Table 5). The outcomes of the molecular docking showed that the truncated N-PepO of designed vaccine interacted favorably with TLRs, indicating it could act as a possible adjuvant for TLR2/TLR4 activation. With the purpose of assessing the molecular stability of ODAC-TLR2/4 docked complexes, simulations of MD were conducted through GROMACS. The structural fluctuations of the ODAC-TLR2 and ODAC-TLR4 reached the range of 0.6–0.8 nm and 0.57–0.72 nm after 20 and 15 ns, respectively (Fig. 9). The outcomes revealed that the binding of the designed vaccine to the TLR receptors was stable during simulation times. The truncated region of N-PepO as the TLR agonist was capable to keep well these connections. RMS Fluctuation is the other tool for computing the dynamic stability of complexes. The findings of RMSF showed that the residues of ODAC, except the truncated N-PepO residues, were more flexible than those of TLR2, while the engineered vaccine and TLR4 amino acids showed no notable flexibility (Fig. 10). Conforming to the RMSF analyses, the truncated N-PepO had the lowest fluctuations in the areas having the most interactions with the TLR2/4 receptors. The results from the C-ImmSim server were consistent with actual immune responses previously reported in pneumococcal infections [117]. The Induction of B and T cell memory responses is deemed as one of the criteria for being an effective candidate vaccine [118]. It was found that populations of memory B and T cells were increased by each injection of ODAC and these levels were maintained after the third injection (Fig. 11), indicating our construct as a suitable one. Another observation of interest was that the level of IFN-γ was elevated following the first injection and remained at the peaks after repeated the injections. This shows a high concentration of T helper cells and thus the efficient production of immunoglobulin, which supports humoral responses [119]. To enhance the expression of the designed vaccine in *E. coli* K-12, codon adaptation was conducted through JCat tool. The ODAC construct possessed the CAI value of 0.99 and GC content of 46.41%. Since CAI values > 0.8 and CG content > 30 and < 70% are considered to be desirable for expression [104, 115], our outcomes are satisfactory.

In numerous recent studies, the reliable computational procedures have been used to select efficient epitopes and design novel vaccines against various pathogens such as *Mycobacterium tuberculosis* [118], *Helicobacter Pylori* [42], *Onchocerca volvulus* [111], *Plasmodium* [120], and Crimean-Congo hemorrhagic fever virus [119]. In this respect, the present study recommends a final peptide construct as the best epitope vaccine based on the strategies used in the research to be an attractive intervention against the *Streptococcus pneumoniae* pathogen. Furthermore, antigenic epitope-rich portions from each of the proteins involved in the construct could also be employed in future studies to develop the other new subunit vaccines consisting of other virulence proteins. In addition, the new truncated domain of PepO could play as a new adjuvant candidate in other studies to increase the potency of vaccines against pneumococci or other pathogens.

## Conclusions

Current pneumococcal vaccines, although, have been useful in decreasing the rate of invasive Pnc diseases, they have some issues. The main goal of this research was designing a novel epitope-based vaccine that could cover the limitations of existing Pnc vaccines. In this regard, the immunodominant epitope-rich regions from highly protective Pnc antigens, namely PhtD, PsaA, PspC, were predicted by immunoinformatics tools and joined together using proper linkers. Then, the truncated fragment of N-PepO (as the potential adjuvant) was linked to the engineered construct in order to enhance the vaccine's immunogenicity. The final designed construct was determined to be antigenic and non-allergenic, and its physical–chemical characteristics were satisfactory. Molecular docking was conducted for evaluating the binding affinity of the vaccine to TLR2/4 receptors. Simulations of MD were applied to confirm the stability of the considered docked complexes. Finally, the expression efficiency of the epitope-based vaccine was assessed. Although the outcomes of this research were quite remarkable, the potency of the engineered vaccine ought to be confirmed in laboratory and animal models.

## Supplementary Information

**Additional file 1: Fig. S1.** TMHMM result for PhtD-C (A), PsaA (B), and PspC (C). The TMHMM plots (A, B, and C) show no existence of transmembrane helices.** Fig. S2**. SignalP 4.0 web-server output for the sequences of PsaA and PspC. The C-, S-, and Y-scores represent the predicted cleavage site value, the projected signal peptide value, and a combination of C-and S-scores, respectively. (A) The anticipated cleavage site of PsaA is located between positions 19 and 20. (B) In PspC, there is not any cleavage site. **Fig. S3.** Three-dimensional structures of proteins PhtD-C (A) and PspC (B). The structures were predicted by I-TASSER. **Fig. S4.** Validation of the refined tertiary structures. A and B show the results of ProSA web for PhtD-C and PspC, respectively. The a and b display Ramachandran plots of PhtD-C and PspC, respectively. **Fig. S5.** Three-dimensional structure of TLR agonist N-PepO. The structure of N-PepO was predicted and refined by I-TASSER and GalaxyRefine, respectively. **Table S1**. The results of validation before and after refinement. Ramachandran plot statistics from PROCHECK and Z-score from ProSA web server. **Table S2.** Prediction of linear B-cell epitopes from PhtD-C by LBTope, ABCpred, IEDB Emini tool, and Ellipro. **Table S3.** Prediction of linear B-cell epitopes from PsaA by LBTope, ABCpred, Emini, and Ellipro. **Table S4.** Prediction of linear B-cell epitopes from PspC by LBTope, ABCpred, Emini, and Ellipro. **Table S5.** Prediction of conformational epitopes from PhtD-C via Ellipro and Discotope. **Table S6.** Prediction of conformational epitopes from PsaA via Ellipro and Discotope. **Table S7.** Prediction of conformational epitopes from PspC via Ellipro and Discotope. **Table S8.** Prediction of MHCII epitopes from PhtD-C by IEDB and NetMHCIIpan. **Table S9.** Prediction of MHCII epitopes from PsaA by IEDB and NetMHCIIpan. **Table S10.** Prediction of MHCII epitopes from PspC by IEDB and NetMHCIIpan.

## Data Availability

Not applicable.

## Abbreviations

Pnc: Pneumococcal
PPV: Pneumococcal polysaccharide vaccines
PCV: Protein-conjugated polysaccharide vaccines
PspA: Pneumococcal surface protein A
PspC: Pneumococcal surface protein C
Pht: Pneumococcal histidine triad
ABC: ATP-binding cassette
Hic: H-binding inhibitor of complement
PbcA: C3-binding protein A
SpsA: *S. pneumoniae*-Secretory IgA-binding protein
CbpA: Choline-binding protein A
N-ter: N-terminal
C-ter: C-terminal
Zn2^+^: Zinc
Mn: Manganese
TLR: Toll-like receptor
APCs: Antigen presenting cells
PepO: *S. pneumoniae* Endopeptidase O
PDB: Protein data bank
C-score: Confidence score
TM: Template modeling
RMSD: Root mean square deviation
DS: Discovery studio
His6: Hexa-histidine
JCat: Java codon adaptation tool
CAI: Codon adaptation index
pI: Isoelectric point
Mw: Molecular weight
GRAVY: Grand average of hydropathicity
+ R: Total number of positive residues
− R: Total number of negative residues
PcpA: Choline-binding protein A
PlyD1: Detoxified pneumolysin
CWPS: Cell wall polysaccharide
PhtA: Pneumococcal histidine triad A
PhtB: Pneumococcal histidine triad B
PhtD: Pneumococcal histidine triad D
PhtE: Pneumococcal histidine triad E
PspC3: PspC from group 3
PhtD-C: C-terminal of PhtD
PsaA: Pneumococcal surface adhesion A
RMSF: Root Mean Square Fluctuation
RE: Restriction Enzymes
ODAC: The final designed vaccine in this study
AI: Aliphatic index;
PdB: Toxoid derivative of pneumolysin
CPS–rPsaA: 23-valent capsular polysaccharide–rPsaA conjugate
PdT: Pneumolysin nontoxic derivative
