# Supplementary material for: In silico designing of a novel epitope-based candidate vaccine against Streptococcus pneumoniae with introduction of a new domain of PepO as adjuvant
Source: J Transl Med. 2022 Sep 4;20:389. doi: 10.1186/s12967-022-03590-6 (PMC9440865; doi:10.1186/s12967-022-03590-6)
Supplement: Supplementary file 1 — Additional file 1: Fig. S1. TMHMM result for PhtD-C (A), PsaA (B), and PspC (C). The TMHMM plots (A, B, and C) show no existence of transmembrane helices. Fig. S2. SignalP 4.0 web-server output for the sequences of PsaA and PspC. The C-, S-, and Y-scores represent the predicted cleavage site value, the projected signal peptide value, and a combination of C-and S-scores, respectively. (A) The anticipated cleavage site of PsaA is located between positions 19 and 20. (B) In PspC, there is not any cleavage site. Fig. S3. Three-dimensional structures of proteins PhtD-C (A) and PspC (B). The structures were predicted by I-TASSER. Fig. S4. Validation of the refined tertiary structures. A and B show the results of ProSA web for PhtD-C and PspC, respectively. The a and b display Ramachandran plots of PhtD-C and PspC, respectively. Fig. S5. Three-dimensional structure of TLR agonist N-PepO. The structure of N-PepO was predicted and refined by I-TASSER and GalaxyRefine, respectively. Table S1. The results of validation before and after refinement. Ramachandran plot statistics from PROCHECK and Z-score from ProSA web server. Table S2. Prediction of linear B-cell epitopes from PhtD-C by LBTope, ABCpred, IEDB Emini tool, and Ellipro. Table S3. Prediction of linear B-cell epitopes from PsaA by LBTope, ABCpred, Emini, and Ellipro. Table S4. Prediction of linear B-cell epitopes from PspC by LBTope, ABCpred, Emini, and Ellipro. Table S5. Prediction of conformational epitopes from PhtD-C via Ellipro and Discotope. Table S6. Prediction of conformational epitopes from PsaA via Ellipro and Discotope. Table S7. Prediction of conformational epitopes from PspC via Ellipro and Discotope. Table S8. Prediction of MHCII epitopes from PhtD-C by IEDB and NetMHCIIpan. Table S9. Prediction of MHCII epitopes from PsaA by IEDB and NetMHCIIpan. Table S10. Prediction of MHCII epitopes from PspC by IEDB and NetMHCIIpan. [file 12967_2022_3590_MOESM1_ESM.docx]

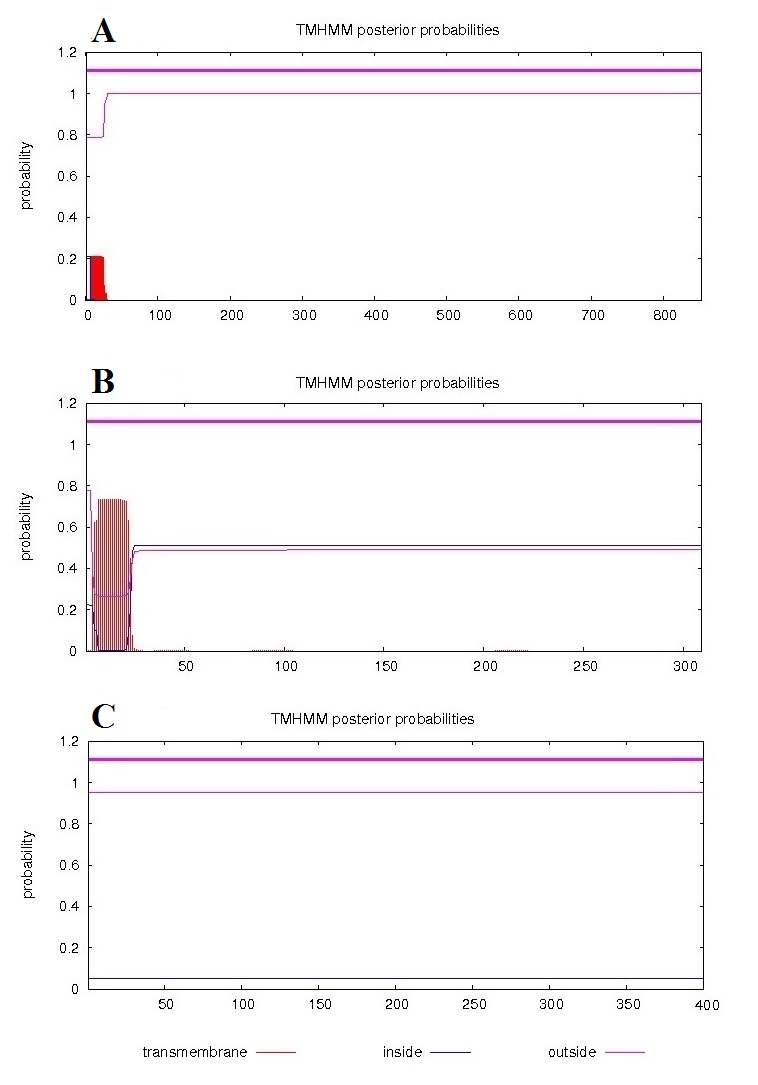


**Supplementary Figure 1.** TMHMM result for PhtD-C (A), PsaA (B), and PspC (C). The TMHMM plots (A, B, and C) show no existence of transmembrane helices.


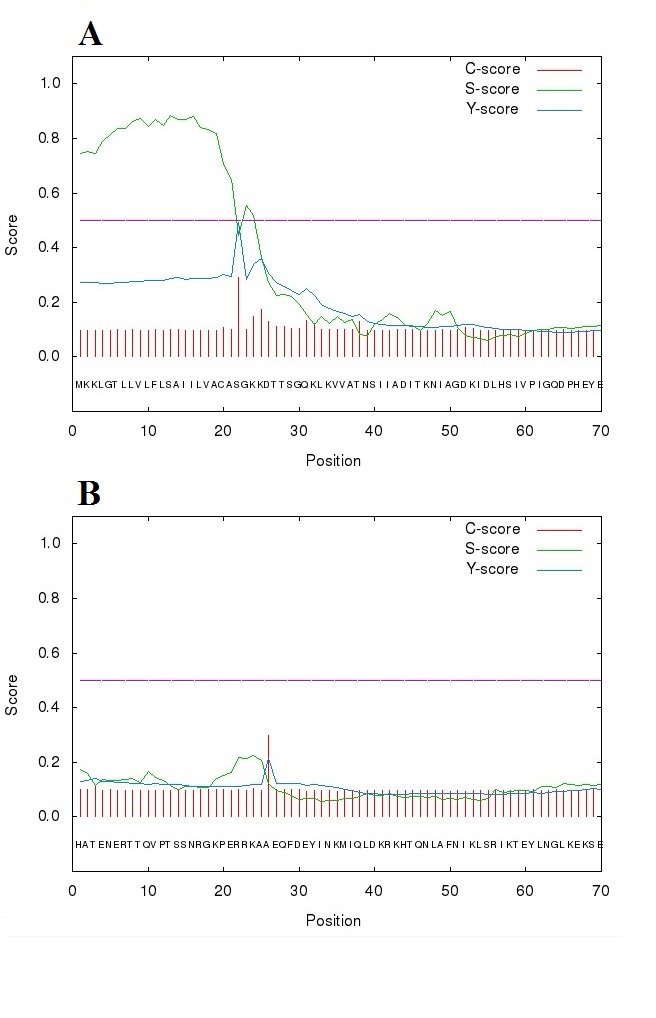


**Supplementary Figure 2**. SignalP 4.0 web-server output for the sequences of PsaA and PspC. The C-, S-, and Y-scores represent the predicted cleavage site value, the projected signal peptide value, and a combination of C-and S-scores, respectively. (A) The anticipated cleavage site of PsaA is located between positions 19 and 20. (B) In PspC, there is not any cleavage site.


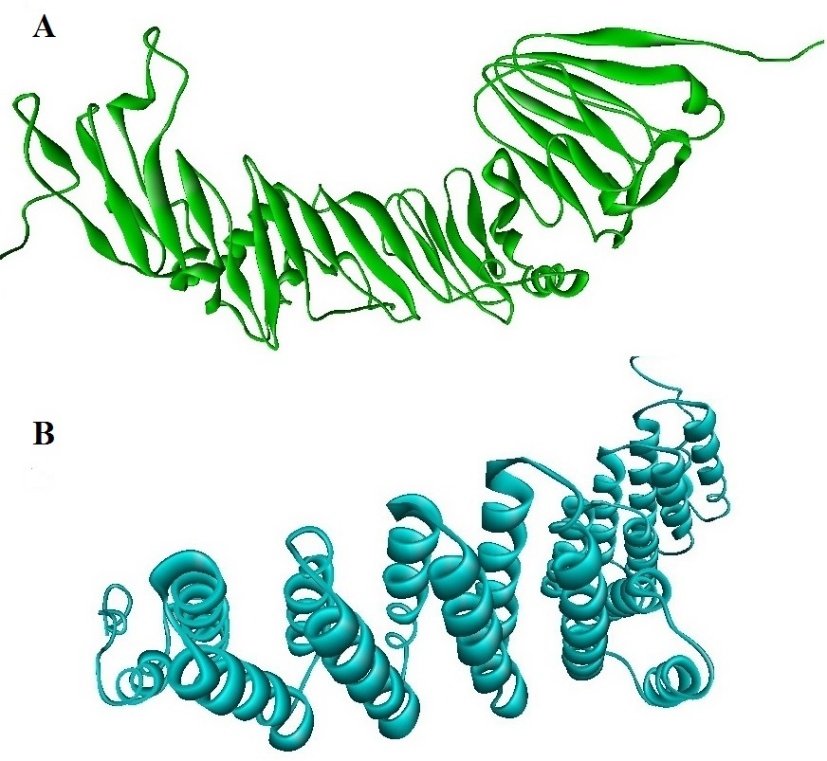


**Supplementary Figure 3**. Three-dimensional structures of proteins PhtD-C (A) and PspC (B). The structures were predicted by I-TASSER.


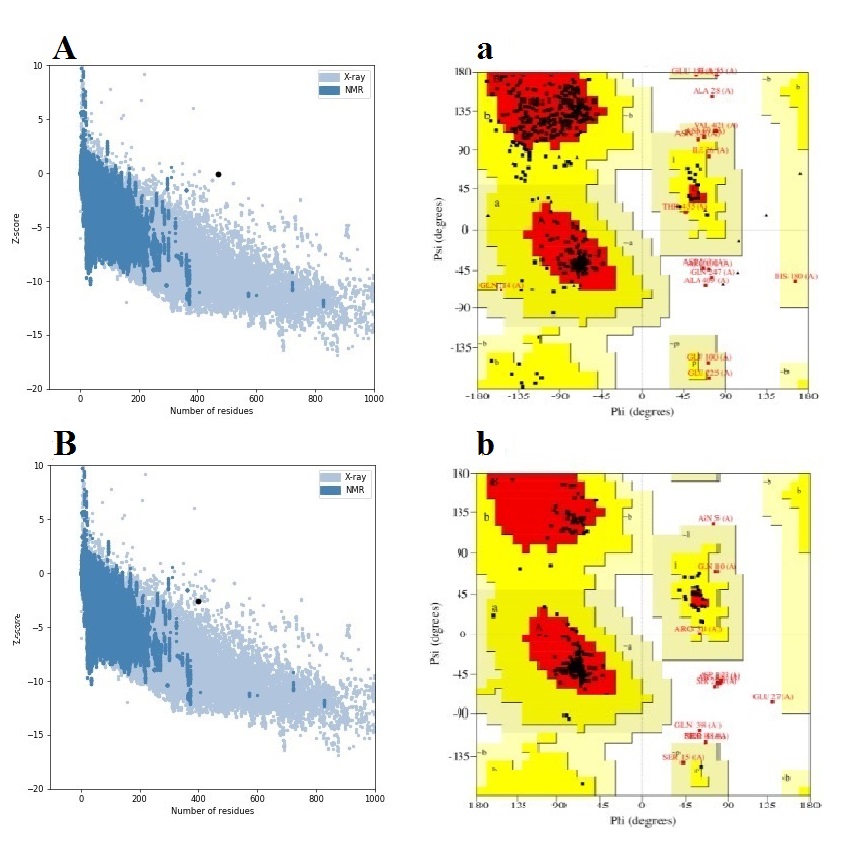


**Supplementary Figure 4**. Validation of the refined tertiary structures. A and B show the results of ProSA web for PhtD-C and PspC, respectively. The a and b display Ramachandran plots of PhtD-C and PspC, respectively.


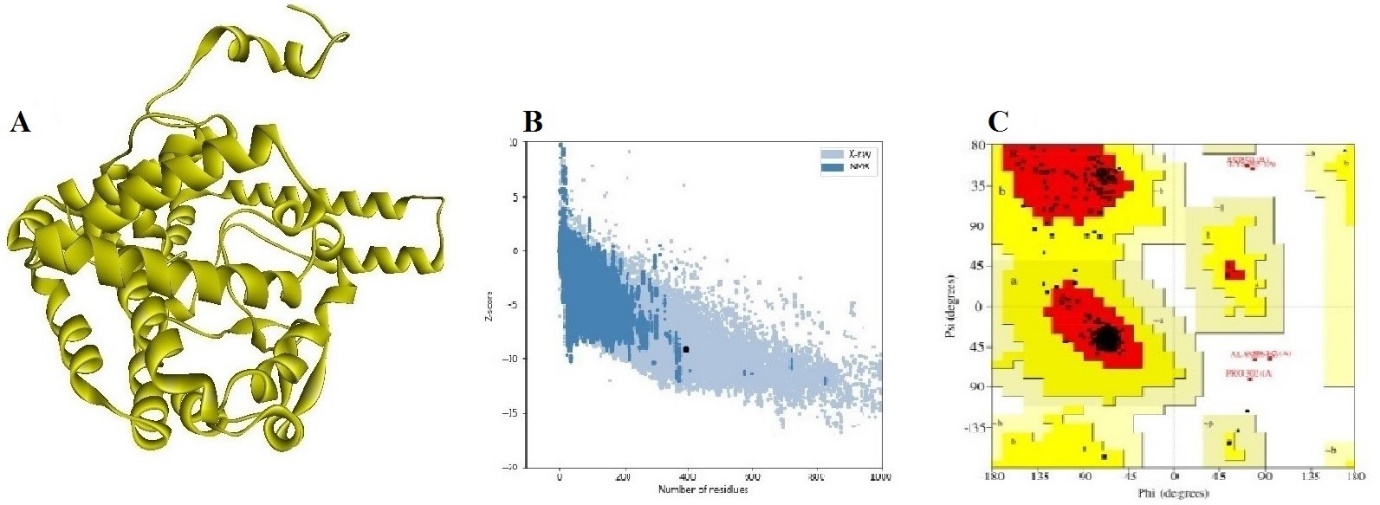


**Supplementary Figure 5.** Three-dimensional structure of TLR agonist N-PepO. The structure of N-PepO was predicted and refined by I-TASSER and GalaxyRefine, respectively.

**Supplementary Table 1**. The results of validation before and after refinement. Ramachandran plot statistics from PROCHECK and Z-score from ProSA web server.

| **Proteins** | **Before Refinement** | | | | **After Refinement** | | | |
| --- | --- | --- | --- | --- | --- | --- | --- | --- |
|  | **Z-score** | **Ramachandran plot** | | | **Z-score** | **Ramachandran plot** | | |
|  |  | **Most favored zones (%)** | **Allowed zones (%)** | **Disallowed zones (%)** |  | **Most favored zones (%)** | **Allowed zones (%)** | **Disallowed zones (%)** |
| **PhtD-C** | 0.47 | 61.3 | 35.8 | 2.9 | -0.04 | 81.7 | 16.9 | 1.4 |
| **PspC** | -2.17 | 80.5 | 18.4 | 1.1 | -2.62 | 91.5 | 6.6 | 1.9 |
| **N-PepO** | -8.72 | 87.1 | 11.8 | 1.1 | -9.1 | 94.4 | 4.5 | 1.1 |

**Supplementary Table 2.** Prediction of linear B-cell epitopes from PhtD-C by LBTope, ABCpred, IEDB Emini tool, and Ellipro.

| LBTope | | | | | | | |
| --- | --- | --- | --- | --- | --- | --- | --- |
| Sequence | **% Probability of correct prediction** | **Sequence** | **% Probability of correct prediction** | **Sequence** | **% Probability of correct prediction** | **Sequence** | **% Probability of correct prediction** |
| KVGDGYVFEENGVPR | 62.17 | APIRHPERLGKPNAQ | 62.70 | FEWFDEGLYEAPKGY | 64.84 | EDKEHDEVSEPTHPE | 60.76 |
| VGDGYVFEENGVPRY | 62.30 | PIRHPERLGKPNAQI | 60.66 | EWFDEGLYEAPKGYS | 65.38 | DKEHDEVSEPTHPES | 63.36 |
| GDGYVFEENGVPRYI | 68.29 | IFDPRDITSDEGDAY | 61.34 | GLYEAPKGYSLEDLL | 62.65 | KEHDEVSEPTHPESD | 72.74 |
| DGYVFEENGVPRYIP | 74.69 | FDPRDITSDEGDAYV | 65.07 | LYEAPKGYSLEDLLA | 71.19 | EHDEVSEPTHPESDE | 56.97 |
| GYVFEENGVPRYIPA | 62.80 | WIKKDSLSEAERAAA | 66.66 | YEAPKGYSLEDLLAT | 61.84 | HDEVSEPTHPESDEK | 58.14 |
| PRYIPAKDLSAETAA | 61.01 | IKKDSLSEAERAAAQ | 69.18 | EAPKGYSLEDLLATV | 60.18 | DEVSEPTHPESDEKE | 65.29 |
| TAAGIDSKLAKQESL | 61.55 | AYAKEKGLTPPSTDH | 60.71 | PKGYSLEDLLATVKY | 62.42 | EVSEPTHPESDEKEN | 68.67 |
| AAGIDSKLAKQESLS | 61.43 | KGAEAIYNRVKAAKK | 60.45 | GYSLEDLLATVKYYV | 64.28 | VSEPTHPESDEKENH | 66.09 |
| AGIDSKLAKQESLSH | 62.01 | AEAIYNRVKAAKKVP | 63.69 | YSLEDLLATVKYYVE | 70.99 | SEPTHPESDEKENHV | 64.58 |
| GIDSKLAKQESLSHK | 67.50 | EAIYNRVKAAKKVPL | 67.67 | SLEDLLATVKYYVEH | 71.99 | EPTHPESDEKENHVG | 78.08 |
| IDSKLAKQESLSHKL | 65.33 | AIYNRVKAAKKVPLD | 64.22 | LEDLLATVKYYVEHP | 73.19 | PTHPESDEKENHVGL | 88.62 |
| DSKLAKQESLSHKLG | 70.50 | AKKVPLDRMPYNLQY | 62.98 | EDLLATVKYYVEHPN | 78.23 | THPESDEKENHVGLN | 76.27 |
| SKLAKQESLSHKLGA | 76.16 | VPLDRMPYNLQYTVE | 71.53 | DLLATVKYYVEHPNE | 76.15 | HPESDEKENHVGLNP | 74.54 |
| KLAKQESLSHKLGAK | 65.82 | PLDRMPYNLQYTVEV | 72.03 | LLATVKYYVEHPNER | 66.77 | PESDEKENHVGLNPS | 77.70 |
| LAKQESLSHKLGAKK | 65.00 | LDRMPYNLQYTVEVK | 77.77 | ATVKYYVEHPNERPH | 71.91 | ESDEKENHVGLNPSA | 76.47 |
| SHKLGAKKTDLPSSD | 64.17 | DRMPYNLQYTVEVKN | 72.07 | TVKYYVEHPNERPHS | 73.60 | SDEKENHVGLNPSAD | 73.15 |
| LGAKKTDLPSSDREF | 65.00 | RMPYNLQYTVEVKNG | 71.80 | VKYYVEHPNERPHSD | 74.39 | DEKENHVGLNPSADN | 60.92 |
| TDLPSSDREFYNKAY | 63.93 | MPYNLQYTVEVKNGS | 72.71 | KYYVEHPNERPHSDN | 60.65 | PSADNLYKPSTDTEE | 61.39 |
| EFYNKAYDLLARIHQ | 64.42 | PYNLQYTVEVKNGSL | 66.99 | VEHPNERPHSDNGFG | 65.58 | TDEAEIPQVEHSVIN | 61.07 |
| FYNKAYDLLARIHQD | 63.06 | YNLQYTVEVKNGSLI | 63.80 | ASDHVQRNKNGQADT | 63.73 | IRQNAVETLTGLKSS | 69.63 |
| YNKAYDLLARIHQDL | 71.92 | NLQYTVEVKNGSLII | 62.61 | SDHVQRNKNGQADTN | 80.34 | RQNAVETLTGLKSSL | 78.04 |
| NKAYDLLARIHQDLL | 62.76 | LQYTVEVKNGSLIIP | 61.58 | DHVQRNKNGQADTNQ | 69.28 | QNAVETLTGLKSSLL | 68.19 |
| KAYDLLARIHQDLLD | 62.81 | QYTVEVKNGSLIIPH | 64.34 | QRNKNGQADTNQTEK | 71.60 | LKSSLLLGTKDNNTI | 61.49 |
| AYDLLARIHQDLLDN | 68.17 | YTVEVKNGSLIIPHY | 64.80 | RNKNGQADTNQTEKP | 70.27 | KSSLLLGTKDNNTIS | 60.55 |
| YDLLARIHQDLLDNK | 68.29 | TVEVKNGSLIIPHYD | 64.16 | NKNGQADTNQTEKPN | 71.94 | SSLLLGTKDNNTISA | 63.19 |
| DLLARIHQDLLDNKG | 74.91 | VEVKNGSLIIPHYDH | 61.67 | KNGQADTNQTEKPNE | 68.52 | SLLLGTKDNNTISAE | 61.01 |
| LLARIHQDLLDNKGR | 71.56 | EVKNGSLIIPHYDHY | 62.31 | NGQADTNQTEKPNEE | 60.57 | LLLGTKDNNTISAEV | 66.68 |
| LARIHQDLLDNKGRQ | 78.06 | VKNGSLIIPHYDHYH | 61.98 | EEKPQTEKPEEDKEH | 62.24 | LLGTKDNNTISAEVD | 76.60 |
| ARIHQDLLDNKGRQV | 69.86 | IIPHYDHYHNIKFEW | 72.02 | EKPQTEKPEEDKEHD | 61.25 | LGTKDNNTISAEVDS | 78.98 |
| IHQDLLDNKGRQVDF | 60.62 | IPHYDHYHNIKFEWF | 64.10 | KPQTEKPEEDKEHDE | 73.39 | GTKDNNTISAEVDSL | 80.97 |
| HQDLLDNKGRQVDFE | 65.15 | PHYDHYHNIKFEWFD | 68.93 | PQTEKPEEDKEHDEV | 72.55 | TKDNNTISAEVDSLL | 73.94 |
| ALDNLLERLKDVSSD | 61.23 | HYDHYHNIKFEWFDE | 68.13 | QTEKPEEDKEHDEVS | 74.97 | KDNNTISAEVDSLLA | 66.53 |
| LDNLLERLKDVSSDK | 73.89 | YDHYHNIKFEWFDEG | 72.78 | TEKPEEDKEHDEVSE | 62.91 | LALLKESQPTPIQ | 62.40 |
| DNLLERLKDVSSDKV | 69.96 | DHYHNIKFEWFDEGL | 70.32 | KPEEDKEHDEVSEPT | 68.83 | ALLKESQPTPIQ | 64.24 |
| NLLERLKDVSSDKVK | 67.03 | IKFEWFDEGLYEAPK | 67.72 | PEEDKEHDEVSEPTH | 68.26 |  |  |
| LERLKDVSSDKVKLV | 62.39 | KFEWFDEGLYEAPKG | 60.93 | EEDKEHDEVSEPTHP | 71.35 |  |  |
| ABCpred | | | | | | | |
| Sequence | **Score** | **Sequence** | **Score** | **Sequence** | **Score** | **Sequence** | **Score** |
| EKPQTEKPEEDKEHDE | 0.95 | FGNASDHVQRNKNGQA | 0.87 | HVQRNKNGQADTNQTE | 0.80 | GQADTNQTEKPNEEKP | 0.72 |
| DPRDITSDEGDAYVTP | 0.95 | TDLPSSDREFYNKAYD | 0.86 | PEEDKEHDEVSEPTHP | 0.79 | AYAKEKGLTPPSTDHQ | 0.71 |
| TDHQDSGNTEAKGAEA | 0.93 | HSVINAKIAEAEALLE | 0.86 | NGVPRYIPAKDLSAET | 0.79 | LLGTKDNNTISAEVDS | 0.69 |
| EVSEPTHPESDEKENH | 0.92 | EGLYEAPKGYSLEDLL | 0.86 | SHWIKKDSLSEAERAA | 0.79 | SSIRQNAVETLTGLKS | 0.69 |
| HYHNIKFEWFDEGLYE | 0.92 | GAEAIYNRVKAAKKVP | 0.85 | KPNAQITYTDDEIQVA | 0.79 | SAEVDSLLALLKESQP | 0.66 |
| GLTPPSTDHQDSGNTE | 0.91 | ERAAAQAYAKEKGLTP | 0.85 | LARIHQDLLDNKGRQV | 0.76 | ERLKDVSSDKVKLVDD | 0.66 |
| KYTTEDGYIFDPRDIT | 0.89 | TGLKSSLLLGTKDNNT | 0.84 | EEAEDTTDEAEIPQVE | 0.76 | KAAKKVPLDRMPYNLQ | 0.65 |
| LAPIRHPERLGKPNAQ | 0.89 | SDEGDAYVTPHMTHSH | 0.84 | YVTPHMTHSHWIKKDS | 0.75 | RMPYNLQYTVEVKNGS | 0.60 |
| VRKVGDGYVFEENGVP | 0.88 | DFEALDNLLERLKDVS | 0.83 | GLNPSADNLYKPSTDT | 0.74 | KGYSLEDLLATVKYYV | 0.53 |
| VEHPNERPHSDNGFGN | 0.88 | PSTDTEETEEEAEDTT | 0.83 | SDEKENHVGLNPSADN | 0.74 |  |  |
| GSLIIPHYDHYHNIKF | 0.88 | KLAKQESLSHKLGAKK | 0.81 | LSAETAAGIDSKLAKQ | 0.74 |  |  |
| DDEIQVAKLAGKYTTE | 0.88 | AEALLEKVTDSSIRQN | 0.81 | EIPQVEHSVINAKIAE | 0.72 |  |  |
| Emini surface accessibility Prediction | | | | | | | |
| Sequence Sequence Sequence Sequence | | | | | | | |
| SSDREFYNKA | | PSTDHQDSG | | HPNERPHS | | HVQRNKNG | |
| DTNQTEKPNEEKPQTEKPEEDKEH | | PTHPESDEKE | | NLYKPSTDTEETEEEAEDTTD | |  | |
|  |  |  |  |  |  |  |  |
| Ellipro | | | | | | | |
| Sequence | **Score** | **Sequence** | **Score** | **Sequence** | **Score** | **Sequence** | **Score** |
| LEKVTDSSIRQNAVETLTGLKSSLLLGTKDNNTISAEVDSLLALLKESQPTPIQ | 0.826 | EAKGAEAIYNRVKAAK | 0.638 | SDHVQRNKNGQADTNQTEK | 0.605 | EPTH | 0.555 |
| EKLVKEAVRKVGDGYVFEENGVPRYIPAKDLSAETAAGIDSKLAKQESL | 0.801 | EHPNERPHSDNGF | 0.611 | DLLDNKGRQVDFEALDNLLERLKDVSSDKVKLVDDILAFLAPIR | 0.589 | LIIPHYDH | 0.546 |
| TEETEEEAEDTTDEAEIPQVEH | 0.79 | KTDLPSSDREFYN | 0.608 | AKEKGL | 0.558 | DEGLY | 0.527 |

**Supplementary Table 3**. Prediction of linear B-cell epitopes from PsaA by LBTope, ABCpred, Emini, and Ellipro.

| LBTope | | | | | | | | |
| --- | --- | --- | --- | --- | --- | --- | --- | --- |
| Sequence | **% Probability of correct prediction** | **Sequence** | | **% Probability of correct prediction** | **Sequence** | **% Probability of correct prediction** | **Sequence** | **% Probability of correct prediction** |
| ADITKNIAGDKIDLH | 61.53 | QDPHEYEPLPEDVKK | | 75.44 | YLEGQNEKGKEDPHA | 72.70 | AKDPNNKEFYEKNLK | 64.66 |
| DITKNIAGDKIDLHS | 63.45 | DPHEYEPLPEDVKKT | | 72.16 | LEGQNEKGKEDPHAW | 73.93 | EFYEKNLKEYTDKLD | 62.29 |
| VPIGQDPHEYEPLPE | 66.51 | AKKTENKDYFAVSDG | | 61.53 | EGQNEKGKEDPHAWL | 71.20 | PSAYIWEINTEEEGT | 60.95 |
| PIGQDPHEYEPLPED | 82.80 | DVIYLEGQNEKGKED | | 76.97 | GQNEKGKEDPHAWLN | 64.62 | SAYIWEINTEEEGTP | 63.92 |
| IGQDPHEYEPLPEDV | 86.55 | VIYLEGQNEKGKEDP | | 76.37 | EKGKEDPHAWLNLEN | 62.25 | KLRQTKVPSLFVESS | 64.82 |
| GQDPHEYEPLPEDVK | 79.71 | IYLEGQNEKGKEDPH | | 74.49 | KNIAKQLSAKDPNNK | 66.84 |  |  |
| ABCpred | | | | | | | | |
| Sequence | **Score** | **Sequence** | | **Score** | **Sequence** | **Score** | **Sequence** | **Score** |
| GKEGDSYYSMMKYNLD | 0.95 | KKTSEADLIFYNGINL | | 0.84 | NIPIYAQIFTDSIAEQ | 0.77 | DPHAWLNLENGIIFAK | 0.69 |
| DPHEYEPLPEDVKKTS | 0.94 | SLFVESSVDDRPMKTV | | 0.83 | NGIIFAKNIAKQLSAK | 0.76 | KLVENAKKTENKDYFA | 0.68 |
| GNAWFTKLVENAKKTE | 0.91 | NSIIADITKNIAGDKI | | 0.83 | DDRPMKTVSQDTNIPI | 0.75 | AVSDGVDVIYLEGQNE | 0.67 |
| IWEINTEEEGTPEQIK | 0.90 | TSEGAFKYFSKAYGVP | | 0.83 | KNLKEYTDKLDKLDKE | 0.72 | TVSQDTNIPIYAQIFT | 0.67 |
| CASGKKDTTSGQKLKV | 0.88 | IVPIGQDPHEYEPLPE | | 0.80 | SMMKYNLDKIAEGLAK | 0.71 | YNGINLETGGNAWFTK | 0.65 |
| GQNEKGKEDPHAWLNL | 0.87 | TPEQIKTLVEKLRQTK | | 0.79 | AKQLSAKDPNNKEFYE | 0.70 | TENKDYFAVSDGVDVI | 0.62 |
| SKAYGVPSAYIWEINT | 0.85 | GDKIDLHSIVPIGQDP | | 0.78 | KLDKESKDKFNKIPAE | 0.69 | DTTSGQKLKVVATNSI | 0.58 |
| Emini surface accessibility Prediction | | | | | | | | |
| Sequence Sequence Sequence | | | | | | | | |
| GKKDTTS | | NAKKTENKD | | | KNLKEYTDKLDKLDKESKDK | |  | |
| DPHEYEPL | | GQNEKGKED | | | NTEEEGT | |  | |
| DVKKTS | | KDPNNKEFY | | | QGKEGDSY | |  | |
|  |  |  | |  |  |  |  |  |
| Ellipro | | | | | | | | |
| Sequence | **Score** | | **Sequence** | **Score** | **Sequence** | **Score** | **Sequence** | **Score** |
| KKDTTSGQKLK | 0.839 | | TKLVENAKKTENK | 0.73 | GGNA | 0.656 | EQGKEG | 0.581 |
| KESKDKFNKIPAEKKL | 0.745 | | YEPLPEDVKKTSEAD | 0.664 | GDKID | 0.627 |  |  |
| EEEGTPEQIKTLVEKLRQTKVPS | 0.735 | | VDDRPMKTVSQDTNIPIY | 0.663 | VDVIYLEGQNEKGKE | 0.626 |  |  |

**Supplementary Table 4.** Prediction of linear B-cell epitopes from PspC by LBTope, ABCpred, Emini, and Ellipro.

| LBTope | | | | | | | | |
| --- | --- | --- | --- | --- | --- | --- | --- | --- |
| Sequence | **% Probability of correct prediction** | **Sequence** | | **% Probability of correct prediction** | **Sequence** | **% Probability of correct prediction** | **Sequence** | **% Probability of correct prediction** |
| ATENERTTQVPTSSN | 66.65 | PEKKVAEAQKKVEEA | | 62.86 | IAEFDVEVKKAELEL | 62.39 | EEAEKKAKAQKEEDR | 75.92 |
| TENERTTQVPTSSNR | 84.83 | EKKVAEAQKKVEEAE | | 65.00 | DVEVKKAELELVKEE | 62.52 | EAEKKAKAQKEEDRR | 81.45 |
| ENERTTQVPTSSNRG | 86.10 | KKVAEAQKKVEEAEK | | 65.00 | AKEKVESKKAEATRL | 67.81 | AEKKAKAQKEEDRRN | 78.74 |
| NERTTQVPTSSNRGK | 82.21 | KVAEAQKKVEEAEKK | | 65.00 | AKEQDESKRRTKRAV | 67.87 | EKKAKAQKEEDRRNY | 81.25 |
| ERTTQVPTSSNRGKP | 75.69 | VAEAQKKVEEAEKKA | | 71.65 | KEQDESKRRTKRAVP | 74.52 | KKAKAQKEEDRRNYP | 78.90 |
| RTTQVPTSSNRGKPE | 71.10 | AEAQKKVEEAEKKAK | | 66.98 | ESKRRTKRAVPGELA | 66.75 | KAKAQKEEDRRNYPS | 78.65 |
| TTQVPTSSNRGKPER | 74.07 | EAQKKVEEAEKKAKA | | 74.58 | RRTKRAVPGELATPD | 67.54 | AKAQKEEDRRNYPSN | 77.41 |
| TQVPTSSNRGKPERR | 76.64 | AQKKVEEAEKKAKAQ | | 75.42 | RTKRAVPGELATPDK | 76.09 | KAQKEEDRRNYPSNT | 79.10 |
| QVPTSSNRGKPERRK | 81.25 | QKKVEEAEKKAKAQK | | 67.37 | TKRAVPGELATPDKK | 69.18 | AQKEEDRRNYPSNTY | 82.67 |
| VPTSSNRGKPERRKA | 70.12 | KKVEEAEKKAKAQKE | | 74.42 | KRAVPGELATPDKKE | 74.91 | QKEEDRRNYPSNTYF | 68.08 |
| SNRGKPERRKAAEQF | 62.15 | KVEEAEKKAKAQKEE | | 72.15 | RAVPGELATPDKKEN | 82.06 | EEDRRNYPSNTYFSL | 61.48 |
| NRGKPERRKAAEQFD | 63.85 | TLPTEPEKKVAEAQK | | 66.71 | AVPGELATPDKKEND | 75.88 | EDRRNYPSNTYFSLE | 64.26 |
| RGKPERRKAAEQFDE | 65.12 | VEEAEKKAKAQKEED | | 85.36 | VPGELATPDKKENDA | 87.72 | DRRNYPSNTYFSLEL | 68.12 |
| GKPERRKAAEQFDEY | 61.00 | EEAEKKAKAQKEEDR | | 75.92 | PGELATPDKKENDAE | 83.65 | FSLELEISESDVEVK | 63.86 |
| KPERRKAAEQFDEYI | 65.23 | EAEKKAKAQKEEDRR | | 81.45 | GELATPDKKENDAES | 76.90 | ELEISESDVEVKKAE | 65.53 |
| PERRKAAEQFDEYIN | 72.36 | AEKKAKAQKEEDRRN | | 78.74 | ELATPDKKENDAESS | 84.58 | AKVESKKAVATRLEN | 62.47 |
| ERRKAAEQFDEYINK | 65.01 | EKKAKAQKEEDRRNY | | 81.25 | LATPDKKENDAESSD | 88.66 | ESKKAVATRLENIKT | 65.55 |
| AAEQFDEYINKMIQL | 61.26 | KKAKAQKEEDRRNYP | | 78.90 | ATPDKKENDAESSDS | 95.86 | EEEAKRKAAEEDKVK | 61.12 |
| AEQFDEYINKMIQLD | 64.99 | KAKAQKEEDRRNYPT | | 74.35 | TPDKKENDAESSDSS | 96.75 | EEAKRKAAEEDKVKE | 63.65 |
| EQFDEYINKMIQLDK | 61.50 | AKAQKEEDRRNYPTN | | 80.33 | PDKKENDAESSDSSV | 84.91 | EAKRKAAEEDKVKEK | 63.41 |
| QFDEYINKMIQLDKR | 71.52 | KAQKEEDRRNYPTNT | | 82.64 | DKKENDAESSDSSVG | 81.00 | AEQPQPAPAPQPEKP | 64.49 |
| MIQLDKRKHTQNLAF | 62.08 | AQKEEDRRNYPTNTY | | 82.85 | KKENDAESSDSSVGE | 69.24 | EQPQPAPAPQPEKPT | 67.99 |
| LDKRKHTQNLAFNIK | 62.11 | QKEEDRRNYPTNTYK | | 73.07 | VGEETLPSPSLKSGK | 61.91 | QPQPAPAPQPEKPTE | 72.47 |
| SRIKTEYLNGLKEKS | 63.62 | KEEDRRNYPTNTYKT | | 72.94 | GEETLPSPSLKSGKK | 75.81 | PQPAPAPQPEKPTEE | 67.21 |
| RIKTEYLNGLKEKSE | 61.67 | EEDRRNYPTNTYKTL | | 69.62 | EETLPSPSLKSGKKV | 72.46 | APAPQPEKPTEEPEN | 65.46 |
| IKTEYLNGLKEKSEA | 68.91 | EDRRNYPTNTYKTLE | | 65.60 | ETLPSPSLKSGKKVA | 72.97 | PAPQPEKPTEEPENP | 63.47 |
| KTEYLNGLKEKSEAE | 65.64 | DRRNYPTNTYKTLEL | | 65.55 | TLPSPSLKSGKKVAE | 70.16 | APQPEKPTEEPENPA | 63.47 |
| TEYLNGLKEKSEAEL | 64.31 | LELEIAEFDVEVKKA | | 65.75 | SPSLKSGKKVAEAEK | 62.72 | PQPEKPTEEPENPAP | 63.47 |
| EYLNGLKEKSEAELP | 65.82 | ELEIAEFDVEVKKAE | | 69.40 | KKVEEAEKKAKAQKE | 74.42 |  |  |
| YLNGLKEKSEAELPS | 66.60 | LEIAEFDVEVKKAEL | | 69.40 | KVEEAEKKAKAQKEE | 72.15 |  |  |
| LPTEPEKKVAEAQKK | 66.73 | EIAEFDVEVKKAELE | | 69.40 | VEEAEKKAKAQKEED | 85.36 |  |  |
| ABCpred | | | | | | | | |
| Sequence | **Score** | **Sequence** | | **Score** | **Sequence** | **Score** | **Sequence** | **Score** |
| PERRKAAEQFDEYINK | 0.94 | TEEPENPAPAPKPEKP | | 0.84 | PSKIKAELDAAFKQFK | 0.79 | DKKENDAESSDSSVGE | 0.74 |
| AKEQDESKRRTKRAVP | 0.93 | QKEEDRRNYPSNTYFS | | 0.83 | KEEAKKPLNEGTIKQA | 0.79 | ATRLENIKTDRKKAEE | 0.71 |
| TSSNRGKPERRKAAEQ | 0.92 | PAPQPEKPTEEPENPA | | 0.82 | EGTIKQAKEKVESKKA | 0.78 | TNTYKTLELEIAEFDV | 0.70 |
| KAEEEAKRKADAKEQD | 0.91 | ELVKEEAKEPRNEEKV | | 0.82 | ELEIAEFDVEVKKAEL | 0.78 | VAEAQKKVEEAEKKAK | 0.70 |
| EKKAKAQKEEDRRNYP | 0.90 | ELEISESDVEVKKAEF | | 0.82 | SKKAEATRLENIKTDR | 0.77 | AEKKVEEAEKKAKAQK | 0.67 |
| RAVPGELATPDKKEND | 0.90 | KAEEEAKRKAAEEDKV | | 0.81 | QKEEDRRNYPTNTYKT | 0.77 | PSLKSGKKVAEAEKKV | 0.67 |
| SKRRTKRAVPGELATP | 0.90 | DEYINKMIQLDKRKHT | | 0.81 | VGEETLPSPSLKSGKK | 0.76 | PRNEEKVKQAKAKVES | 0.59 |
| EKKAKAQKEEDRRNYP | 0.90 | ERTTQVPTSSNRGKPE | | 0.80 | AELELVKEEAKKPLNE | 0.76 | KTEYLNGLKEKSEAEL | 0.58 |
| DTLPTEPEKKVAEAQK | 0.87 | IKLSRIKTEYLNGLKE | | 0.80 | KAAEEDKVKEKPAEQP | 0.75 | KRKHTQNLAFNIKLSR | 0.57 |
| KSEAELPSKIKAELDA | 0.84 | VKQAKAKVESKKAVAT | | 0.80 | EKPAEQPQPAPAPQPE | 0.74 |  |  |
| Emini surface accessibility Prediction | | | | | | | | |
| Sequence Sequence Sequence Sequence | | | | | | | | |
| RGKPERRKA | | EAKRKAD | | | AEKKAKAQKEEDRRNYPS | | KPAEQP | |
| AEKKAKAQKEEDRRNYPTNT | | KEQDESKRRT | | | EAKEPRNEEK | | QPEKPTEEPE | |
| TDRKKA | | PDKKEN | | | TDRKKA | |  | |
|  |  |  | |  |  |  |  |  |
| Ellipro | | | | | | | | |
| Sequence | **Score** | | **Sequence** | **Score** | **Sequence** | **Score** | **Sequence** | **Score** |
| DKVKEKPAEQPQPAPAPQPEKPTEEPENPAPAPKPEKPAEQPQ | 0.847 | | RKADAKEQDESKRRTKRAVPGELATPDKKENDAESSDSSVGEETLPSPSLKSGKKVAEAEKKVEEAEKKAKAQKEEDRRNYPSNTYFS | 0.74 | NKMIQLDKRKHTQN | 0.682 | KAEFELVKEEAKEPRNEEKV | 0.643 |
| HATENERTT | 0.753 | | FKQFKKDTLPTEPEKK | 0.683 | EAKKPLNEGT | 0.642 |  |  |

**Supplementary Table 5.** Prediction of conformational epitopes from PhtD-C via Ellipro and Discotope.

| Ellipro |  |  |
| --- | --- | --- |
| Residues | **Number of residues** | **Score** |
| E1, K2, L3, V4, K5, E6 | 6 | 0.975 |
| E298, H299, P300, N301, E302, R303, P304, H305, S306, D307, N308, G309, F310, N334, E354, P355, T356, H357, P358, H366, V367, G368, L369, T383, E384, E385, T386, E387, E388, E389, A390, E391, D392, T393, T394, D395, E396, A397, E398, I399, P400, Q401, V402, E403, H404, S405, N408, L418, E419, K420, V421, T422, D423, S424, S425, I426, R427, Q428, N429, A430, V431, E432, T433, L434, T435, G436, L437, K438, S439, S440, L441, L442, L443, G444, T445, K446, D447, N448, N449, T450, I451, S452, A453, E454, V455, D456, S457, L458, L459, A460, L461, L462, K463, E464, S465, Q466, P467, T468, P469, I470, Q471 | 101 | 0.746 |
| A7, V8, K10, V11, G12, D13, G14, Y15, V16, F17, E18, E19, N20, G21, V22, P23, R24, Y25, I26, P27, A28, K29, D30, L31, S32, A33, E34, T35, A36, A37, G38, I39, D40, S41, K42, L43, A44, K45, Q46, E47, S48, L49, A55, K56, K57, T58, D59, L60, P61, S62, S63, D64, R65, E66, F67, Y68, N69, K70, K103, D104, V105, S106, S107, D108, K109, K111, L112, V113, D114, D115, I116, A118, F119, L120, A121, P122, I123, H125, E127, Q144, K147 | 81 | 0.667 |
| Y158, P162, R163, I165, T166, K204, G205, L206, T207, G217, N218, E220, A221, K222, G223, A224, E225, A226, I227, Y228, N229, R230, V231, K232, A233, A234, K235, D274, E275, G276, L277, Y278, N322, G323, Q324, A325, D326, T327, N328, Q329, T330, E331, K332 | 43 | 0.608 |
| D81, L82, L83, D84, N85, K86, G87, R88, Q89, V90, D91, F92, E93, A94, L95, D96, N97, L98, L99, R101, E169, A172, Y173, V174, T175, P176, H177, H182, Y200, A201, K202, E203 | 32 | 0.584 |
| S256, L257, I258, I259, P260, H261, Y262, D263, H264, S314, D315, H316, V317, N320, K321 | 15 | 0.526 |
| Discotope |  |  |
| Residues | **Number of residues** | **Score** |
| T175, H177, M178, T179, H180, S181, H182, W183, K185, K186, D187, S188, L189, S190, E191, A192, E193, R194, A195, A196, A197, Q198, A199, Y200, A201, K202, E203, K204, G205, L206, T207, P208, P209, S210, T211, D212, H213, Q214, D215, S216, G217, N218, T219, E220, A221, K222, G223, A224, E225, A226, I227, Y228, N229, R230, V231, K232, A233, A234, K235, K236, V237, P238, L239, D240, R241, M242, P243, Y244, N245, L246, Q247, Y248, T249, V250, E251, V252, K253, N254, G255, S256, L257, I258, I259, P260, H261, Y262, D263, H264, Y265, H266, N267, I268, K269, F270, E271, W272, F273, D274, E275, G276, L277, Y278, E279, A280, P281, K282, G283, Y284, S285, L286, E287, D288, L289, L290, A291, T292, V293, K294, Y295, Y296, V297, E298, H299, P300, N301, E302, R303, P304, H305, S306, D307, N308, G309, F310, G311, N312, A313, S314, D315, H316, V317, Q318, R319, N320, K321, N322, G323, Q324, A325, D326, T327, N328, Q329, T330, E331, K332, P333, N334, E335, E336, K337, P338, Q339, T340, E341, K342, P343, E344, E345, D346, K347, E348, H349, D350, E351, V352, S353, E354, P355, T356, H357, P358, E359, S360, D361, E362, K363, E6364, N365 | 189 | 3.71 |
| N370, S372, A373, D374, N375, L376, Y377, K378, P379 | 9 | -0.85 |
| T386, E387, E388, E389, A390, E391, D392, T393, T394, D395, E396, E398 | 12 | -0.89 |
| E19, G21, V22, P23, R24, Y25 | 6 | -1.01 |
| K45, E47, S48, L49, S50 | 5 | -1.49 |
| K86, G87, R88, Q89 | 4 | -1.53 |
| F67, N69, K70, A71 | 4 | -1.80 |
| D141, I143, V145, A146, L148, G150, Y152, T154, E155, D156, G157, Y158, I159, F160, D161, P162, R163, D164, I165, T166, D168, E169 | 22 | -1.86 |
| I123, R124, H125, E127, R128, L129 | 6 | -1.98 |
| E1, K2, L3, V4, K5, E6, A7, V8 | 8 | -2.22 |
| D447, N448, N449, T450, I451, S452, A453, E454 | 8 | -2.33 |

**Supplementary Table 6.** Prediction of conformational epitopes from PsaA via Ellipro and Discotope.

| Ellipro |  |  |
| --- | --- | --- |
| Residues | **Number of residues** | **Score** |
| K24, K25, D26, T27, T28, S29, G30, Q31, K32, K34, G51, D52, K53, I54, D55, Y69, E70, P71, L72, P73, E74, V76, K77, K78, S80, E81, D83, T94, G95, G96, N97, A98, T101, K102, V104, E105, N106, A107, K108, K109, T110, E111, N112, K113, K156, S159, A160, K161, D162, P163, N164, N165, K166, E167, F168, E170, K171, N172, K174, E175, D178, D181, K185 | 63 | 0.745 |
| D189, K190, F191, I194, P195, A196, E197, K198, K199, G216, V217, E228, E229, E230, G231, T232, P233, E234, Q235, I236, K237, T238, V240, E241, K242, L243, R244, Q245, T246, K247, V248, P249, S250, V257, D258, D259, R260, P261, K263, T264, V265, S266, Q267, D268, T269, N270, I271, P272, Y274, A304, E305, G306, A308, K309 | 54 | 0.715 |
| N91, G121, V122, D123, V124, I125, Y126, L127, E128, G129, Q130, N131, E132, K133, G134, K135, E136, E224 | 18 | 0.578 |
| A283, E284, Q285, G286, K287, D290 | 6 | 0.567 |
| Discotope |  |  |
| Residues | **Number of residues** | **Score** |
| K5, K6, D7, T8, T9, S10, G11, Q12, K13 | 9 | 4.013 |
| E109, G110, Q111, N112, E113, K114, G115, K116 | 8 | 2.62 |
| S140, A141, K142, D143, P144, N145, N146, K147, E148, F149, Y150, E151, K152, N153, K155, E156, D159, K160, D162, K163, K166, E167, D170, N173, K174 | 25 | 2.04 |
| G76, G77, N78, A79 | 4 | 1.814 |
| T208, E209, E210, E211, G212, T213, P214, E215, Q216, I217, K218 | 11 | 1.414 |
| E265, Q266, G267, K268 | 4 | -0.34 |
| D239, D240, R241, K244, T245, Q248, D249, N251 | 8 | -1.91 |

**Supplementary Table 7.** Prediction of conformational epitopes from PspC via Ellipro and Discotope

| Ellipro |  |  |
| --- | --- | --- |
| Residues | **Number of residues** | **Score** |
| E377, K378, P379, T380, E381, E382, P383, E384, N385, P386, A387, P388, A389, P390, K391, P392, E393, K394, P395, A396, E397, Q398, P399, Q400 | 24 | 0.966 |
| Q367, P368, Q369, P370, A371, P372, A373, P374, Q375, P376 | 10 | 0.799 |
| A201, D202, A203, K204, E205, Q206, D207, E208, S209, K210, R211, R212 | 12 | 0.796 |
| H1, A2, T3, E4, N5, E6, R7, T8, T9, Q10 | 10 | 0.722 |
| T213, K214, A216, V217, P218, G219, E220, L221, A222, T223, P224, D225, K226, K227, E228, N229, D230, A231, E232, S233, S234, D235, S236, S237, V238, G239, E240, E241, T242, L243, P244, S245, P246, S247, L248, K249, S250, G251, K252, K253, V254, A255, E256, A257, E258, K259, K260, V261, E262, E263, A264, E265, K266, K267, A268, K269, A270, Q271, K272, E273, E274, D275, R276, R277, N278, Y279, P280, S281, N282, T283, Y284, F285, S286, L289, E293, V296, K300, A301, F303, E304, L305, V306, K307, E308, E309, A310, K311, E312, P313, R314, N315, E316, E317, K318, V319 | 95 | 0.71 |
| E31, N34, K35, M36, I37, Q38, L39, D40, K41, R42, K43, H44, T45, Q46, N47, F85, K86, Q87, F88, K89, K90, D91, T92, L93, P94, T95, E96, P97, E98, K99, K100, E156, A157, K158 | 34 | 0.675 |
| K159, P160, L161, N162, E163, G164, T165 | 7 | 0.643 |
| D358, V360, K361, E362, K363, P364, A365, E366 | 8 | 0.598 |
| Discotope |  |  |
| Residues | **Number of residues** | **Score** |
| [H](https://webs.iiitd.edu.in/raghava/lbtope/vrindex/lbtope.php?ran=65471)1, [A](https://webs.iiitd.edu.in/raghava/lbtope/vrindex/lbtope.php?ran=65471)2, [T](https://webs.iiitd.edu.in/raghava/lbtope/vrindex/lbtope.php?ran=65471)3, [E](https://webs.iiitd.edu.in/raghava/lbtope/vrindex/lbtope.php?ran=65471)4, [N](https://webs.iiitd.edu.in/raghava/lbtope/vrindex/lbtope.php?ran=65471)5, [E](https://webs.iiitd.edu.in/raghava/lbtope/vrindex/lbtope.php?ran=65471)6, [R](https://webs.iiitd.edu.in/raghava/lbtope/vrindex/lbtope.php?ran=65471)7, [T](https://webs.iiitd.edu.in/raghava/lbtope/vrindex/lbtope.php?ran=65471)8, [T](https://webs.iiitd.edu.in/raghava/lbtope/vrindex/lbtope.php?ran=65471)9, [Q](https://webs.iiitd.edu.in/raghava/lbtope/vrindex/lbtope.php?ran=65471)10, [V](https://webs.iiitd.edu.in/raghava/lbtope/vrindex/lbtope.php?ran=65471)11, [P](https://webs.iiitd.edu.in/raghava/lbtope/vrindex/lbtope.php?ran=65471)12, [T](https://webs.iiitd.edu.in/raghava/lbtope/vrindex/lbtope.php?ran=65471)13, [S](https://webs.iiitd.edu.in/raghava/lbtope/vrindex/lbtope.php?ran=65471)14, [S](https://webs.iiitd.edu.in/raghava/lbtope/vrindex/lbtope.php?ran=65471)15, [N](https://webs.iiitd.edu.in/raghava/lbtope/vrindex/lbtope.php?ran=65471)16, [R](https://webs.iiitd.edu.in/raghava/lbtope/vrindex/lbtope.php?ran=65471)17, [G](https://webs.iiitd.edu.in/raghava/lbtope/vrindex/lbtope.php?ran=65471)18, [K](https://webs.iiitd.edu.in/raghava/lbtope/vrindex/lbtope.php?ran=65471)19, [P](https://webs.iiitd.edu.in/raghava/lbtope/vrindex/lbtope.php?ran=65471)20, [E](https://webs.iiitd.edu.in/raghava/lbtope/vrindex/lbtope.php?ran=65471)21, [R](https://webs.iiitd.edu.in/raghava/lbtope/vrindex/lbtope.php?ran=65471)22, [R](https://webs.iiitd.edu.in/raghava/lbtope/vrindex/lbtope.php?ran=65471)23, [K](https://webs.iiitd.edu.in/raghava/lbtope/vrindex/lbtope.php?ran=65471)24, [A](https://webs.iiitd.edu.in/raghava/lbtope/vrindex/lbtope.php?ran=65471)25, [A](https://webs.iiitd.edu.in/raghava/lbtope/vrindex/lbtope.php?ran=65471)26, [E](https://webs.iiitd.edu.in/raghava/lbtope/vrindex/lbtope.php?ran=65471)27, [Q](https://webs.iiitd.edu.in/raghava/lbtope/vrindex/lbtope.php?ran=65471)28, [F](https://webs.iiitd.edu.in/raghava/lbtope/vrindex/lbtope.php?ran=65471)29, [D](https://webs.iiitd.edu.in/raghava/lbtope/vrindex/lbtope.php?ran=65471)30, [E](https://webs.iiitd.edu.in/raghava/lbtope/vrindex/lbtope.php?ran=65471)31 | 31 | 6.584 |
| [N](https://webs.iiitd.edu.in/raghava/lbtope/vrindex/lbtope.php?ran=65471)34, [K](https://webs.iiitd.edu.in/raghava/lbtope/vrindex/lbtope.php?ran=65471)35, [I](https://webs.iiitd.edu.in/raghava/lbtope/vrindex/lbtope.php?ran=65471)37, [Q](https://webs.iiitd.edu.in/raghava/lbtope/vrindex/lbtope.php?ran=65471)38, [L](https://webs.iiitd.edu.in/raghava/lbtope/vrindex/lbtope.php?ran=65471)39, [D](https://webs.iiitd.edu.in/raghava/lbtope/vrindex/lbtope.php?ran=65471)40, K41, R42, K43, H44, T45, Q46, N47, L48 | 14 | 3.01 |
| [L](https://webs.iiitd.edu.in/raghava/lbtope/vrindex/lbtope.php?ran=65471)62, [N](https://webs.iiitd.edu.in/raghava/lbtope/vrindex/lbtope.php?ran=65471)63, [G](https://webs.iiitd.edu.in/raghava/lbtope/vrindex/lbtope.php?ran=65471)64, [L](https://webs.iiitd.edu.in/raghava/lbtope/vrindex/lbtope.php?ran=65471)65,[K](https://webs.iiitd.edu.in/raghava/lbtope/vrindex/lbtope.php?ran=65471)66, [E](https://webs.iiitd.edu.in/raghava/lbtope/vrindex/lbtope.php?ran=65471)67, [K](https://webs.iiitd.edu.in/raghava/lbtope/vrindex/lbtope.php?ran=65471)68, [S](https://webs.iiitd.edu.in/raghava/lbtope/vrindex/lbtope.php?ran=65471)69, [E](https://webs.iiitd.edu.in/raghava/lbtope/vrindex/lbtope.php?ran=65471)70, [A](https://webs.iiitd.edu.in/raghava/lbtope/vrindex/lbtope.php?ran=65471)71, [E](https://webs.iiitd.edu.in/raghava/lbtope/vrindex/lbtope.php?ran=65471)72, [L](https://webs.iiitd.edu.in/raghava/lbtope/vrindex/lbtope.php?ran=65471)73, [P](https://webs.iiitd.edu.in/raghava/lbtope/vrindex/lbtope.php?ran=65471)74, [S](https://webs.iiitd.edu.in/raghava/lbtope/vrindex/lbtope.php?ran=65471)75 | 14 | 3.511 |
| [K](https://webs.iiitd.edu.in/raghava/lbtope/vrindex/lbtope.php?ran=65471)86, [F](https://webs.iiitd.edu.in/raghava/lbtope/vrindex/lbtope.php?ran=65471)88, [K](https://webs.iiitd.edu.in/raghava/lbtope/vrindex/lbtope.php?ran=65471)89, [K](https://webs.iiitd.edu.in/raghava/lbtope/vrindex/lbtope.php?ran=65471)90, [D](https://webs.iiitd.edu.in/raghava/lbtope/vrindex/lbtope.php?ran=65471)91, [T](https://webs.iiitd.edu.in/raghava/lbtope/vrindex/lbtope.php?ran=65471)92, [L](https://webs.iiitd.edu.in/raghava/lbtope/vrindex/lbtope.php?ran=65471)93, [P](https://webs.iiitd.edu.in/raghava/lbtope/vrindex/lbtope.php?ran=65471)94, [T](https://webs.iiitd.edu.in/raghava/lbtope/vrindex/lbtope.php?ran=65471)95, [E](https://webs.iiitd.edu.in/raghava/lbtope/vrindex/lbtope.php?ran=65471)96, [P](https://webs.iiitd.edu.in/raghava/lbtope/vrindex/lbtope.php?ran=65471)97, [E](https://webs.iiitd.edu.in/raghava/lbtope/vrindex/lbtope.php?ran=65471)98, [K](https://webs.iiitd.edu.in/raghava/lbtope/vrindex/lbtope.php?ran=65471)99, [K](https://webs.iiitd.edu.in/raghava/lbtope/vrindex/lbtope.php?ran=65471)100, [V](https://webs.iiitd.edu.in/raghava/lbtope/vrindex/lbtope.php?ran=65471)101, [A](https://webs.iiitd.edu.in/raghava/lbtope/vrindex/lbtope.php?ran=65471)102 | 17 | 2.478 |
| [K](https://webs.iiitd.edu.in/raghava/lbtope/vrindex/lbtope.php?ran=65471)116, [A](https://webs.iiitd.edu.in/raghava/lbtope/vrindex/lbtope.php?ran=65471)117, [Q](https://webs.iiitd.edu.in/raghava/lbtope/vrindex/lbtope.php?ran=65471)118, [K](https://webs.iiitd.edu.in/raghava/lbtope/vrindex/lbtope.php?ran=65471)119, [E](https://webs.iiitd.edu.in/raghava/lbtope/vrindex/lbtope.php?ran=65471)120, [E](https://webs.iiitd.edu.in/raghava/lbtope/vrindex/lbtope.php?ran=65471)121, [D](https://webs.iiitd.edu.in/raghava/lbtope/vrindex/lbtope.php?ran=65471)122, [R](https://webs.iiitd.edu.in/raghava/lbtope/vrindex/lbtope.php?ran=65471)123, [R](https://webs.iiitd.edu.in/raghava/lbtope/vrindex/lbtope.php?ran=65471)124, [N](https://webs.iiitd.edu.in/raghava/lbtope/vrindex/lbtope.php?ran=65471)125, [Y](https://webs.iiitd.edu.in/raghava/lbtope/vrindex/lbtope.php?ran=65471)126, [P](https://webs.iiitd.edu.in/raghava/lbtope/vrindex/lbtope.php?ran=65471)127, [T](https://webs.iiitd.edu.in/raghava/lbtope/vrindex/lbtope.php?ran=65471)128, [N](https://webs.iiitd.edu.in/raghava/lbtope/vrindex/lbtope.php?ran=65471)129, [T](https://webs.iiitd.edu.in/raghava/lbtope/vrindex/lbtope.php?ran=65471)130, [Y](https://webs.iiitd.edu.in/raghava/lbtope/vrindex/lbtope.php?ran=65471)131, [K](https://webs.iiitd.edu.in/raghava/lbtope/vrindex/lbtope.php?ran=65471)132, [T](https://webs.iiitd.edu.in/raghava/lbtope/vrindex/lbtope.php?ran=65471)133, [L](https://webs.iiitd.edu.in/raghava/lbtope/vrindex/lbtope.php?ran=65471)134, [E](https://webs.iiitd.edu.in/raghava/lbtope/vrindex/lbtope.php?ran=65471)135, [L](https://webs.iiitd.edu.in/raghava/lbtope/vrindex/lbtope.php?ran=65471)136, [E](https://webs.iiitd.edu.in/raghava/lbtope/vrindex/lbtope.php?ran=65471)137, [I](https://webs.iiitd.edu.in/raghava/lbtope/vrindex/lbtope.php?ran=65471)138 | 23 | 6.037 |
| [K](https://webs.iiitd.edu.in/raghava/lbtope/vrindex/lbtope.php?ran=65471)154, [E](https://webs.iiitd.edu.in/raghava/lbtope/vrindex/lbtope.php?ran=65471)155, [E](https://webs.iiitd.edu.in/raghava/lbtope/vrindex/lbtope.php?ran=65471)156. [A](https://webs.iiitd.edu.in/raghava/lbtope/vrindex/lbtope.php?ran=65471)157. [K](https://webs.iiitd.edu.in/raghava/lbtope/vrindex/lbtope.php?ran=65471)158. [K](https://webs.iiitd.edu.in/raghava/lbtope/vrindex/lbtope.php?ran=65471)159, [P](https://webs.iiitd.edu.in/raghava/lbtope/vrindex/lbtope.php?ran=65471)160, [L](https://webs.iiitd.edu.in/raghava/lbtope/vrindex/lbtope.php?ran=65471)161, [N](https://webs.iiitd.edu.in/raghava/lbtope/vrindex/lbtope.php?ran=65471)162, [E](https://webs.iiitd.edu.in/raghava/lbtope/vrindex/lbtope.php?ran=65471)163, [G](https://webs.iiitd.edu.in/raghava/lbtope/vrindex/lbtope.php?ran=65471)164, [T](https://webs.iiitd.edu.in/raghava/lbtope/vrindex/lbtope.php?ran=65471)165, [I](https://webs.iiitd.edu.in/raghava/lbtope/vrindex/lbtope.php?ran=65471)166, [K](https://webs.iiitd.edu.in/raghava/lbtope/vrindex/lbtope.php?ran=65471)167,[Q](https://webs.iiitd.edu.in/raghava/lbtope/vrindex/lbtope.php?ran=65471)168 | 15 | 1.786 |
| [T](https://webs.iiitd.edu.in/raghava/lbtope/vrindex/lbtope.php?ran=65471)181, [R](https://webs.iiitd.edu.in/raghava/lbtope/vrindex/lbtope.php?ran=65471)182, [L](https://webs.iiitd.edu.in/raghava/lbtope/vrindex/lbtope.php?ran=65471)183, [E](https://webs.iiitd.edu.in/raghava/lbtope/vrindex/lbtope.php?ran=65471)184, [N](https://webs.iiitd.edu.in/raghava/lbtope/vrindex/lbtope.php?ran=65471)185, [I](https://webs.iiitd.edu.in/raghava/lbtope/vrindex/lbtope.php?ran=65471)186, [K](https://webs.iiitd.edu.in/raghava/lbtope/vrindex/lbtope.php?ran=65471)187, [T](https://webs.iiitd.edu.in/raghava/lbtope/vrindex/lbtope.php?ran=65471)188, [D](https://webs.iiitd.edu.in/raghava/lbtope/vrindex/lbtope.php?ran=65471)189, [R](https://webs.iiitd.edu.in/raghava/lbtope/vrindex/lbtope.php?ran=65471)190, [K](https://webs.iiitd.edu.in/raghava/lbtope/vrindex/lbtope.php?ran=65471)191, [K](https://webs.iiitd.edu.in/raghava/lbtope/vrindex/lbtope.php?ran=65471)192, [A](https://webs.iiitd.edu.in/raghava/lbtope/vrindex/lbtope.php?ran=65471)193, [E](https://webs.iiitd.edu.in/raghava/lbtope/vrindex/lbtope.php?ran=65471)194, [E](https://webs.iiitd.edu.in/raghava/lbtope/vrindex/lbtope.php?ran=65471)195, [E](https://webs.iiitd.edu.in/raghava/lbtope/vrindex/lbtope.php?ran=65471)196, [A](https://webs.iiitd.edu.in/raghava/lbtope/vrindex/lbtope.php?ran=65471)197, [K](https://webs.iiitd.edu.in/raghava/lbtope/vrindex/lbtope.php?ran=65471)198, [R](https://webs.iiitd.edu.in/raghava/lbtope/vrindex/lbtope.php?ran=65471)199, [K](https://webs.iiitd.edu.in/raghava/lbtope/vrindex/lbtope.php?ran=65471)200, [A](https://webs.iiitd.edu.in/raghava/lbtope/vrindex/lbtope.php?ran=65471)201, [D](https://webs.iiitd.edu.in/raghava/lbtope/vrindex/lbtope.php?ran=65471)202, [A](https://webs.iiitd.edu.in/raghava/lbtope/vrindex/lbtope.php?ran=65471)203, [K](https://webs.iiitd.edu.in/raghava/lbtope/vrindex/lbtope.php?ran=65471)204, [E](https://webs.iiitd.edu.in/raghava/lbtope/vrindex/lbtope.php?ran=65471)205, [Q](https://webs.iiitd.edu.in/raghava/lbtope/vrindex/lbtope.php?ran=65471)206, [D](https://webs.iiitd.edu.in/raghava/lbtope/vrindex/lbtope.php?ran=65471)207, [E](https://webs.iiitd.edu.in/raghava/lbtope/vrindex/lbtope.php?ran=65471)208, [S](https://webs.iiitd.edu.in/raghava/lbtope/vrindex/lbtope.php?ran=65471)209, [K](https://webs.iiitd.edu.in/raghava/lbtope/vrindex/lbtope.php?ran=65471)210, [R](https://webs.iiitd.edu.in/raghava/lbtope/vrindex/lbtope.php?ran=65471)211, [R](https://webs.iiitd.edu.in/raghava/lbtope/vrindex/lbtope.php?ran=65471)212, [T](https://webs.iiitd.edu.in/raghava/lbtope/vrindex/lbtope.php?ran=65471)213, [K](https://webs.iiitd.edu.in/raghava/lbtope/vrindex/lbtope.php?ran=65471)214, [R](https://webs.iiitd.edu.in/raghava/lbtope/vrindex/lbtope.php?ran=65471)215, [A](https://webs.iiitd.edu.in/raghava/lbtope/vrindex/lbtope.php?ran=65471)216, [V](https://webs.iiitd.edu.in/raghava/lbtope/vrindex/lbtope.php?ran=65471)217, [P](https://webs.iiitd.edu.in/raghava/lbtope/vrindex/lbtope.php?ran=65471)218, [G](https://webs.iiitd.edu.in/raghava/lbtope/vrindex/lbtope.php?ran=65471)219, [E](https://webs.iiitd.edu.in/raghava/lbtope/vrindex/lbtope.php?ran=65471)220, [L](https://webs.iiitd.edu.in/raghava/lbtope/vrindex/lbtope.php?ran=65471)221, [A](https://webs.iiitd.edu.in/raghava/lbtope/vrindex/lbtope.php?ran=65471)222, [T](https://webs.iiitd.edu.in/raghava/lbtope/vrindex/lbtope.php?ran=65471)223, [P](https://webs.iiitd.edu.in/raghava/lbtope/vrindex/lbtope.php?ran=65471)224, [D](https://webs.iiitd.edu.in/raghava/lbtope/vrindex/lbtope.php?ran=65471)225, [K](https://webs.iiitd.edu.in/raghava/lbtope/vrindex/lbtope.php?ran=65471)226, [K](https://webs.iiitd.edu.in/raghava/lbtope/vrindex/lbtope.php?ran=65471)227, [E](https://webs.iiitd.edu.in/raghava/lbtope/vrindex/lbtope.php?ran=65471)228, [N](https://webs.iiitd.edu.in/raghava/lbtope/vrindex/lbtope.php?ran=65471)229, [D](https://webs.iiitd.edu.in/raghava/lbtope/vrindex/lbtope.php?ran=65471)230, [A](https://webs.iiitd.edu.in/raghava/lbtope/vrindex/lbtope.php?ran=65471)231, [E](https://webs.iiitd.edu.in/raghava/lbtope/vrindex/lbtope.php?ran=65471)232, [S](https://webs.iiitd.edu.in/raghava/lbtope/vrindex/lbtope.php?ran=65471)233, [S](https://webs.iiitd.edu.in/raghava/lbtope/vrindex/lbtope.php?ran=65471)234, [D](https://webs.iiitd.edu.in/raghava/lbtope/vrindex/lbtope.php?ran=65471)235, [S](https://webs.iiitd.edu.in/raghava/lbtope/vrindex/lbtope.php?ran=65471)236, [S](https://webs.iiitd.edu.in/raghava/lbtope/vrindex/lbtope.php?ran=65471)237, [V](https://webs.iiitd.edu.in/raghava/lbtope/vrindex/lbtope.php?ran=65471)238, [G](https://webs.iiitd.edu.in/raghava/lbtope/vrindex/lbtope.php?ran=65471)239, [E](https://webs.iiitd.edu.in/raghava/lbtope/vrindex/lbtope.php?ran=65471)240, [E](https://webs.iiitd.edu.in/raghava/lbtope/vrindex/lbtope.php?ran=65471)241, [T](https://webs.iiitd.edu.in/raghava/lbtope/vrindex/lbtope.php?ran=65471)242, [L](https://webs.iiitd.edu.in/raghava/lbtope/vrindex/lbtope.php?ran=65471)243, [P](https://webs.iiitd.edu.in/raghava/lbtope/vrindex/lbtope.php?ran=65471)244, [S](https://webs.iiitd.edu.in/raghava/lbtope/vrindex/lbtope.php?ran=65471)245, [P](https://webs.iiitd.edu.in/raghava/lbtope/vrindex/lbtope.php?ran=65471)246, [S](https://webs.iiitd.edu.in/raghava/lbtope/vrindex/lbtope.php?ran=65471)247, [L](https://webs.iiitd.edu.in/raghava/lbtope/vrindex/lbtope.php?ran=65471)248, [K](https://webs.iiitd.edu.in/raghava/lbtope/vrindex/lbtope.php?ran=65471)249, [S](https://webs.iiitd.edu.in/raghava/lbtope/vrindex/lbtope.php?ran=65471)250, [G](https://webs.iiitd.edu.in/raghava/lbtope/vrindex/lbtope.php?ran=65471)251, [K](https://webs.iiitd.edu.in/raghava/lbtope/vrindex/lbtope.php?ran=65471)252, [K](https://webs.iiitd.edu.in/raghava/lbtope/vrindex/lbtope.php?ran=65471)253, [V](https://webs.iiitd.edu.in/raghava/lbtope/vrindex/lbtope.php?ran=65471)254, [A](https://webs.iiitd.edu.in/raghava/lbtope/vrindex/lbtope.php?ran=65471)255, [E](https://webs.iiitd.edu.in/raghava/lbtope/vrindex/lbtope.php?ran=65471)256 | 75 | 9.949 |
| [E](https://webs.iiitd.edu.in/raghava/lbtope/vrindex/lbtope.php?ran=65471)262, [E](https://webs.iiitd.edu.in/raghava/lbtope/vrindex/lbtope.php?ran=65471)263, [A](https://webs.iiitd.edu.in/raghava/lbtope/vrindex/lbtope.php?ran=65471)264, [E](https://webs.iiitd.edu.in/raghava/lbtope/vrindex/lbtope.php?ran=65471)265, [K](https://webs.iiitd.edu.in/raghava/lbtope/vrindex/lbtope.php?ran=65471)266, [K](https://webs.iiitd.edu.in/raghava/lbtope/vrindex/lbtope.php?ran=65471)267, [A](https://webs.iiitd.edu.in/raghava/lbtope/vrindex/lbtope.php?ran=65471)268, [K](https://webs.iiitd.edu.in/raghava/lbtope/vrindex/lbtope.php?ran=65471)269, [A](https://webs.iiitd.edu.in/raghava/lbtope/vrindex/lbtope.php?ran=65471)270, [Q](https://webs.iiitd.edu.in/raghava/lbtope/vrindex/lbtope.php?ran=65471)271, [K](https://webs.iiitd.edu.in/raghava/lbtope/vrindex/lbtope.php?ran=65471)272, [E](https://webs.iiitd.edu.in/raghava/lbtope/vrindex/lbtope.php?ran=65471)273, [E](https://webs.iiitd.edu.in/raghava/lbtope/vrindex/lbtope.php?ran=65471)274, [D](https://webs.iiitd.edu.in/raghava/lbtope/vrindex/lbtope.php?ran=65471)275, [R](https://webs.iiitd.edu.in/raghava/lbtope/vrindex/lbtope.php?ran=65471)276, [R](https://webs.iiitd.edu.in/raghava/lbtope/vrindex/lbtope.php?ran=65471)277, [N](https://webs.iiitd.edu.in/raghava/lbtope/vrindex/lbtope.php?ran=65471)278, [Y](https://webs.iiitd.edu.in/raghava/lbtope/vrindex/lbtope.php?ran=65471)279, [P](https://webs.iiitd.edu.in/raghava/lbtope/vrindex/lbtope.php?ran=65471)280, [S](https://webs.iiitd.edu.in/raghava/lbtope/vrindex/lbtope.php?ran=65471)281, [N](https://webs.iiitd.edu.in/raghava/lbtope/vrindex/lbtope.php?ran=65471)282, [T](https://webs.iiitd.edu.in/raghava/lbtope/vrindex/lbtope.php?ran=65471)283, [Y](https://webs.iiitd.edu.in/raghava/lbtope/vrindex/lbtope.php?ran=65471)284, [F](https://webs.iiitd.edu.in/raghava/lbtope/vrindex/lbtope.php?ran=65471)285, [S](https://webs.iiitd.edu.in/raghava/lbtope/vrindex/lbtope.php?ran=65471)286, [L](https://webs.iiitd.edu.in/raghava/lbtope/vrindex/lbtope.php?ran=65471)287, [E](https://webs.iiitd.edu.in/raghava/lbtope/vrindex/lbtope.php?ran=65471)288, [L](https://webs.iiitd.edu.in/raghava/lbtope/vrindex/lbtope.php?ran=65471)289, [E](https://webs.iiitd.edu.in/raghava/lbtope/vrindex/lbtope.php?ran=65471)290, [I](https://webs.iiitd.edu.in/raghava/lbtope/vrindex/lbtope.php?ran=65471)291, [S](https://webs.iiitd.edu.in/raghava/lbtope/vrindex/lbtope.php?ran=65471)292 | 31 | 4.032 |
| [K](https://webs.iiitd.edu.in/raghava/lbtope/vrindex/lbtope.php?ran=65471)307, [E](https://webs.iiitd.edu.in/raghava/lbtope/vrindex/lbtope.php?ran=65471)308, [E](https://webs.iiitd.edu.in/raghava/lbtope/vrindex/lbtope.php?ran=65471)309, [A](https://webs.iiitd.edu.in/raghava/lbtope/vrindex/lbtope.php?ran=65471)310, [K](https://webs.iiitd.edu.in/raghava/lbtope/vrindex/lbtope.php?ran=65471)311, [E](https://webs.iiitd.edu.in/raghava/lbtope/vrindex/lbtope.php?ran=65471)312, [P](https://webs.iiitd.edu.in/raghava/lbtope/vrindex/lbtope.php?ran=65471)313, [R](https://webs.iiitd.edu.in/raghava/lbtope/vrindex/lbtope.php?ran=65471)314, [N](https://webs.iiitd.edu.in/raghava/lbtope/vrindex/lbtope.php?ran=65471)315, [E](https://webs.iiitd.edu.in/raghava/lbtope/vrindex/lbtope.php?ran=65471)316, [E](https://webs.iiitd.edu.in/raghava/lbtope/vrindex/lbtope.php?ran=65471)317, [K](https://webs.iiitd.edu.in/raghava/lbtope/vrindex/lbtope.php?ran=65471)318, [V](https://webs.iiitd.edu.in/raghava/lbtope/vrindex/lbtope.php?ran=65471)319, [K](https://webs.iiitd.edu.in/raghava/lbtope/vrindex/lbtope.php?ran=65471)320, [Q](https://webs.iiitd.edu.in/raghava/lbtope/vrindex/lbtope.php?ran=65471)321 | 15 | 2.342 |
| [E](https://webs.iiitd.edu.in/raghava/lbtope/vrindex/lbtope.php?ran=65471)327, [S](https://webs.iiitd.edu.in/raghava/lbtope/vrindex/lbtope.php?ran=65471)328, [K](https://webs.iiitd.edu.in/raghava/lbtope/vrindex/lbtope.php?ran=65471)329,[K](https://webs.iiitd.edu.in/raghava/lbtope/vrindex/lbtope.php?ran=65471)330, [A](https://webs.iiitd.edu.in/raghava/lbtope/vrindex/lbtope.php?ran=65471)331, [V](https://webs.iiitd.edu.in/raghava/lbtope/vrindex/lbtope.php?ran=65471)332, [A](https://webs.iiitd.edu.in/raghava/lbtope/vrindex/lbtope.php?ran=65471)333, [T](https://webs.iiitd.edu.in/raghava/lbtope/vrindex/lbtope.php?ran=65471)334, [R](https://webs.iiitd.edu.in/raghava/lbtope/vrindex/lbtope.php?ran=65471)335, [L](https://webs.iiitd.edu.in/raghava/lbtope/vrindex/lbtope.php?ran=65471)336, [E](https://webs.iiitd.edu.in/raghava/lbtope/vrindex/lbtope.php?ran=65471)337, [N](https://webs.iiitd.edu.in/raghava/lbtope/vrindex/lbtope.php?ran=65471)338, [I](https://webs.iiitd.edu.in/raghava/lbtope/vrindex/lbtope.php?ran=65471)339, [K](https://webs.iiitd.edu.in/raghava/lbtope/vrindex/lbtope.php?ran=65471)340, [T](https://webs.iiitd.edu.in/raghava/lbtope/vrindex/lbtope.php?ran=65471)341, [D](https://webs.iiitd.edu.in/raghava/lbtope/vrindex/lbtope.php?ran=65471)342, [R](https://webs.iiitd.edu.in/raghava/lbtope/vrindex/lbtope.php?ran=65471)343, [K](https://webs.iiitd.edu.in/raghava/lbtope/vrindex/lbtope.php?ran=65471)344, [K](https://webs.iiitd.edu.in/raghava/lbtope/vrindex/lbtope.php?ran=65471)345, [A](https://webs.iiitd.edu.in/raghava/lbtope/vrindex/lbtope.php?ran=65471)346, [E](https://webs.iiitd.edu.in/raghava/lbtope/vrindex/lbtope.php?ran=65471)347, [E](https://webs.iiitd.edu.in/raghava/lbtope/vrindex/lbtope.php?ran=65471)348, [E](https://webs.iiitd.edu.in/raghava/lbtope/vrindex/lbtope.php?ran=65471)349, [A](https://webs.iiitd.edu.in/raghava/lbtope/vrindex/lbtope.php?ran=65471)350, [K](https://webs.iiitd.edu.in/raghava/lbtope/vrindex/lbtope.php?ran=65471)351, [R](https://webs.iiitd.edu.in/raghava/lbtope/vrindex/lbtope.php?ran=65471)352, [K](https://webs.iiitd.edu.in/raghava/lbtope/vrindex/lbtope.php?ran=65471)353, [A](https://webs.iiitd.edu.in/raghava/lbtope/vrindex/lbtope.php?ran=65471)354, [A](https://webs.iiitd.edu.in/raghava/lbtope/vrindex/lbtope.php?ran=65471)355, [E](https://webs.iiitd.edu.in/raghava/lbtope/vrindex/lbtope.php?ran=65471)356, [E](https://webs.iiitd.edu.in/raghava/lbtope/vrindex/lbtope.php?ran=65471)357, [D](https://webs.iiitd.edu.in/raghava/lbtope/vrindex/lbtope.php?ran=65471)358, [K](https://webs.iiitd.edu.in/raghava/lbtope/vrindex/lbtope.php?ran=65471)359, [V](https://webs.iiitd.edu.in/raghava/lbtope/vrindex/lbtope.php?ran=65471)360, [K](https://webs.iiitd.edu.in/raghava/lbtope/vrindex/lbtope.php?ran=65471)361, [E](https://webs.iiitd.edu.in/raghava/lbtope/vrindex/lbtope.php?ran=65471)362, [K](https://webs.iiitd.edu.in/raghava/lbtope/vrindex/lbtope.php?ran=65471)363, [P](https://webs.iiitd.edu.in/raghava/lbtope/vrindex/lbtope.php?ran=65471)364, [A](https://webs.iiitd.edu.in/raghava/lbtope/vrindex/lbtope.php?ran=65471)365, [E](https://webs.iiitd.edu.in/raghava/lbtope/vrindex/lbtope.php?ran=65471)366, [Q](https://webs.iiitd.edu.in/raghava/lbtope/vrindex/lbtope.php?ran=65471)367, [P](https://webs.iiitd.edu.in/raghava/lbtope/vrindex/lbtope.php?ran=65471)368, [Q](https://webs.iiitd.edu.in/raghava/lbtope/vrindex/lbtope.php?ran=65471)369, [P](https://webs.iiitd.edu.in/raghava/lbtope/vrindex/lbtope.php?ran=65471)370, [A](https://webs.iiitd.edu.in/raghava/lbtope/vrindex/lbtope.php?ran=65471)371, [P](https://webs.iiitd.edu.in/raghava/lbtope/vrindex/lbtope.php?ran=65471)372, [A](https://webs.iiitd.edu.in/raghava/lbtope/vrindex/lbtope.php?ran=65471)373, [P](https://webs.iiitd.edu.in/raghava/lbtope/vrindex/lbtope.php?ran=65471)374, [Q](https://webs.iiitd.edu.in/raghava/lbtope/vrindex/lbtope.php?ran=65471)375, [P](https://webs.iiitd.edu.in/raghava/lbtope/vrindex/lbtope.php?ran=65471)376, [E](https://webs.iiitd.edu.in/raghava/lbtope/vrindex/lbtope.php?ran=65471)377, [K](https://webs.iiitd.edu.in/raghava/lbtope/vrindex/lbtope.php?ran=65471)378, [P](https://webs.iiitd.edu.in/raghava/lbtope/vrindex/lbtope.php?ran=65471)379, [T](https://webs.iiitd.edu.in/raghava/lbtope/vrindex/lbtope.php?ran=65471)380, [E](https://webs.iiitd.edu.in/raghava/lbtope/vrindex/lbtope.php?ran=65471)381, [E](https://webs.iiitd.edu.in/raghava/lbtope/vrindex/lbtope.php?ran=65471)382, [P](https://webs.iiitd.edu.in/raghava/lbtope/vrindex/lbtope.php?ran=65471)383, [E](https://webs.iiitd.edu.in/raghava/lbtope/vrindex/lbtope.php?ran=65471)384, [N](https://webs.iiitd.edu.in/raghava/lbtope/vrindex/lbtope.php?ran=65471)385, [P](https://webs.iiitd.edu.in/raghava/lbtope/vrindex/lbtope.php?ran=65471)386, [A](https://webs.iiitd.edu.in/raghava/lbtope/vrindex/lbtope.php?ran=65471)387, [P](https://webs.iiitd.edu.in/raghava/lbtope/vrindex/lbtope.php?ran=65471)388, [A](https://webs.iiitd.edu.in/raghava/lbtope/vrindex/lbtope.php?ran=65471)389, [P](https://webs.iiitd.edu.in/raghava/lbtope/vrindex/lbtope.php?ran=65471)390, [K](https://webs.iiitd.edu.in/raghava/lbtope/vrindex/lbtope.php?ran=65471)391, [P](https://webs.iiitd.edu.in/raghava/lbtope/vrindex/lbtope.php?ran=65471)392, [E](https://webs.iiitd.edu.in/raghava/lbtope/vrindex/lbtope.php?ran=65471)393, [K](https://webs.iiitd.edu.in/raghava/lbtope/vrindex/lbtope.php?ran=65471)394, [P](https://webs.iiitd.edu.in/raghava/lbtope/vrindex/lbtope.php?ran=65471)395, [A](https://webs.iiitd.edu.in/raghava/lbtope/vrindex/lbtope.php?ran=65471)396, [E](https://webs.iiitd.edu.in/raghava/lbtope/vrindex/lbtope.php?ran=65471)397, [Q](https://webs.iiitd.edu.in/raghava/lbtope/vrindex/lbtope.php?ran=65471)398, [P](https://webs.iiitd.edu.in/raghava/lbtope/vrindex/lbtope.php?ran=65471)399, [Q](https://webs.iiitd.edu.in/raghava/lbtope/vrindex/lbtope.php?ran=65471)400 | 8 | 5.192 |

**Supplementary Table 8.** Prediction of MHCII epitopes from PhtD-C by IEDB and NetMHCIIpan.

| IEDB | | |  | | | |
| --- | --- | --- | --- | --- | --- | --- |
| Allele | **Peptide** | **Percentile Rank** | **Allele** | **Peptide** | **Percentile Rank** | |
| H2-IEd | AEAIYNRVKAAKKVP | 2.40 | DRB1*11:01 | AEAIYNRVKAAKKVP | 4.50 | |
| H2-IEd | GAEAIYNRVKAAKKV | 3.00 | DRB1*11:01 | EAIYNRVKAAKKVPL | 4.60 | |
| H2-IEd | KGAEAIYNRVKAAKK | 3.25 | DRB1*11:01 | GAEAIYNRVKAAKKV | 4.60 | |
| H2-IEd | EAIYNRVKAAKKVPL | 3.50 | DRB1*03:01 | RDITSDEGDAYVTPH | 4.60 | |
| H2-IAd | QNAVETLTGLKSSLL | 4.30 | DRB1*04:01 | DSKLAKQESLSHKLG | 4.70 | |
| H2-IAd | NAVETLTGLKSSLLL | 4.40 | DRB1*04:01 | SKLAKQESLSHKLGA | 4.70 | |
| H2-IEd | MTHSHWIKKDSLSEA | 4.85 | DRB1*04:01 | IDSKLAKQESLSHKL | 4.80 | |
| H2-IAb | DEGDAYVTPHMTHSH | 5.00 | DRB1*11:01 | AIYNRVKAAKKVPLD | 4.90 | |
| H2-IEd | HMTHSHWIKKDSLSE | 5.00 | DRB1*04:01 | GIDSKLAKQESLSHK | 5.50 | |
| H2-IEd | ILAFLAPIRHPERLG | 5.10 | DRB1*03:01 | SDKVKLVDDILAFLA | 5.50 | |
| H2-IAb | EGDAYVTPHMTHSHW | 5.20 | DRB1*07:01 | DSLLALLKESQPTPI | 5.60 | |
| H2-IEd | AIYNRVKAAKKVPLD | 5.80 | DRB1*13:01 | EKLVKEAVRKVGDGY | 5.70 | |
| H2-IAd | AVETLTGLKSSLLLG | 6.05 | DRB1*13:01 | KLVKEAVRKVGDGYV | 5.70 | |
| H2-IEd | IYNRVKAAKKVPLDR | 6.05 | DRB1*13:01 | LVKEAVRKVGDGYVF | 5.70 | |
| H2-IAb | SDEGDAYVTPHMTHS | 6.20 | DRB1*03:01 | DKVKLVDDILAFLAP | 5.90 | |
| H2-IAd | LSEAERAAAQAYAKE | 6.25 | DRB1*03:01 | KVKLVDDILAFLAPI | 6.20 | |
| H2-IEd | AKGAEAIYNRVKAAK | 6.30 | DRB1*04:01 | GDGYVFEENGVPRYI | 6.30 | |
| H2-IAb | GDAYVTPHMTHSHWI | 6.40 | DRB1*11:01 | IYNRVKAAKKVPLDR | 6.50 | |
| H2-IAd | SEAERAAAQAYAKEK | 6.50 | DRB1*01:01 | LTGLKSSLLLGTKDN | 6.80 | |
| H2-IAb | VDDILAFLAPIRHPE | 6.50 | DRB1*11:01 | KGAEAIYNRVKAAKK | 6.90 | |
| H2-IAb | DDILAFLAPIRHPER | 6.65 | DRB1*03:01 | SSDKVKLVDDILAFL | 6.90 | |
| H2-IEd | YNRVKAAKKVPLDRM | 6.70 | DRB1*15:01 | VKLVDDILAFLAPIR | 7.20 | |
| H2-IAd | SLSEAERAAAQAYAK | 6.85 | DRB1*07:01 | AIYNRVKAAKKVPLD | 7.40 | |
| H2-IEd | LAFLAPIRHPERLGK | 7.20 | DRB1*04:01 | DGYVFEENGVPRYIP | 7.40 | |
| H2-IAb | DAYVTPHMTHSHWIK | 7.70 | DRB1*07:01 | EAIYNRVKAAKKVPL | 7.40 | |
| H2-IAb | DILAFLAPIRHPERL | 7.80 | DRB1*08:01 | AEAIYNRVKAAKKVP | 7.60 | |
| H2-IEd | THSHWIKKDSLSEAE | 8.10 | DRB1*08:01 | AIYNRVKAAKKVPLD | 7.60 | |
| H2-IAd | AIYNRVKAAKKVPLD | 8.15 | DRB1*08:01 | AKGAEAIYNRVKAAK | 7.60 | |
| H2-IEd | NRVKAAKKVPLDRMP | 8.15 | DRB1*08:01 | EAIYNRVKAAKKVPL | 7.60 | |
| H2-IAb | GAEAIYNRVKAAKKV | 8.20 | DRB1*08:01 | GAEAIYNRVKAAKKV | 7.60 | |
| H2-IAb | AEAIYNRVKAAKKVP | 8.25 | DRB1*08:01 | IYNRVKAAKKVPLDR | 7.60 | |
| H2-IEd | DILAFLAPIRHPERL | 8.35 | DRB1*08:01 | KGAEAIYNRVKAAKK | 7.60 | |
| H2-IAd | EAERAAAQAYAKEKG | 8.35 | DRB1*11:01 | DDILAFLAPIRHPER | 8.00 | |
| H2-IAd | VETLTGLKSSLLLGT | 8.35 | DRB1*11:01 | DEIQVAKLAGKYTTE | 8.00 | |
| H2-IEd | AFLAPIRHPERLGKP | 8.65 | DRB1*11:01 | DILAFLAPIRHPERL | 8.00 | |
| H2-IEd | PHMTHSHWIKKDSLS | 8.70 | DRB1*11:01 | ILAFLAPIRHPERLG | 8.00 | |
| H2-IAb | GNTEAKGAEAIYNRV | 8.90 | DRB1*11:01 | LAFLAPIRHPERLGK | 8.00 | |
| H2-IAb | SGNTEAKGAEAIYNR | 8.90 | DRB1*03:01 | VKLVDDILAFLAPIR | 8.00 | |
| H2-IAb | DSGNTEAKGAEAIYN | 8.95 | DRB1*08:01 | EENGVPRYIPAKDLS | 8.10 | |
| H2-IAd | EAIYNRVKAAKKVPL | 9.10 | DRB1*08:01 | ENGVPRYIPAKDLSA | 8.10 | |
| H2-IAb | KGAEAIYNRVKAAKK | 9.10 | DRB1*08:01 | FEENGVPRYIPAKDL | 8.10 | |
| H2-IEd | RVKAAKKVPLDRMPY | 9.50 | DRB1*08:01 | GVPRYIPAKDLSAET | 8.10 | |
| H2-IEd | DDILAFLAPIRHPER | 9.80 | DRB1*08:01 | NGVPRYIPAKDLSAE | 8.10 | |
| H2-IAb | SLLALLKESQPTPIQ | 9.85 | DRB1*03:01 | RLKDVSSDKVKLVDD | 8.10 | |
| H2-IAb | LVDDILAFLAPIRHP | 9.95 | DRB1*08:01 | VFEENGVPRYIPAKD | 8.10 | |
| DRB1*01:01 | AVETLTGLKSSLLLG | 0.44 | DRB1*08:01 | VPRYIPAKDLSAETA | 8.10 | |
| DRB1*01:01 | NAVETLTGLKSSLLL | 0.51 | DRB1*15:01 | ETLTGLKSSLLLGTK | 8.20 | |
| DRB1*01:01 | VETLTGLKSSLLLGT | 0.52 | DRB1*07:01 | GAEAIYNRVKAAKKV | 8.20 | |
| DRB1*01:01 | ETLTGLKSSLLLGTK | 0.96 | DRB1*07:01 | IYNRVKAAKKVPLDR | 8.20 | |
| DRB1*01:01 | QNAVETLTGLKSSLL | 1.60 | DRB1*07:01 | YNRVKAAKKVPLDRM | 8.20 | |
| DRB1*08:01 | HMTHSHWIKKDSLSE | 1.70 | DRB1*04:01 | VGDGYVFEENGVPRY | 8.30 | |
| DRB1*08:01 | HSHWIKKDSLSEAER | 1.70 | DRB1*01:01 | AEAIYNRVKAAKKVP | 8.40 | |
| DRB1*08:01 | HWIKKDSLSEAERAA | 1.70 | DRB1*01:01 | AIYNRVKAAKKVPLD | 8.40 | |
| DRB1*08:01 | MTHSHWIKKDSLSEA | 1.70 | DRB1*01:01 | EAIYNRVKAAKKVPL | 8.40 | |
| DRB1*08:01 | SHWIKKDSLSEAERA | 1.70 | DRB1*01:01 | GAEAIYNRVKAAKKV | 8.40 | |
| DRB1*08:01 | THSHWIKKDSLSEAE | 1.70 | DRB1*01:01 | KGAEAIYNRVKAAKK | 8.40 | |
| DRB1*08:01 | WIKKDSLSEAERAAA | 1.70 | DRB1*15:01 | VETLTGLKSSLLLGT | 8.60 | |
| DRB1*01:01 | TLTGLKSSLLLGTKD | 2.10 | DRB1*15:01 | ILAFLAPIRHPERLG | 8.70 | |
| DRB1*11:01 | YNKAYDLLARIHQDL | 2.50 | DRB1*03:01 | ERLKDVSSDKVKLVD | 8.90 | |
| DRB1*11:01 | FYNKAYDLLARIHQD | 2.60 | DRB1*15:01 | EDLLATVKYYVEHPN | 9.00 | |
| DRB1*11:01 | NKAYDLLARIHQDLL | 2.70 | DRB1*03:01 | NTISAEVDSLLALLK | 9.10 | |
| DRB1*11:01 | KAYDLLARIHQDLLD | 2.90 | DRB1*11:01 | YNRVKAAKKVPLDRM | 9.10 | |
| DRB1*03:01 | DPRDITSDEGDAYVT | 3.00 | DRB1*07:01 | AEAIYNRVKAAKKVP | 9.20 | |
| DRB1*11:01 | EFYNKAYDLLARIHQ | 3.20 | DRB1*11:01 | EKLVKEAVRKVGDGY | 9.20 | |
| DRB1*15:01 | VDDILAFLAPIRHPE | 3.30 | DRB1*03:01 | NNTISAEVDSLLALL | 9.20 | |
| DRB1*15:01 | DDILAFLAPIRHPER | 3.40 | DRB1*11:01 | VDDILAFLAPIRHPE | 9.30 | |
| DRB1*03:01 | PRDITSDEGDAYVTP | 3.40 | DRB1*04:01 | GYVFEENGVPRYIPA | 9.40 | |
| DRB1*11:01 | AYDLLARIHQDLLDN | 3.50 | DRB1*03:01 | LKDVSSDKVKLVDDI | 9.40 | |
| DRB1*11:01 | YDLLARIHQDLLDNK | 3.50 | DRB1*15:01 | AVETLTGLKSSLLLG | 9.70 | |
| DRB1*15:01 | LVDDILAFLAPIRHP | 3.60 | DRB1*03:01 | DNNTISAEVDSLLAL | 9.70 | |
| DRB1*03:01 | FDPRDITSDEGDAYV | 4.10 | DRB1*03:01 | SHWIKKDSLSEAERA | 9.70 | |
| DRB1*15:01 | KLVDDILAFLAPIRH | 4.10 | DRB1*15:01 | EAIYNRVKAAKKVPL | 9.90 | |
| DRB1*15:01 | DILAFLAPIRHPERL | 4.30 | DRB1*03:01 | HSHWIKKDSLSEAER | 9.90 | |
| DRB1*07:01 | SLLALLKESQPTPIQ | 4.30 |  |  |  | |
| NetMHCIIpan |  |  |  |  |  |  |
| Allele | **Peptide** | **%Rank** | **Allele** | **Peptide** | **%Rank** |  |
| H-2-IEd | VPRYIPAKDLSAETA | 0.66 | DRB1_0401 | VGDGYVFEENGVPRY | 0.67 |  |
| H-2-IEd | LATVKYYVEHPNERP | 0.21 | DRB1_0401 | GDGYVFEENGVPRYI | 0.80 |  |
| H-2-IEd | ATVKYYVEHPNERPH | 0.05 | DRB1_0401 | NGVPRYIPAKDLSAE | 0.10 |  |
| H-2-IEd | TVKYYVEHPNERPHS | 0.03 | DRB1_0401 | GVPRYIPAKDLSAET | 0.04 |  |
| H-2-IEd | VKYYVEHPNERPHSD | 0.22 | DRB1_0401 | VPRYIPAKDLSAETA | 0.03 |  |
| H-2-IAd | PRYIPAKDLSAETAA | 0.71 | DRB1_0401 | PRYIPAKDLSAETAA | 0.13 |  |
| H-2-IAd | RYIPAKDLSAETAAG | 0.31 | DRB1_0401 | REFYNKAYDLLARIH | 0.68 |  |
| H-2-IAd | YIPAKDLSAETAAGI | 0.37 | DRB1_0401 | GRQVDFEALDNLLER | 0.73 |  |
| H-2-IAd | IPAKDLSAETAAGID | 0.81 | DRB1_0401 | RQVDFEALDNLLERL | 0.65 |  |
| H-2-IAd | SAETAAGIDSKLAKQ | 0.47 | DRB1_0401 | QVDFEALDNLLERLK | 0.31 |  |
| H-2-IAd | AETAAGIDSKLAKQE | 0.24 | DRB1_0401 | NAQITYTDDEIQVAK | 0.99 |  |
| H-2-IAd | LSEAERAAAQAYAKE | 0.98 | DRB1_0401 | DRMPYNLQYTVEVKN | 0.90 |  |
| H-2-IAd | SEAERAAAQAYAKEK | 0.69 | DRB1_0401 | RMPYNLQYTVEVKNG | 0.52 |  |
| H-2-IAd | GNTEAKGAEAIYNRV | 0.77 | DRB1_0401 | DNGFGNASDHVQRNK | 0.70 |  |
| H-2-IAd | NTEAKGAEAIYNRVK | 0.35 | DRB1_0801 | FYNKAYDLLARIHQD | 0.74 |  |
| H-2-IAd | IPQVEHSVINAKIAE | 0.73 | DRB1_0801 | YNKAYDLLARIHQDL | 0.99 |  |
| H-2-IAd | PQVEHSVINAKIAEA | 0.36 | DRB1_0801 | QVDFEALDNLLERLK | 0.77 |  |
| H-2-IAd | QVEHSVINAKIAEAE | 0.23 | DRB1_0801 | HSHWIKKDSLSEAER | 0.72 |  |
| H-2-IAd | VEHSVINAKIAEAEA | 0.35 | DRB1_1101 | EKLVKEAVRKVGDGY | 0.68 |  |
| H-2-IAd | EHSVINAKIAEAEAL | 0.75 | DRB1_1101 | VPRYIPAKDLSAETA | 0.97 |  |
| H-2-IAd | SIRQNAVETLTGLKS | 0.76 | DRB1_1101 | FYNKAYDLLARIHQD | 0.05 |  |
| DRB1_0101 | PAKDLSAETAAGIDS | 0.78 | DRB1_1101 | YNKAYDLLARIHQDL | 0.06 |  |
| DRB1_0101 | AKDLSAETAAGIDSK | 0.32 | DRB1_1101 | NKAYDLLARIHQDLL | 0.13 |  |
| DRB1_0101 | DRMPYNLQYTVEVKN | 0.33 | DRB1_1101 | KAYDLLARIHQDLLD | 0.83 |  |
| DRB1_0101 | RMPYNLQYTVEVKNG | 0.13 | DRB1_1101 | PYNLQYTVEVKNGSL | 0.65 |  |
| DRB1_0301 | TEDGYIFDPRDITSD | 0.89 | DRB1_1101 | YNLQYTVEVKNGSLI | 0.46 |  |
| DRB1_0301 | EDGYIFDPRDITSDE | 0.72 | DRB1_1101 | NLQYTVEVKNGSLII | 0.55 |  |
| DRB1_0301 | FDPRDITSDEGDAYV | 0.70 | DRB1_1101 | VDSLLALLKESQPTP | 0.90 |  |
| DRB1_0301 | DPRDITSDEGDAYVT | 0.41 | DRB1_1301 | KGAEAIYNRVKAAKK | 0.70 |  |
| DRB1_0301 | PRDITSDEGDAYVTP | 0.30 | DRB1_1301 | GAEAIYNRVKAAKKV | 0.42 |  |
| DRB1_0301 | RDITSDEGDAYVTPH | 0.85 | DRB1_1301 | AEAIYNRVKAAKKVP | 0.21 |  |

**Supplementary Table 9.** Prediction of MHCII epitopes from PsaA by IEDB and NetMHCIIpan.

| **IEDB** | | | | | |
| --- | --- | --- | --- | --- | --- |
| **Allele** | **Peptide** | **Percentile Rank** | **Allele** | **Peptide** | **Percentile Rank** |
| H2-IAb | FKYFSKAYGVPSAYI | 2.00 | DRB1*03:01 | TNSIIADITKNIAGD | 5.50 |
| H2-IAb | KYFSKAYGVPSAYIW | 2.05 | DRB1*03:01 | ATNSIIADITKNIAG | 5.70 |
| H2-IAb | AFKYFSKAYGVPSAY | 2.15 | DRB1*03:01 | VATNSIIADITKNIA | 5.70 |
| H2-IAb | YFSKAYGVPSAYIWE | 2.55 | DRB1*04:01 | GQKLKVVATNSIIAD | 5.90 |
| H2-IAb | FSKAYGVPSAYIWEI | 3.95 | DRB1*04:01 | KLKVVATNSIIADIT | 5.90 |
| H2-IEd | EGAFKYFSKAYGVPS | 5.75 | DRB1*04:01 | QKLKVVATNSIIADI | 5.90 |
| H2-IEd | DSYYSMMKYNLDKIA | 6.10 | DRB1*04:01 | SGQKLKVVATNSIIA | 5.90 |
| H2-IAd | KLKVVATNSIIADIT | 6.24 | DRB1*01:01 | ENGIIFAKNIAKQLS | 6.20 |
| H2-IEd | SEGAFKYFSKAYGVP | 6.25 | DRB1*01:01 | GIIFAKNIAKQLSAK | 6.20 |
| H2-IAd | LKVVATNSIIADITK | 6.60 | DRB1*11:01 | GKEGDSYYSMMKYNL | 6.20 |
| H2-IAd | EKLRQTKVPSLFVES | 6.85 | DRB1*01:01 | IIFAKNIAKQLSAKD | 6.20 |
| H2-IAd | VEKLRQTKVPSLFVE | 6.85 | DRB1*01:01 | LENGIIFAKNIAKQL | 6.20 |
| H2-IAd | GQKLKVVATNSIIAD | 7.06 | DRB1*15:01 | LIFYNGINLETGGNA | 6.20 |
| H2-IAd | QKLKVVATNSIIADI | 7.09 | DRB1*01:01 | NGIIFAKNIAKQLSA | 6.20 |
| H2-IAd | SGQKLKVVATNSIIA | 7.09 | DRB1*11:01 | GIIFAKNIAKQLSAK | 6.40 |
| H2-IEd | GDSYYSMMKYNLDKI | 7.20 | DRB1*04:01 | ADLIFYNGINLETGG | 6.90 |
| H2-IAd | LVEKLRQTKVPSLFV | 7.30 | DRB1*07:01 | LRQTKVPSLFVESSV | 6.90 |
| H2-IEd | SYYSMMKYNLDKIAE | 7.50 | DRB1*03:01 | NSIIADITKNIAGDK | 6.90 |
| H2-IEd | GAFKYFSKAYGVPSA | 8.25 | DRB1*11:01 | IIFAKNIAKQLSAKD | 7.00 |
| H2-IEd | AFKYFSKAYGVPSAY | 8.60 | DRB1*04:01 | EADLIFYNGINLETG | 7.10 |
| H2-IEd | NNKEFYEKNLKEYTD | 9.30 | DRB1*07:01 | ENGIIFAKNIAKQLS | 7.10 |
| H2-IEd | PNNKEFYEKNLKEYT | 9.40 | DRB1*03:01 | IIADITKNIAGDKID | 7.30 |
| DRB1*07:01 | SGQKLKVVATNSIIA | 0.21 | DRB1*07:01 | LENGIIFAKNIAKQL | 7.30 |
| DRB1*07:01 | TSGQKLKVVATNSII | 0.21 | DRB1*03:01 | LFVESSVDDRPMKTV | 7.30 |
| DRB1*07:01 | GQKLKVVATNSIIAD | 0.27 | DRB1*03:01 | SIIADITKNIAGDKI | 7.30 |
| DRB1*07:01 | QKLKVVATNSIIADI | 0.27 | DRB1*03:01 | VVATNSIIADITKNI | 7.30 |
| DRB1*07:01 | TTSGQKLKVVATNSI | 0.28 | DRB1*11:01 | ENGIIFAKNIAKQLS | 7.40 |
| DRB1*11:01 | GAFKYFSKAYGVPSA | 0.50 | DRB1*07:01 | FKYFSKAYGVPSAYI | 7.40 |
| DRB1*11:01 | EGAFKYFSKAYGVPS | 0.59 | DRB1*07:01 | KYFSKAYGVPSAYIW | 7.40 |
| DRB1*15:01 | SEADLIFYNGINLET | 0.71 | DRB1*07:01 | YFSKAYGVPSAYIWE | 7.40 |
| DRB1*15:01 | KTSEADLIFYNGINL | 0.80 | DRB1*07:01 | FSKAYGVPSAYIWEI | 7.60 |
| DRB1*15:01 | TSEADLIFYNGINLE | 0.82 | DRB1*15:01 | LENGIIFAKNIAKQL | 7.80 |
| DRB1*15:01 | EADLIFYNGINLETG | 0.84 | DRB1*07:01 | NGIIFAKNIAKQLSA | 7.80 |
| DRB1*11:01 | SEGAFKYFSKAYGVP | 0.85 | DRB1*15:01 | SGQKLKVVATNSIIA | 8.10 |
| DRB1*15:01 | ADLIFYNGINLETGG | 1.10 | DRB1*03:01 | SLFVESSVDDRPMKT | 8.20 |
| DRB1*11:01 | TSEGAFKYFSKAYGV | 1.20 | DRB1*13:01 | AFKYFSKAYGVPSAY | 8.30 |
| DRB1*07:01 | KLKVVATNSIIADIT | 1.80 | DRB1*13:01 | EGAFKYFSKAYGVPS | 8.30 |
| DRB1*11:01 | AFKYFSKAYGVPSAY | 1.90 | DRB1*13:01 | FKYFSKAYGVPSAYI | 8.30 |
| DRB1*07:01 | LKVVATNSIIADITK | 2.00 | DRB1*03:01 | FVESSVDDRPMKTVS | 8.30 |
| DRB1*08:01 | ENGIIFAKNIAKQLS | 2.10 | DRB1*13:01 | GAFKYFSKAYGVPSA | 8.30 |
| DRB1*08:01 | GIIFAKNIAKQLSAK | 2.10 | DRB1*15:01 | LNLENGIIFAKNIAK | 8.30 |
| DRB1*08:01 | IFAKNIAKQLSAKDP | 2.10 | DRB1*13:01 | SEGAFKYFSKAYGVP | 8.30 |
| DRB1*08:01 | IIFAKNIAKQLSAKD | 2.10 | DRB1*13:01 | TSEGAFKYFSKAYGV | 8.30 |
| DRB1*08:01 | LENGIIFAKNIAKQL | 2.10 | DRB1*13:01 | VTSEGAFKYFSKAYG | 8.30 |
| DRB1*08:01 | NGIIFAKNIAKQLSA | 2.10 | DRB1*04:01 | DLIFYNGINLETGGN | 8.40 |
| DRB1*08:01 | NLENGIIFAKNIAKQ | 2.10 | DRB1*15:01 | GQKLKVVATNSIIAD | 8.40 |
| DRB1*15:01 | DLIFYNGINLETGGN | 2.30 | DRB1*04:01 | IIFAKNIAKQLSAKD | 8.40 |
| DRB1*11:01 | VTSEGAFKYFSKAYG | 2.30 | DRB1*15:01 | NLENGIIFAKNIAKQ | 8.40 |
| DRB1*08:01 | AFKYFSKAYGVPSAY | 2.40 | DRB1*15:01 | QKLKVVATNSIIADI | 8.40 |
| DRB1*08:01 | EGAFKYFSKAYGVPS | 2.40 | DRB1*04:01 | LIFYNGINLETGGNA | 8.50 |
| DRB1*08:01 | FKYFSKAYGVPSAYI | 2.40 | DRB1*07:01 | AFKYFSKAYGVPSAY | 8.70 |
| DRB1*08:01 | GAFKYFSKAYGVPSA | 2.40 | DRB1*07:01 | EGAFKYFSKAYGVPS | 8.70 |
| DRB1*08:01 | SEGAFKYFSKAYGVP | 2.40 | DRB1*07:01 | GAFKYFSKAYGVPSA | 8.70 |
| DRB1*08:01 | TSEGAFKYFSKAYGV | 2.40 | DRB1*15:01 | ENGIIFAKNIAKQLS | 8.80 |
| DRB1*08:01 | VTSEGAFKYFSKAYG | 2.40 | DRB1*07:01 | GIIFAKNIAKQLSAK | 9.00 |
| DRB1*07:01 | LVEKLRQTKVPSLFV | 2.90 | DRB1*03:01 | SMMKYNLDKIAEGLA | 9.10 |
| DRB1*07:01 | VEKLRQTKVPSLFVE | 2.90 | DRB1*04:01 | TSGQKLKVVATNSII | 9.20 |
| DRB1*11:01 | FKYFSKAYGVPSAYI | 3.30 | DRB1*03:01 | MKTVSQDTNIPIYAQ | 9.30 |
| DRB1*07:01 | EKLRQTKVPSLFVES | 3.50 | DRB1*04:01 | PIYAQIFTDSIAEQG | 9.40 |
| DRB1*15:01 | TSEGAFKYFSKAYGV | 4.20 | DRB1*01:01 | FKYFSKAYGVPSAYI | 9.50 |
| DRB1*15:01 | EGAFKYFSKAYGVPS | 4.30 | DRB1*04:01 | TNSIIADITKNIAGD | 9.60 |
| DRB1*11:01 | GDSYYSMMKYNLDKI | 4.30 | DRB1*04:01 | TTSGQKLKVVATNSI | 9.60 |
| DRB1*15:01 | SEGAFKYFSKAYGVP | 4.30 | DRB1*04:01 | ATNSIIADITKNIAG | 9.70 |
| DRB1*07:01 | KLRQTKVPSLFVESS | 4.40 | DRB1*01:01 | KYFSKAYGVPSAYIW | 9.70 |
| DRB1*11:01 | DSYYSMMKYNLDKIA | 4.50 | DRB1*04:01 | GAFKYFSKAYGVPSA | 9.80 |
| DRB1*07:01 | TLVEKLRQTKVPSLF | 4.50 | DRB1*03:01 | YSMMKYNLDKIAEGL | 9.80 |
| DRB1*11:01 | EGDSYYSMMKYNLDK | 4.60 | DRB1*04:01 | NSIIADITKNIAGDK | 9.90 |
| DRB1*07:01 | KTLVEKLRQTKVPSL | 4.70 | DRB1*11:01 | SYYSMMKYNLDKIAE | 9.90 |
| DRB1*15:01 | GAFKYFSKAYGVPSA | 4.90 | DRB1*04:01 | VATNSIIADITKNIA | 9.90 |
| DRB1*11:01 | KEGDSYYSMMKYNLD | 5.00 | DRB1*11:01 | YYSMMKYNLDKIAEG | 9.90 |
| DRB1*11:01 | NGIIFAKNIAKQLSA | 5.40 |  |  |  |
| **NetMHCIIpan** |  |  |  |  |  |
| **Allele** | **Peptide** | **%Rank** | **Allele** | **Peptide** | **%Rank** |
| H-2-IAb | IYAQIFTDSIAEQGK | 0.89 | DRB1_0701 | SGQKLKVVATNSIIA | 0.47 |
| H-2-IAb | YAQIFTDSIAEQGKE | 0.41 | DRB1_0701 | GQKLKVVATNSIIAD | 0.20 |
| H-2-IAb | AQIFTDSIAEQGKEG | 0.31 | DRB1_0701 | QKLKVVATNSIIADI | 0.38 |
| H-2-IAb | QIFTDSIAEQGKEGD | 0.95 | DRB1_0701 | KLKVVATNSIIADIT | 0.46 |
| H-2-IAd | DKIDLHSIVPIGQDP | 0.37 | DRB1_0701 | NKDYFAVSDGVDVIY | 0.79 |
| H-2-IAd | KIDLHSIVPIGQDPH | 0.27 | DRB1_0701 | VEKLRQTKVPSLFVE | 0.84 |
| H-2-IEd | KIDLHSIVPIGQDPH | 0.57 | DRB1_0801 | TNSIIADITKNIAGD | 0.86 |
| H-2-IEd | LENGIIFAKNIAKQL | 0.55 | DRB1_0801 | NSIIADITKNIAGDK | 0.47 |
| H-2-IEd | ENGIIFAKNIAKQLS | 0.24 | DRB1_0801 | VTSEGAFKYFSKAYG | 0.73 |
| H-2-IEd | NGIIFAKNIAKQLSA | 0.58 | DRB1_0801 | TSEGAFKYFSKAYGV | 0.18 |
| H-2-IEd | GIIFAKNIAKQLSAK | 0.70 | DRB1_0801 | SEGAFKYFSKAYGVP | 0.07 |
| H-2-IEd | ESKDKFNKIPAEKKL | 0.18 | DRB1_0801 | EGAFKYFSKAYGVPS | 0.06 |
| H-2-IEd | SKDKFNKIPAEKKLI | 0.11 | DRB1_0801 | GAFKYFSKAYGVPSA | 0.26 |
| H-2-IEd | KDKFNKIPAEKKLIV | 0.11 | DRB1_0801 | EGDSYYSMMKYNLDK | 0.81 |
| H-2-IEd | SEGAFKYFSKAYGVP | 0.27 | DRB1_0801 | GDSYYSMMKYNLDKI | 0.83 |
| H-2-IEd | EGAFKYFSKAYGVPS | 0.15 | DRB1_1101 | TNSIIADITKNIAGD | 0.60 |
| DRB1_0301 | VATNSIIADITKNIA | 0.93 | DRB1_1101 | NSIIADITKNIAGDK | 0.27 |
| DRB1_0301 | ATNSIIADITKNIAG | 0.45 | DRB1_1101 | VTSEGAFKYFSKAYG | 1.0 |
| DRB1_0301 | TNSIIADITKNIAGD | 0.29 | DRB1_1101 | TSEGAFKYFSKAYGV | 0.18 |
| DRB1_0301 | NSIIADITKNIAGDK | 0.72 | DRB1_1101 | SEGAFKYFSKAYGVP | 0.07 |
| DRB1_0301 | MKTVSQDTNIPIYAQ | 0.95 | DRB1_1101 | EGAFKYFSKAYGVPS | 0.06 |
| DRB1_0301 | IYAQIFTDSIAEQGK | 0.92 | DRB1_1101 | GAFKYFSKAYGVPSA | 0.20 |
| DRB1_0301 | YAQIFTDSIAEQGKE | 0.66 | DRB1_1301 | TNSIIADITKNIAGD | 0.61 |
| DRB1_0401 | VDVIYLEGQNEKGKE | 0.95 | DRB1_1301 | NSIIADITKNIAGDK | 0.31 |
| DRB1_0401 | DVIYLEGQNEKGKED | 0.60 | DRB1_1301 | ENGIIFAKNIAKQLS | 0.97 |
| DRB1_0401 | VPSAYIWEINTEEEG | 0.98 | DRB1_1301 | PEQIKTLVEKLRQTK | 0.75 |
| DRB1_0401 | PSAYIWEINTEEEGT | 0.79 | DRB1_1501 | GQKLKVVATNSIIAD | 0.88 |
| DRB1_0401 | YAQIFTDSIAEQGKE | 0.63 | DRB1_1501 | TSEADLIFYNGINLE | 0.31 |
| DRB1_0401 | AQIFTDSIAEQGKEG | 0.80 | DRB1_1501 | SEADLIFYNGINLET | 0.17 |
| DRB1_0401 | SMMKYNLDKIAEGLA | 0.61 | DRB1_1501 | EADLIFYNGINLETG | 0.10 |
| DRB1_0401 | MMKYNLDKIAEGLAK | 0.29 | DRB1_1501 | ADLIFYNGINLETGG | 0.41 |
| DRB1_0701 | TSGQKLKVVATNSII | 0.92 | DRB1_1501 |  |  |

**Supplementary Table 10.** Prediction of MHCII epitopes from PspC by IEDB and NetMHCIIpan.

| IEDB | | |  | | | |
| --- | --- | --- | --- | --- | --- | --- |
| Allele | **Peptide** | **Percentile Rank** | **Allele** | **Peptide** | **Percentile Rank** | |
| H2-IEd | LAFNIKLSRIKTEYL | 4.75 | DRB1*11:01 | HTQNLAFNIKLSRIK | 5.40 | |
| H2-IEd | NLAFNIKLSRIKTEY | 4.80 | DRB1*07:01 | NIKLSRIKTEYLNGL | 5.50 | |
| H2-IEd | QNLAFNIKLSRIKTE | 4.95 | DRB1*11:01 | AFNIKLSRIKTEYLN | 5.80 | |
| H2-IAd | KAKVESKKAVATRLE | 6.00 | DRB1*07:01 | FNIKLSRIKTEYLNG | 5.80 | |
| H2-IEd | DAAFKQFKKDTLPTE | 6.15 | DRB1*03:01 | IQLDKRKHTQNLAFN | 6.10 | |
| H2-IEd | ELDAAFKQFKKDTLP | 6.15 | DRB1*15:01 | AFNIKLSRIKTEYLN | 6.60 | |
| H2-IEd | LDAAFKQFKKDTLPT | 6.20 | DRB1*03:01 | ATRLENIKTDRKKAE | 7.00 | |
| H2-IEd | TQNLAFNIKLSRIKT | 6.25 | DRB1*03:01 | ATRLENIKTDRKKAE | 7.00 | |
| H2-IEd | AELDAAFKQFKKDTL | 7.45 | DRB1*15:01 | IKLSRIKTEYLNGLK | 7.00 | |
| H2-IAd | AKAKVESKKAVATRL | 7.85 | DRB1*07:01 | IKLSRIKTEYLNGLK | 7.10 | |
| H2-IAd | QAKAKVESKKAVATR | 7.85 | DRB1*13:01 | DEYINKMIQLDKRKH | 7.50 | |
| H2-IAd | AKVESKKAVATRLEN | 8.10 | DRB1*13:01 | EYINKMIQLDKRKHT | 7.50 | |
| H2-IEd | AFNIKLSRIKTEYLN | 8.20 | DRB1*15:01 | HTQNLAFNIKLSRIK | 7.50 | |
| H2-IEd | HTQNLAFNIKLSRIK | 9.15 | DRB1*13:01 | INKMIQLDKRKHTQN | 7.50 | |
| H2-IEd | YINKMIQLDKRKHTQ | 9.65 | DRB1*13:01 | KMIQLDKRKHTQNLA | 7.50 | |
| H2-IAd | KQAKAKVESKKAVAT | 9.90 | DRB1*13:01 | MIQLDKRKHTQNLAF | 7.50 | |
| DRB1*03:01 | NKMIQLDKRKHTQNL | 0.95 | DRB1*13:01 | NKMIQLDKRKHTQNL | 7.50 | |
| DRB1*03:01 | INKMIQLDKRKHTQN | 0.96 | DRB1*13:01 | YINKMIQLDKRKHTQ | 7.50 | |
| DRB1*03:01 | KMIQLDKRKHTQNLA | 1.20 | DRB1*03:01 | QNLAFNIKLSRIKTE | 7.60 | |
| DRB1*03:01 | YINKMIQLDKRKHTQ | 1.20 | DRB1*15:01 | TQNLAFNIKLSRIKT | 7.60 | |
| DRB1*08:01 | AEQFDEYINKMIQLD | 1.40 | DRB1*03:01 | ENIKTDRKKAEEEAK | 8.10 | |
| DRB1*08:01 | DEYINKMIQLDKRKH | 1.40 | DRB1*03:01 | ENIKTDRKKAEEEAK | 8.10 | |
| DRB1*08:01 | EQFDEYINKMIQLDK | 1.40 | DRB1*15:01 | NIKLSRIKTEYLNGL | 8.10 | |
| DRB1*08:01 | EYINKMIQLDKRKHT | 1.40 | DRB1*04:01 | AAFKQFKKDTLPTEP | 8.40 | |
| DRB1*08:01 | FDEYINKMIQLDKRK | 1.40 | DRB1*08:01 | AFNIKLSRIKTEYLN | 8.40 | |
| DRB1*08:01 | QFDEYINKMIQLDKR | 1.40 | DRB1*08:01 | FNIKLSRIKTEYLNG | 8.40 | |
| DRB1*08:01 | YINKMIQLDKRKHTQ | 1.40 | DRB1*08:01 | HTQNLAFNIKLSRIK | 8.40 | |
| DRB1*11:01 | INKMIQLDKRKHTQN | 1.70 | DRB1*08:01 | LAFNIKLSRIKTEYL | 8.40 | |
| DRB1*11:01 | NKMIQLDKRKHTQNL | 1.70 | DRB1*08:01 | NLAFNIKLSRIKTEY | 8.40 | |
| DRB1*11:01 | YINKMIQLDKRKHTQ | 1.80 | DRB1*08:01 | QNLAFNIKLSRIKTE | 8.40 | |
| DRB1*11:01 | KMIQLDKRKHTQNLA | 2.40 | DRB1*08:01 | TQNLAFNIKLSRIKT | 8.40 | |
| DRB1*03:01 | EYINKMIQLDKRKHT | 2.60 | DRB1*11:01 | MIQLDKRKHTQNLAF | 8.80 | |
| DRB1*11:01 | EYINKMIQLDKRKHT | 2.60 | DRB1*03:01 | TQNLAFNIKLSRIKT | 8.80 | |
| DRB1*11:01 | QNLAFNIKLSRIKTE | 3.00 | DRB1*04:01 | FKQFKKDTLPTEPEK | 8.90 | |
| DRB1*11:01 | NLAFNIKLSRIKTEY | 3.20 | DRB1*03:01 | HTQNLAFNIKLSRIK | 8.90 | |
| DRB1*07:01 | LAFNIKLSRIKTEYL | 3.60 | DRB1*03:01 | KHTQNLAFNIKLSRI | 9.10 | |
| DRB1*07:01 | AFNIKLSRIKTEYLN | 3.70 | DRB1*04:01 | AFKQFKKDTLPTEPE | 9.20 | |
| DRB1*11:01 | TQNLAFNIKLSRIKT | 3.70 | DRB1*15:01 | FNIKLSRIKTEYLNG | 9.20 | |
| DRB1*03:01 | MIQLDKRKHTQNLAF | 3.80 | DRB1*08:01 | INKMIQLDKRKHTQN | 9.20 | |
| DRB1*11:01 | LAFNIKLSRIKTEYL | 4.10 | DRB1*08:01 | KMIQLDKRKHTQNLA | 9.20 | |
| DRB1*03:01 | RLENIKTDRKKAEEE | 4.30 | DRB1*08:01 | MIQLDKRKHTQNLAF | 9.20 | |
| DRB1*03:01 | RLENIKTDRKKAEEE | 4.30 | DRB1*08:01 | NKMIQLDKRKHTQNL | 9.20 | |
| DRB1*03:01 | TRLENIKTDRKKAEE | 4.60 | DRB1*15:01 | LAFNIKLSRIKTEYL | 9.30 | |
| DRB1*03:01 | TRLENIKTDRKKAEE | 4.60 | DRB1*04:01 | KQFKKDTLPTEPEKK | 9.50 | |
| DRB1*11:01 | DEYINKMIQLDKRKH | 4.90 | DRB1*03:01 | RKHTQNLAFNIKLSR | 9.50 | |
| DRB1*03:01 | LENIKTDRKKAEEEA | 5.20 | DRB1*11:01 | FNIKLSRIKTEYLNG | 9.60 | |
| DRB1*03:01 | LENIKTDRKKAEEEA | 5.20 |  |  |  | |
| NetMHCIIpan |  |  |  |  |  |  |
| Allele | **Peptide** | **%Rank** | **Allele** | **Peptide** | **%Rank** |  |
| H-2-IAb | KVKEKPAEQPQPAPA | 0.38 | H-2-IEd | VKKAEFELVKEEAKE | 0.94 |  |
| H-2-IAb | VKEKPAEQPQPAPAP | 0.07 | H-2-IEd | KKAEFELVKEEAKEP | 0.19 |  |
| H-2-IAb | KEKPAEQPQPAPAPQ | 0.04 | DRB1_0301 | YINKMIQLDKRKHTQ | 0.78 |  |
| H-2-IAb | EKPAEQPQPAPAPQP | 0.03 | DRB1_0301 | INKMIQLDKRKHTQN | 0.49 |  |
| H-2-IAb | KPAEQPQPAPAPQPE | 0.11 | DRB1_0301 | NKMIQLDKRKHTQNL | 0.54 |  |
| H-2-IAd | KVEEAEKKAKAQKEE | 0.72 | DRB1_0301 | TRLENIKTDRKKAEE | 0.30 |  |
| H-2-IAd | VEEAEKKAKAQKEED | 0.27 | DRB1_0301 | RLENIKTDRKKAEEE | 0.14 |  |
| H-2-IAd | EEAEKKAKAQKEEDR | 0.16 | DRB1_0301 | LENIKTDRKKAEEEA | 0.10 |  |
| H-2-IAd | EAEKKAKAQKEEDRR | 0.62 | DRB1_0301 | ENIKTDRKKAEEEAK | 0.21 |  |
| H-2-IAd | ELEIAEFDVEVKKAE | 0.59 | DRB1_0301 | FELVKEEAKEPRNEE | 0.85 |  |
| H-2-IAd | LEIAEFDVEVKKAEL | 0.48 | DRB1_0301 | TRLENIKTDRKKAEE | 0.30 |  |
| H-2-IAd | EIAEFDVEVKKAELE | 0.24 | DRB1_0301 | RLENIKTDRKKAEEE | 0.14 |  |
| H-2-IAd | KVEEAEKKAKAQKEE | 0.72 | DRB1_0301 | LENIKTDRKKAEEEA | 0.10 |  |
| H-2-IAd | VEEAEKKAKAQKEED | 0.27 | DRB1_0301 | ENIKTDRKKAEEEAK | 0.21 |  |
| H-2-IAd | EEAEKKAKAQKEEDR | 0.16 | DRB1_0801 | QFDEYINKMIQLDKR | 0.44 |  |
| H-2-IAd | EAEKKAKAQKEEDRR | 0.62 | DRB1_0801 | FDEYINKMIQLDKRK | 0.27 |  |
| H-2-IAd | ELEISESDVEVKKAE | 0.61 | DRB1_0801 | DEYINKMIQLDKRKH | 0.50 |  |
| H-2-IAd | LEISESDVEVKKAEF | 0.32 | DRB1_0801 | EKKVAEAQKKVEEAE | 0.83 |  |
| H-2-IAd | EISESDVEVKKAEFE | 0.15 | DRB1_0801 | EIAEFDVEVKKAELE | 0.57 |  |
| H-2-IAd | EEKVKQAKAKVESKK | 0.72 | DRB1_0801 | IAEFDVEVKKAELEL | 0.69 |  |
| H-2-IEd | TQNLAFNIKLSRIKT | 0.74 | DRB1_1301 | INKMIQLDKRKHTQN | 0.63 |  |
| H-2-IEd | QNLAFNIKLSRIKTE | 0.26 | DRB1_1301 | NKMIQLDKRKHTQNL | 0.96 |  |
| H-2-IEd | NLAFNIKLSRIKTEY | 0.26 | DRB1_1301 | HTQNLAFNIKLSRIK | 0.57 |  |
| H-2-IEd | ATRLENIKTDRKKAE | 0.75 | DRB1_1301 | TQNLAFNIKLSRIKT | 0.46 |  |
